# Supplementary material for: APOE from astrocytes restores Alzheimer’s Aβ-pathology and DAM-like responses in APOE deficient microglia
Source: EMBO Mol Med. 2024 Nov 11;16(12):3113–41. doi: 10.1038/s44321-024-00162-7 (PMC11628604; doi:10.1038/s44321-024-00162-7)
Supplement: Supplementary file 1 — Appendix [file 44321_2024_162_MOESM1_ESM.pdf]

Table of Contents

1. Appendix Figure S1: AAV-mediated transduction enables expression of APOE specifically in astrocytes in vivo. **2**

2. Appendix Figure S2: Astrocyte-derived APOE leads to fibrillar Aβ plaque formation in vivo. **4**

3. Appendix Figure S3: APOE expression modulates astrocyte cell-states in *App*$^{NL-G-F}$ mouse brain. **5**

4. Appendix Figure S4: *Apoe*-deficient microglia mount reactive responses to fibrillar amyloid plaques. **7**

5. Appendix Figure S5: APOE levels and microglia depletion. **9**

6. Appendix Figure S6: Quality control of microglia and astrocyte single cell libraries. **10**

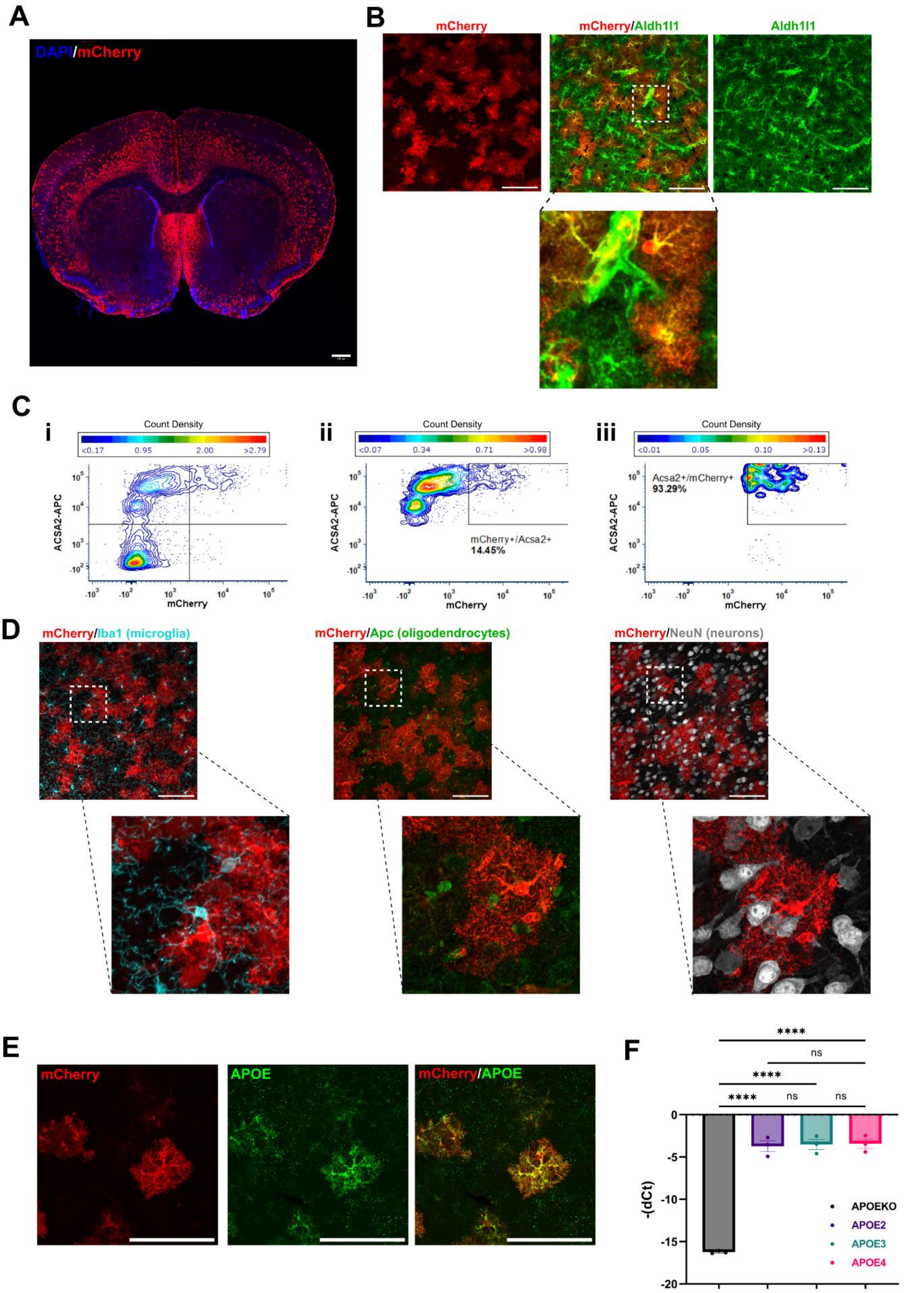

Appendix Figure S1. AAV-mediated transduction enables expression of APOE specifically in astrocytes in vivo. (A) IF image of AAV transduced mouse brain coronal sections at 2 months of age showing distribution of transduced astrocytes (mCherry in red) in cortex and subcortical areas (in *App*$^{NL-G-F}$ x *Rag2*$^{-/-}$ x *Apoe*$^{-/-}$ mice. Scale bar: 500 µm. **(B)** IF image of AAV transduced cortical region (see

Appendix Fig. S2A) of 6 months old *App*$^{NL-G-F}$ x Apoe$^{-/-}$ mouse brain, showing mCherry (red) co-localizing with astrocyte marker Aldh1l1 (green). Image below shows a zoomed (5x) view of merged image indicated by the inset box of white dashed-lines. Scale bar: 100 µm. **(C)** Contour plot from flow cytometry analysis showing (i) density distribution of mCherry+ and Acsa2+ astrocytes, split to show (ii) proportion of mCherry+ cells in total Acsa2+ cells, and (iii) proportion of Acsa2+ astrocytes in total mCherry+ cells, in 6 months old *App*$^{NL-G-F}$ x Apoe$^{-/-}$ mouse brain. Colour scale indicates count density for each contour bin. **(D)** IF images of AAV transduced cortical region (see Appendix Fig. S2A) of 6 months old *App*$^{NL-G-F}$ x *Apoe*$^{-/-}$ mouse brain. mCherry (red) shows the transduced cells. Co-staining with anti-Iba1 antibody showing microglia (cyan) (Note that this is the same image as S1B and co-staining was done with Aldh1l1); with anti-Apc antibody showing oligodendrocytes cells (green); with anti-NeuN antibody showing neurons (white) (Note that Apc and NeuN panels are shown from the same image as these markers were co-stained in the same slide). Images below show zoomed in (5x) views indicated by the inset box of white dashed-lines. Scale bar: 100 µm. **(E)** IF images of AAV transduced cortical region (see Appendix Fig. S2A) of 6 months old *App*$^{NL-G-F}$ x *Rag2*$^{-/-}$ x *Apoe*$^{-/-}$ mouse brain, showing APOE3 (green) co-localizing with mCherry+ (red). Scale bar: 100 µm. **(F)** Bar plot showing human APOE mRNA levels at 6 months of age in mouse brain homogenates measured by semiquantitative real-time PCR. Data points show mean value for 3 technical replicates per mouse (n= 3 mice per group). ns = non-significant. **Statistical tests:** Data presented as mean ± SEM in (F). One-way ANOVA and Tukey's multiple comparison test (F). Significance shown for pairwise comparisons: ****p < 0.0001

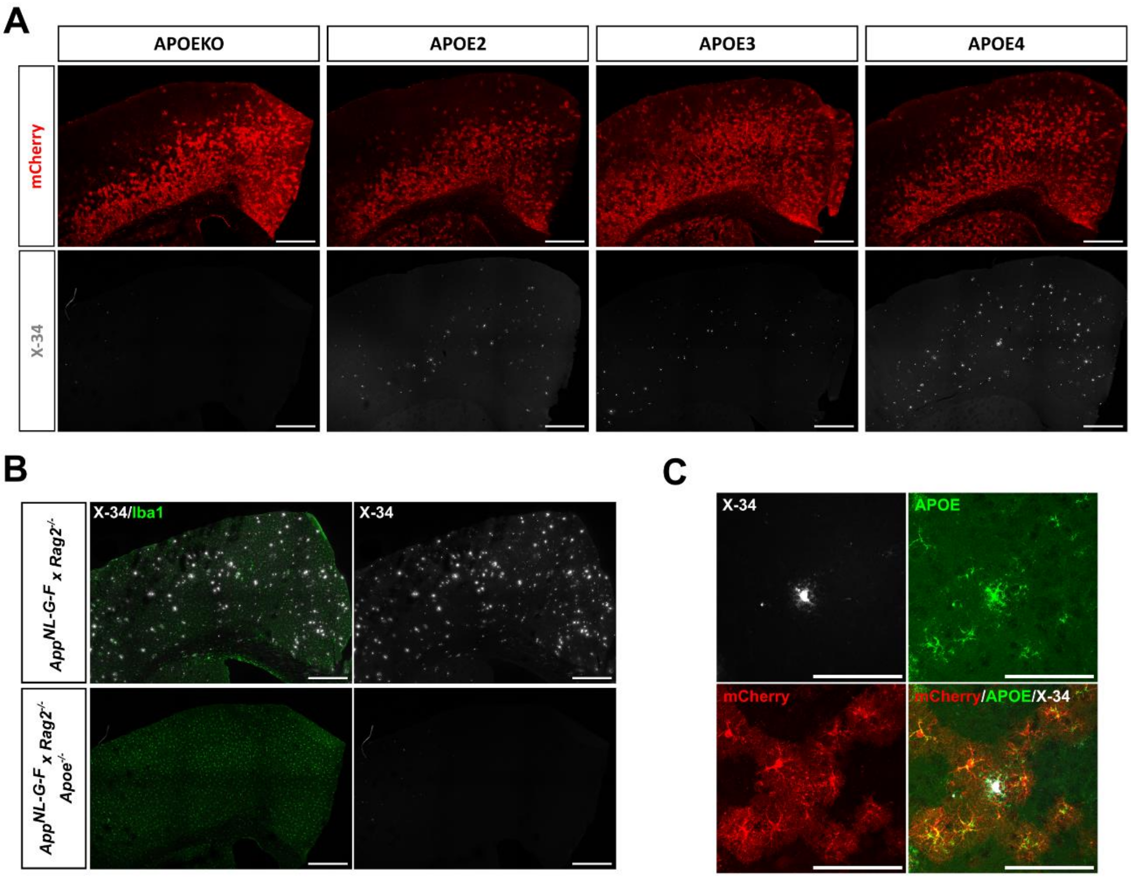

Appendix Figure S2. Astrocyte-derived APOE leads to fibrillar Aβ plaque formation in vivo. (A) IF images showing AAV transduced (red) regions in the cortex and the associated distribution of X-34+ fibrillar plaque deposits (white) in 6 months old *App*$^{NL\text{-}G\text{-}F}$ x *Rag2*$^{-/-}$ x Apoe$^{-/-}$ mice. Scale bar: 500 µm **(B)** IF images showing distribution of X-34+ fibrillar plaque deposits (white) and Iba1+ microglia clustering (green) in cortical region in 6 months old *App*$^{NL\text{-}G\text{-}F}$ x *Rag2*$^{-/-}$ mice (top panel). Absence of fibrillar plaque deposits and clustered microglia in cortical region of 6 months old *App*$^{NL\text{-}G\text{-}F}$ x *Rag2*$^{-/-}$ x Apoe$^{-/-}$ mouse brain (bottom panel). Scale bar: 500 µm. **(C)** IF images of AAV transduced cortical region of 6 months old *App*$^{NL\text{-}G\text{-}F}$ x *Apoe*$^{-/-}$ mouse brain, showing APOE3 (green) co-localizing with mCherry+ (red) astrocytes and X-34+ (white) fibrillar plaque deposits. Scale bar: 100 µm.

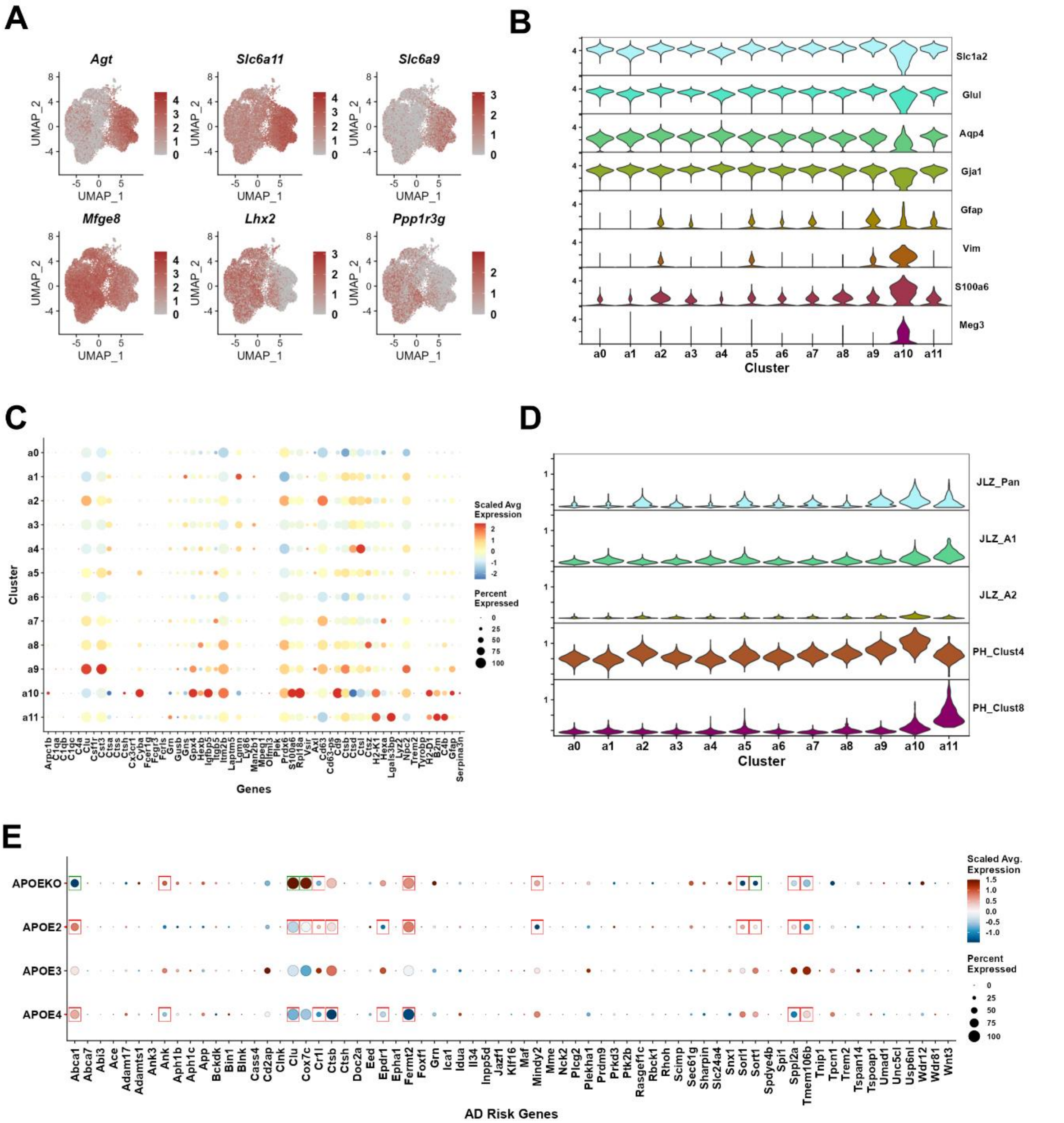

Appendix Figure S3. APOE expression modulates astrocyte cell-states in *App*$^{NL-G-F}$ mouse brain. (A) UMAP plot (same as Figure 6a), showing expression of markers for non-telencephalon (*Agt, Slc6a11, Slac6a9*) and telencephalon (*Mfge8, Lhx2, Ppp1r3g*) astrocytes. Colour scale indicates normalized gene expression. **(B)** Violin plot showing normalized module score in each cluster, for marker gene set from previously identified reactive astrocyte cell-states. JLZ_Pan- Pan-reactive astrocytes, JLZ_A1- A1 astrocytes, JLZ_A2- A2 astrocytes from (Zamanian *et al.*, 2012), PH_Clust4- Cluster 4, PH_Clust- Cluster 8 from (Hasel *et al.*, 2021). **(C)** Violin plot showing normalized gene expression in each cluster, for homeostatic astrocyte markers (*Slc1a2, Glul, Aqp4, Gja1*) and reactive astrocyte markers (*Gfap, Vim, S100a6, Meg3*) **(D)** Dot plot showing expression of previously identified Plaque Induced Genes (PIGs) (Chen *et al.*, 2020) in each cluster. Colour scale indicates normalized expression level, scaled per gene (z-score). Dot size indicates percentage of cells, in each group, expressing the gene. **(E)** Dot plot showing expression of previously identified AD risk genes, split by experimental groups. Colour scale indicates normalized expression level, scaled per gene (z-score). Dot size indicates percentage of cells, in each group, expressing the gene. Squares around dots mark statistically significant genes (expressed

in more than 25% of cells in each group and with adjusted p-value < 0.05) based on differential expression against APOE3 group. Green squares indicate genes with | Log2(Fold Change) | > 0.2. Red squares indicate genes with | Log2(Fold Change) | < 0.2. **Statistical tests:** MAST differential expression test in (E), p-values were adjusted with Bonferroni correction based on the total number of genes in the dataset.

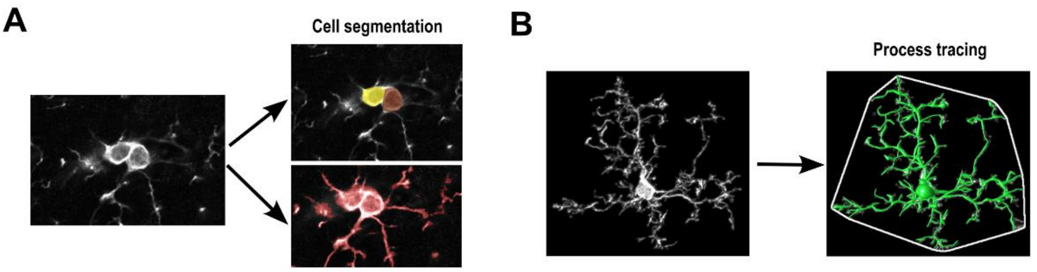

C

	Non-plaque-associated microglia	Plaque-associated microglia	Total number of microglia
APOEKO	1446	11	**1457**
APOE2	1077	456	**1533**
APOE3	1408	186	**1594**
APOE4	1181	1160	**2341**
**All groups**	**5112**	**1813**	**6925**

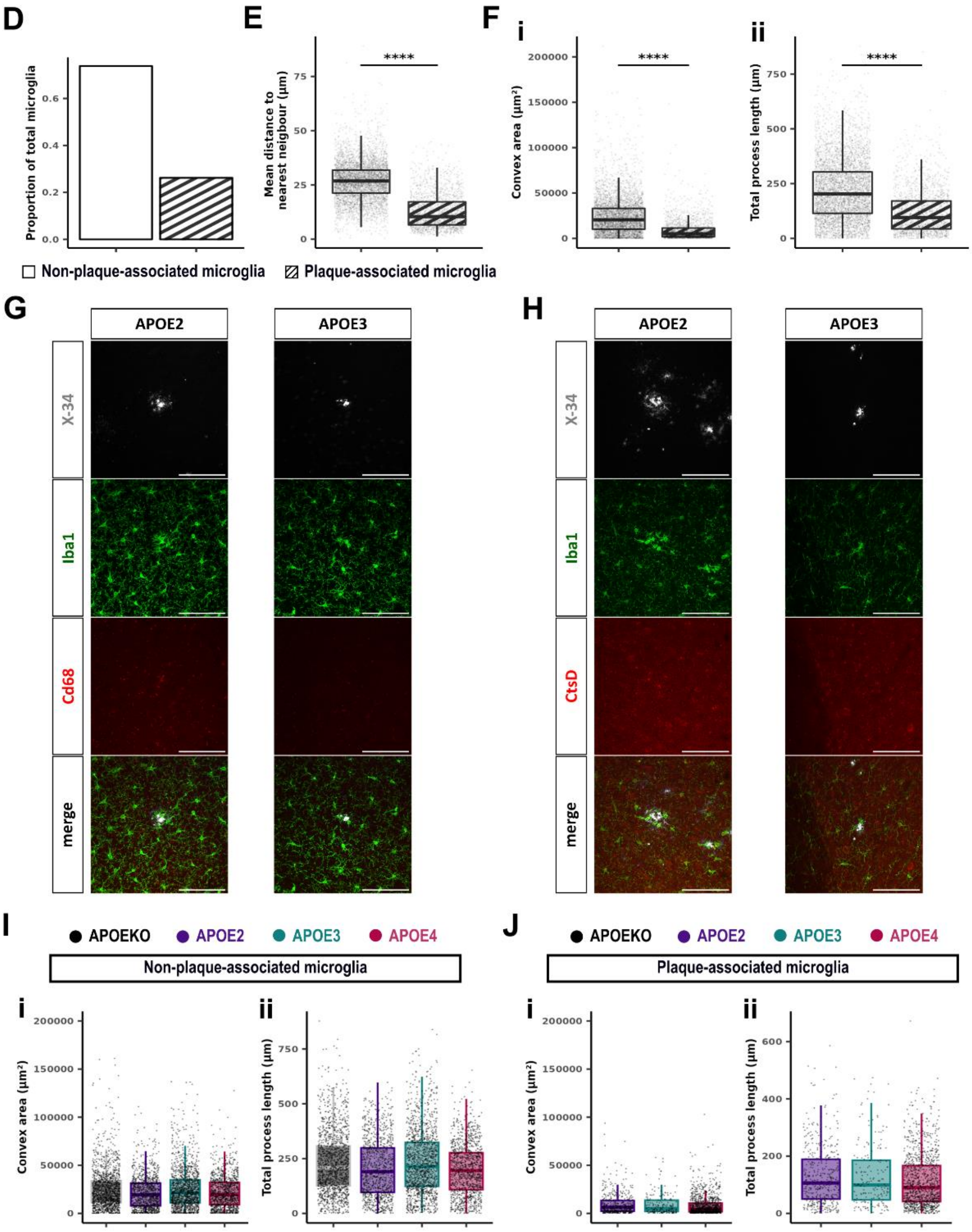

Appendix Figure S4. ***Apoe*****-deficient microglia mount reactive responses to fibrillar amyloid plaques.** **(A)** Representation of thresholding (first step) and segmentation of microglia cell bodies, based on anti-Iba1 immunostaining, using a combination of Cellpose (second step: top panel) and Labkit (second step: bottom panel) algorithms. See Materials and Methods for details. **(B)** Representation of thresholding (first step) and tracing (second step) of primary and secondary processes of segmented microglial cell. Sum of the length of primary and secondary process calculated as "total process length". Area of bounding box (in white) calculated as "convex area" for territory size. **(C)** Number of microglia segmented and quantified from AAV transduced cortical regions (40x magnification), at 6 months of age, from the experimental groups, further classified as plaque-associated (within X34+ thresholded plaque area and up to 5 µm outside a plaque's edge) or non-plaque-associated. (n= 6-7 mice per group. 3 fields of view per mouse). **(D)** Bar plot showing proportion of total number of microglia from all the experimental groups (Non-plaque-associated microglia = 0.74; Plaque-associated microglia = 0.26). **(E)** Box plot showing mean distance between each microglia to its nearest three neighbours, for microglial cells classified as plaque-associated or non-plaque-associated. Data points show individual microglia from all experimental groups. **(F)** Box plots showing (i) territory size (convex area) occupied by, and (ii) total process length of, microglia classified as plaque-associated or non-plaque-associated. Data points show individual microglia from all experimental groups. Texture legends for microglia classification in (D), (E) and (F) are indicated. **(G)** IF images of AAV-APOE2 and AAV-APOE3 transduced cortical regions (see Appendix Fig. S2A) at 6 months of age. X-34 staining (white) shows the fibrillar plaque deposits. Co-staining with anti-Iba1 antibody shows microglial cells (green) and anti-Cd68 antibody shows phagocytic structures (red) inside microglia surrounding plaques. Scale bar: 100 µm. **(H)** IF images of AAV-APOE2 and AAV-APOE3 transduced cortical regions (see Appendix Fig. S2A) at 6 months of age. X-34 staining (white) shows the fibrillar plaque deposits. Co-staining with anti-Iba1 antibody shows microglia (green) and anti-CtsD antibody shows lysosomal structures (red) inside clustered microglia surrounding plaques. Scale bar: 100 µm. **(I)** Box plots showing (i) territory size (convex area) occupied by, and (ii) total process lengths of, non-plaque-associated microglial cells compared between the experimental groups. Data points show individual microglia from each experimental group. **(J)** Box plots showing (i) territory size (convex area) occupied by, and (ii) total process lengths of, plaque-associated microglial cells compared between experimental groups. Data points show individual microglia from each experimental group. Colours legends for experimental groups in (G) and (H) are indicated. **Statistical tests:** Data represented as median and interquartile range ± values within 1.5 times the interquartile range in (D), (E), (F), (I) and (J). Linear mixed effects model in (C-E). Linear mixed effects model with Tukey's HSD test in (F), (I) and (J). Significance shown for pairwise comparisons ****p < 0.0001

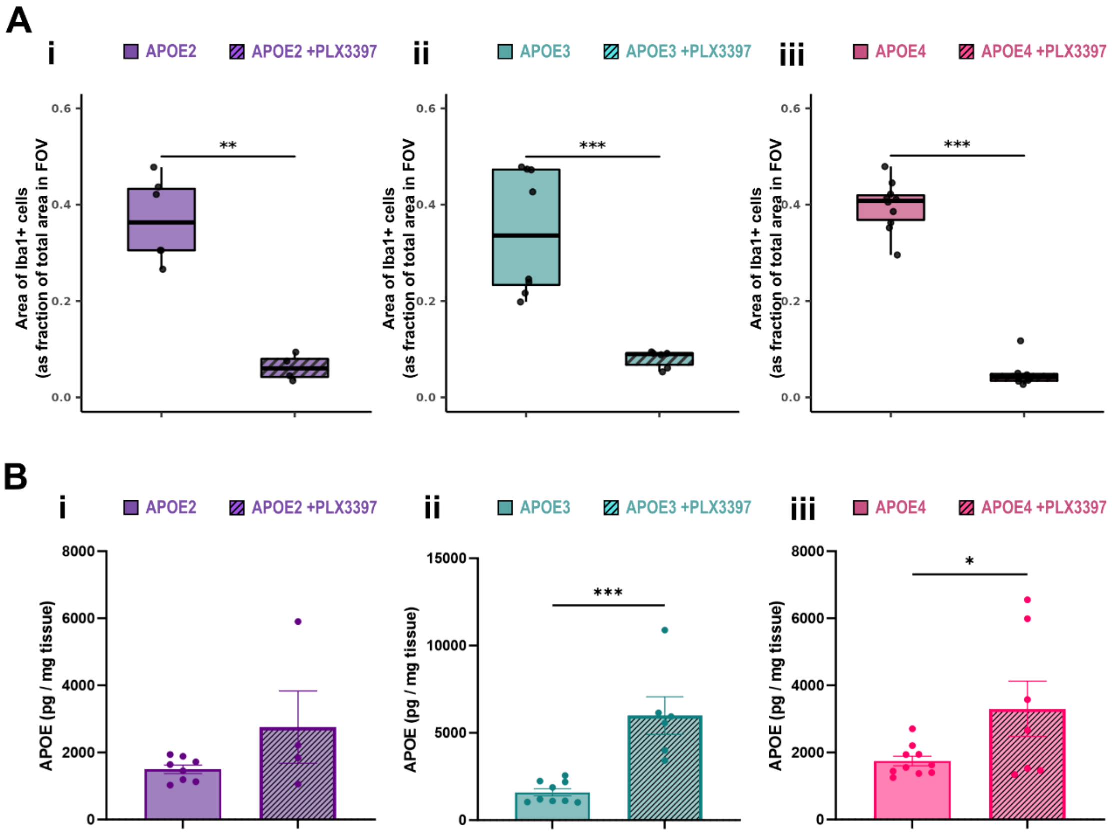

Appendix Figure S5. APOE levels and microglia depletion. (A) Box plots showing area of Iba1+ cells (as fraction of total area in FOV) in AAV transduced cortical regions at 6 months of age, from control and PLX3397-treated (shaded) groups in (i) APOE2, (ii) APOE3, (iii) APOE4. Percentage reductions are: APOE2: 83%, APOE3: 77%, APOE4: 87%. Data points show mean value for 3 FOV per mouse (n= 4-10 mice per group). **(B)** Bar plots showing total APOE levels in brain homogenate from control and PLX3397-treated (shaded) groups in (i) APOE2, (ii) APOE3, (iii) APOE4, measured by MSD-ELISA. Data points show mean value for 2 technical replicates per mouse (n= 4-10 mice per group). **Statistical tests:** Data represented as median and interquartile range ± values within 1.5 times the interquartile range in (A); mean ± SEM in (B). Non-parametric Wilcoxon test in (a). Unpaired t-test in (b). $*p < 0.05$; $**p < 0.01$; $***p < 0.001$

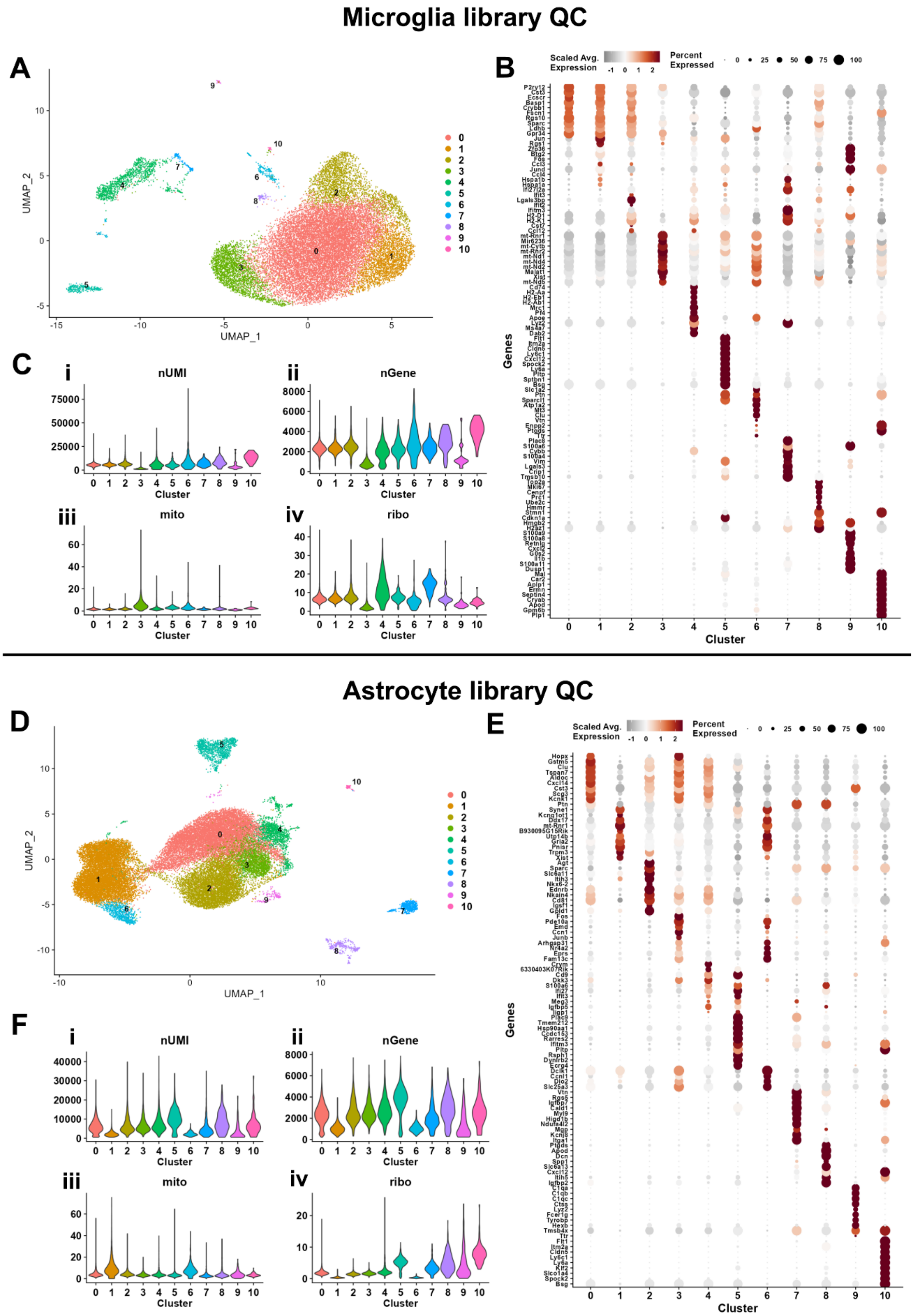

Appendix Figure S6: Quality control of microglia and astrocyte single cell libraries. (A) UMAP plot showing 23,495 APOE-deficient microglia (Cd11b+/Cd45-low) sorted from 6 months old mouse brains from the four experimental groups (n= 2 or 3 mice per group). **(B)** Dot plot showing the top 10

differentially expressed genes in each QC cluster of microglia library. Colour scale indicates normalized expression level, scaled by gene (z-score). Dot size indicates percentage of cells, in each cluster, expressing the gene. Microglia QC Clusters 0, 1, 2, 3 and 8 have microglial identity. Contaminating cell types identified are macrophages (Microglia QC Cluster 4), endothelial cells (Microglia QC Cluster 5), mix of astrocyte and microglial cells (Microglia QC Cluster 6), monocytes (Microglia QC Clusters 7 and 9) and oligodendrocytes (QC Cluster 10). **(C)** Violin plot showing (i) nUMI (total count of RNA transcripts captured), (ii) nGene (total number of genes), (iii) mito (percentage of mitochondrial genes), (iv) ribo (percentage of ribosomal genes), in each microglial QC cluster. **(D)** UMAP plot showing 32,982 astrocytes (mCherry+/Acsa2+) sorted from 6 months old mouse brains from the four experimental groups (n= 2 or 3 mice per group). **(E)** Dot plot showing the top 10 differentially expressed genes in each QC cluster of astrocyte library. Colour scale indicates normalized expression level, scaled by gene (z-score). Dot size indicates percentage of cells, in each cluster, expressing the gene. Astrocyte QC Clusters 0, 1, 2, 3, 4 and 6 have astrocyte identity. Contaminating cell types identified are ependymal cells (Astrocyte QC Cluster 5), mural cells (Astrocyte QC Cluster 7), microglia (Astrocyte QC Cluster 9), endothelial cells (Astrocyte QC Cluster 10) and a population of undetermined identity (Astrocyte QC Cluster 8). **(F)** Violin plot showing (i) nUMI (total count of RNA transcripts captured), (ii) nGene (total number of genes), (iii) mito (percentage of mitochondrial genes), (iv) ribo (percentage of ribosomal genes), in each astrocyte QC cluster.
