# Supplementary figures and images for: APOE from astrocytes restores Alzheimer’s Aβ-pathology and DAM-like responses in APOE deficient microglia
Source: EMBO Mol Med. 2024 Nov 11;16(12):3113–41. doi: 10.1038/s44321-024-00162-7 (PMC11628604; doi:10.1038/s44321-024-00162-7)

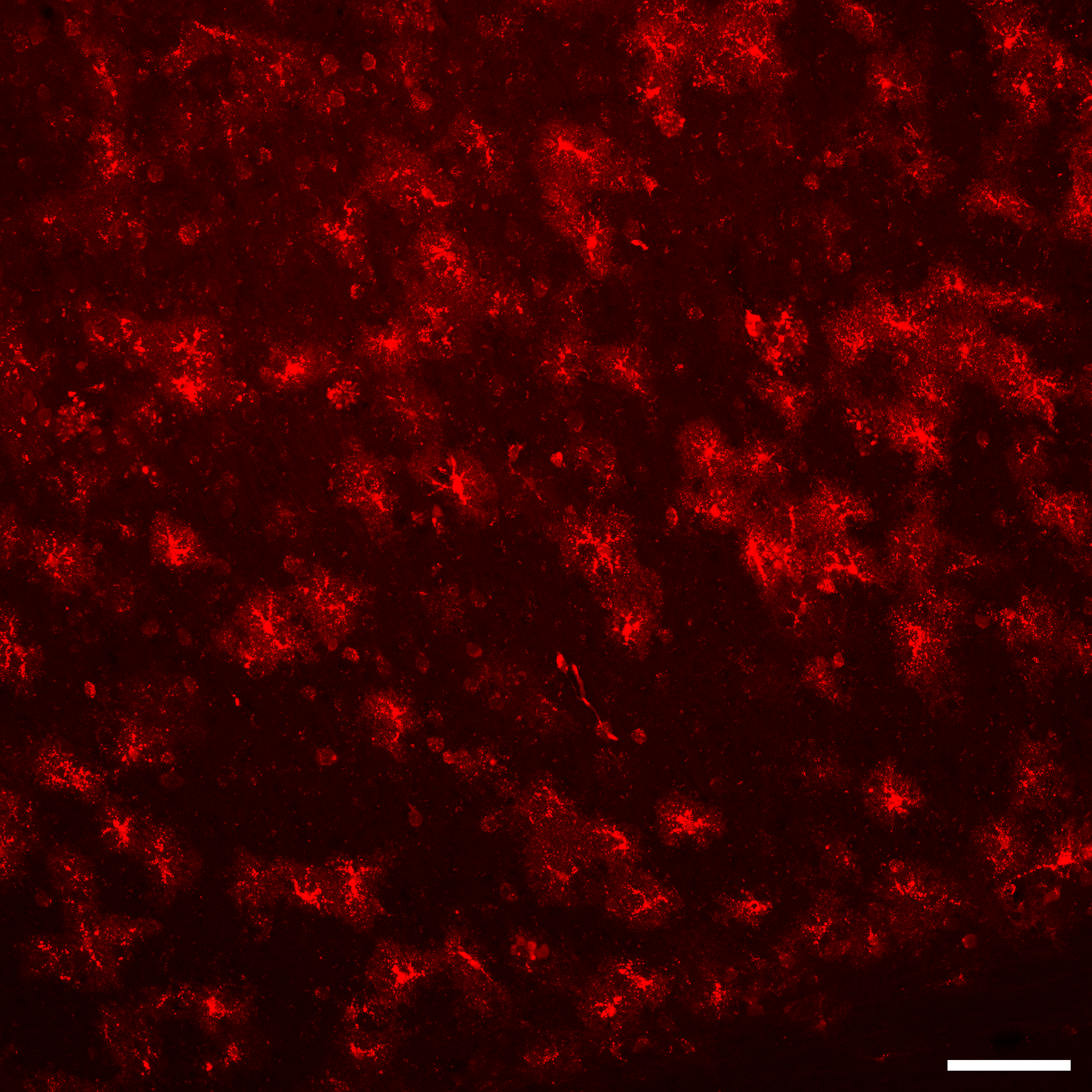

Supplement: Supplementary file 9 — Source data Fig. 1 [file 44321_2024_162_MOESM9_ESM.zip › Figure 1/1B/1B.APOE2_mcherry.tif]

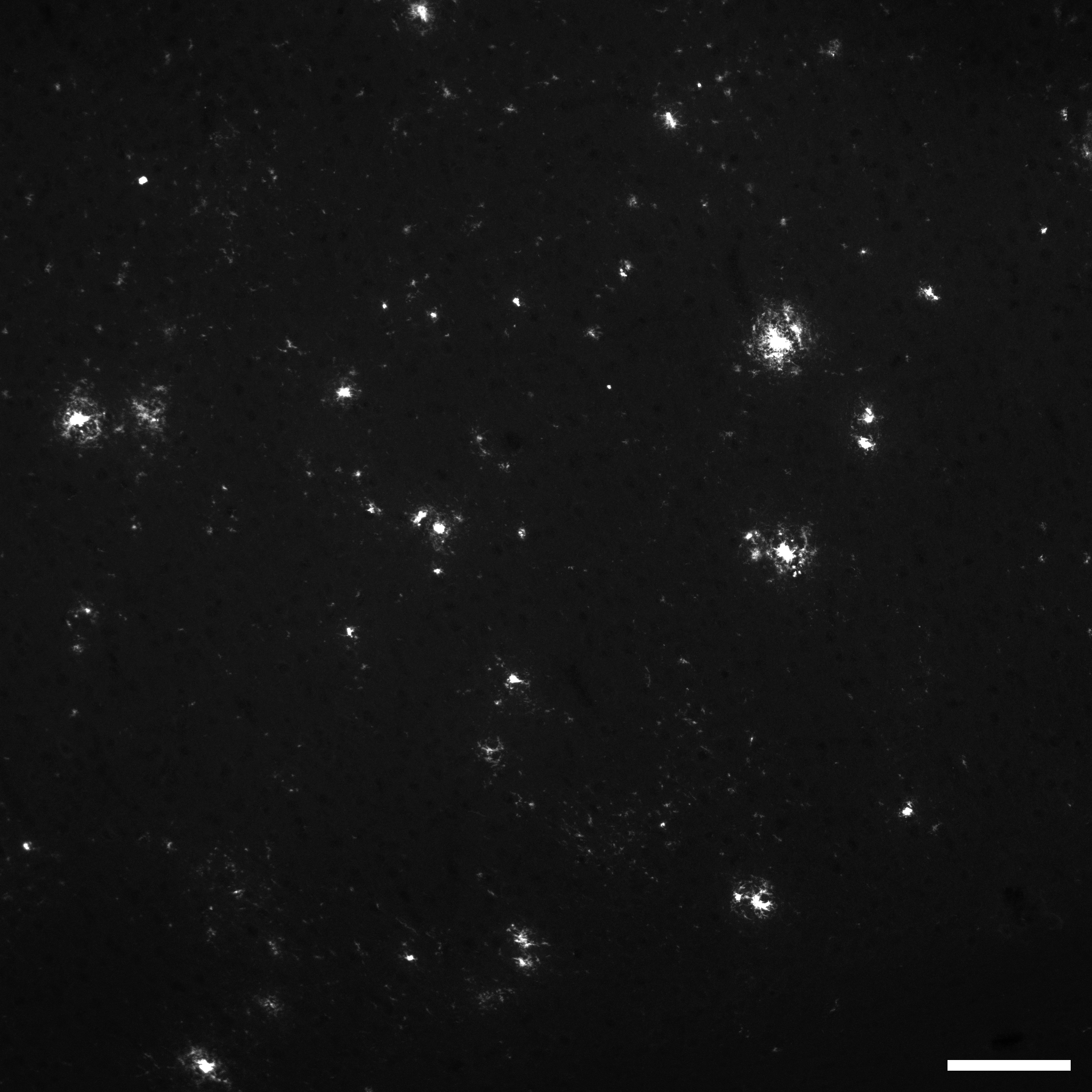

Supplement: Supplementary file 9 — Source data Fig. 1 [file 44321_2024_162_MOESM9_ESM.zip › Figure 1/1B/1b.APOE2_x-34.tif]

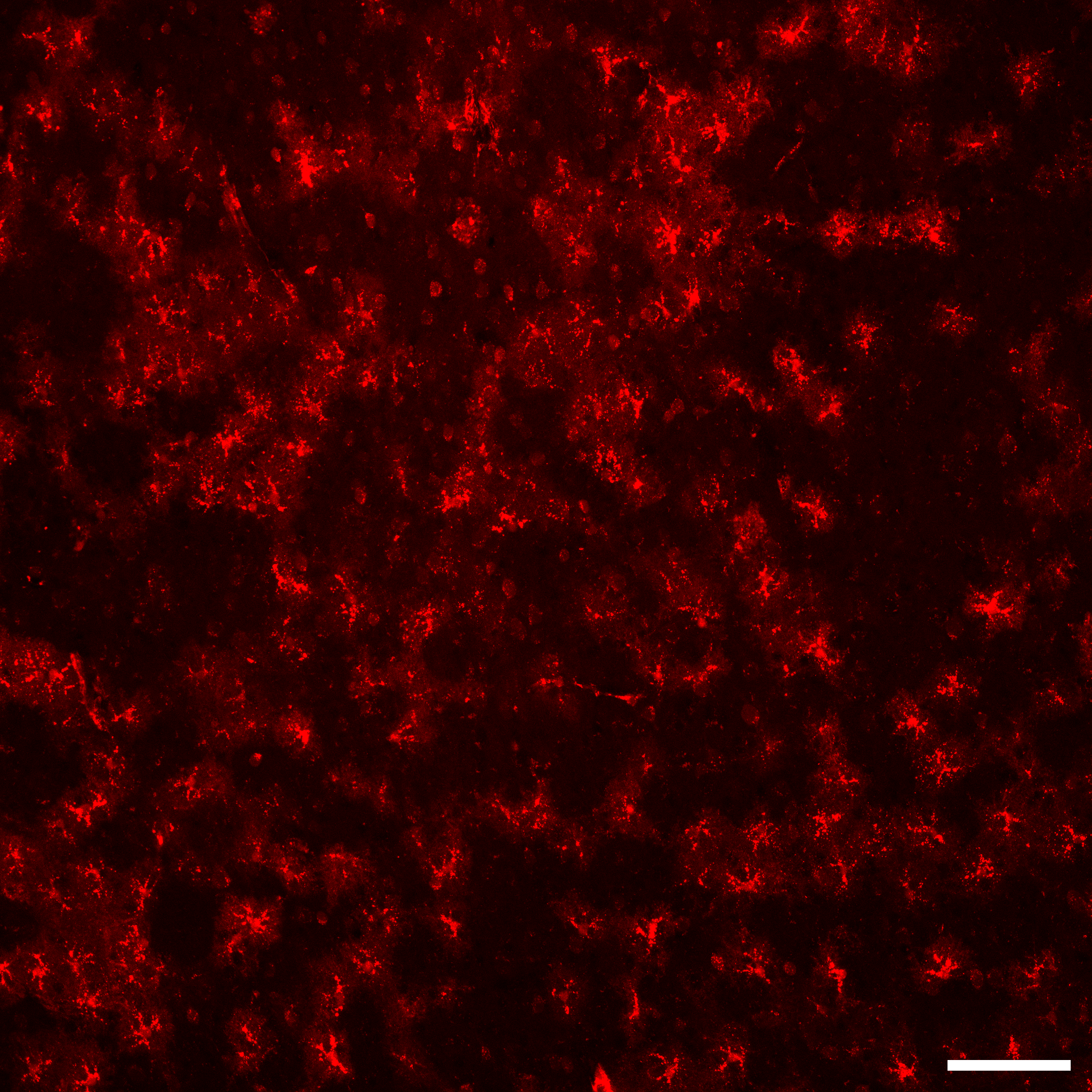

Supplement: Supplementary file 9 — Source data Fig. 1 [file 44321_2024_162_MOESM9_ESM.zip › Figure 1/1B/1B.APOE3_ mcherry.tif]

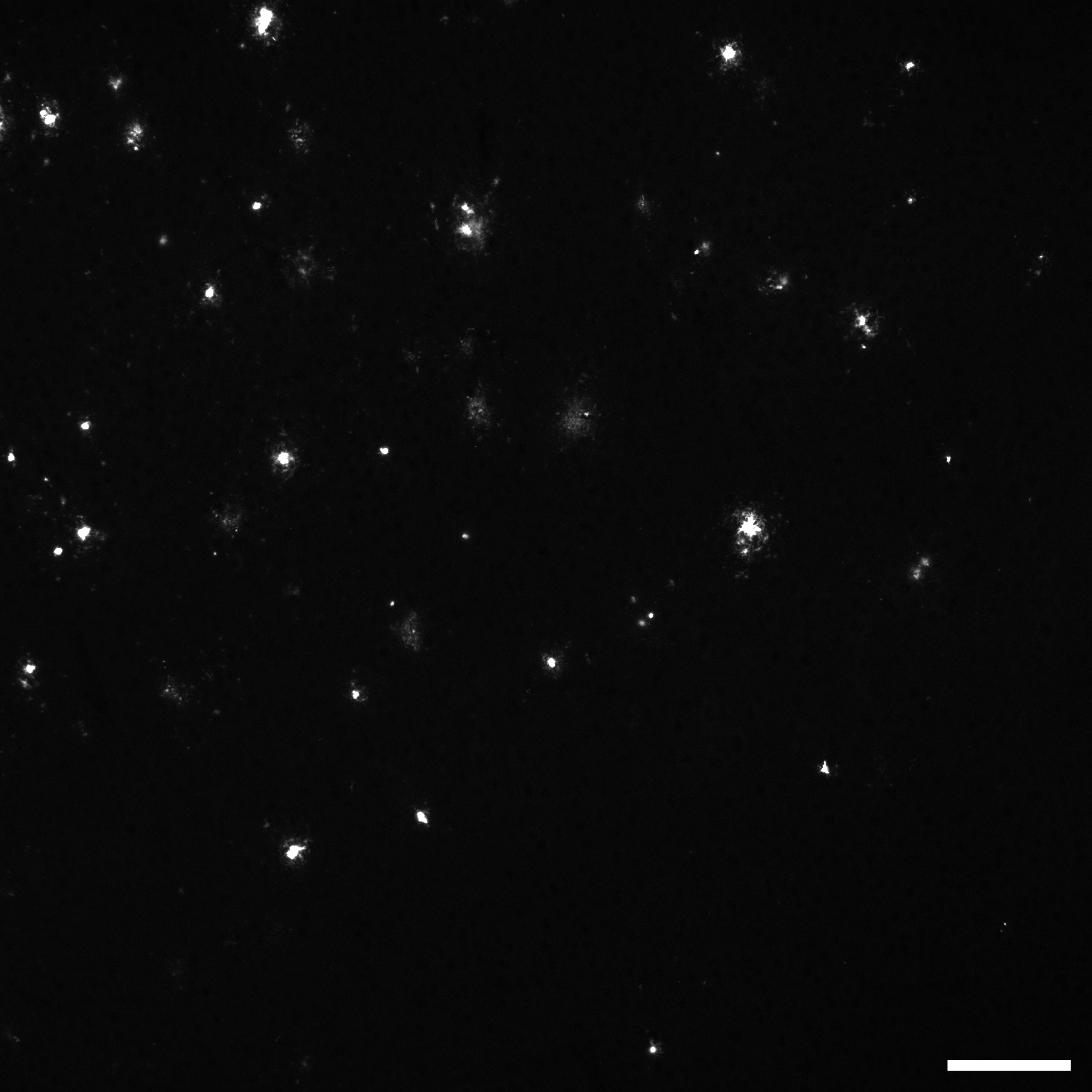

Supplement: Supplementary file 9 — Source data Fig. 1 [file 44321_2024_162_MOESM9_ESM.zip › Figure 1/1B/1B.APOE3_x-34.tif]

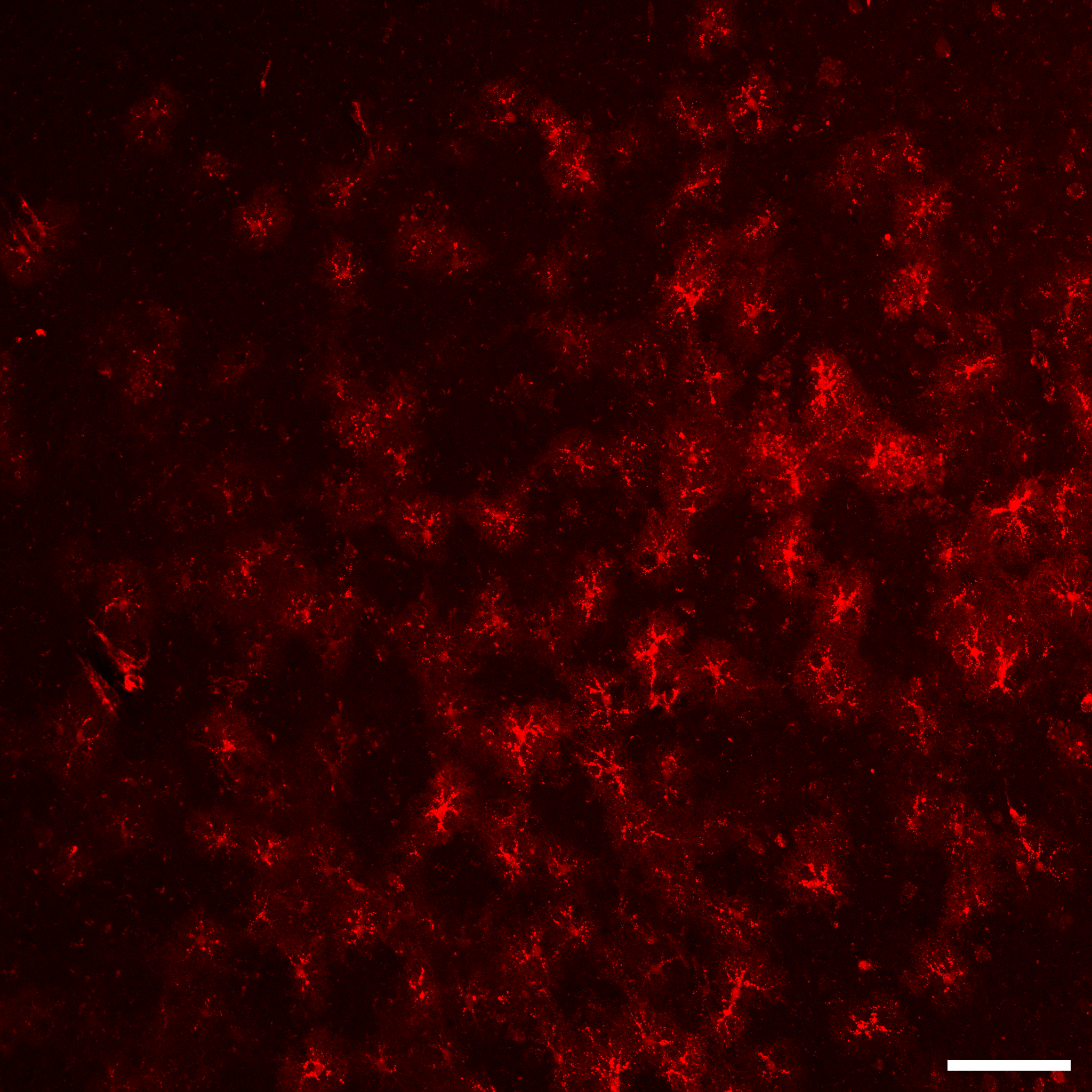

Supplement: Supplementary file 9 — Source data Fig. 1 [file 44321_2024_162_MOESM9_ESM.zip › Figure 1/1B/1B.APOE4_mcherry.tif]

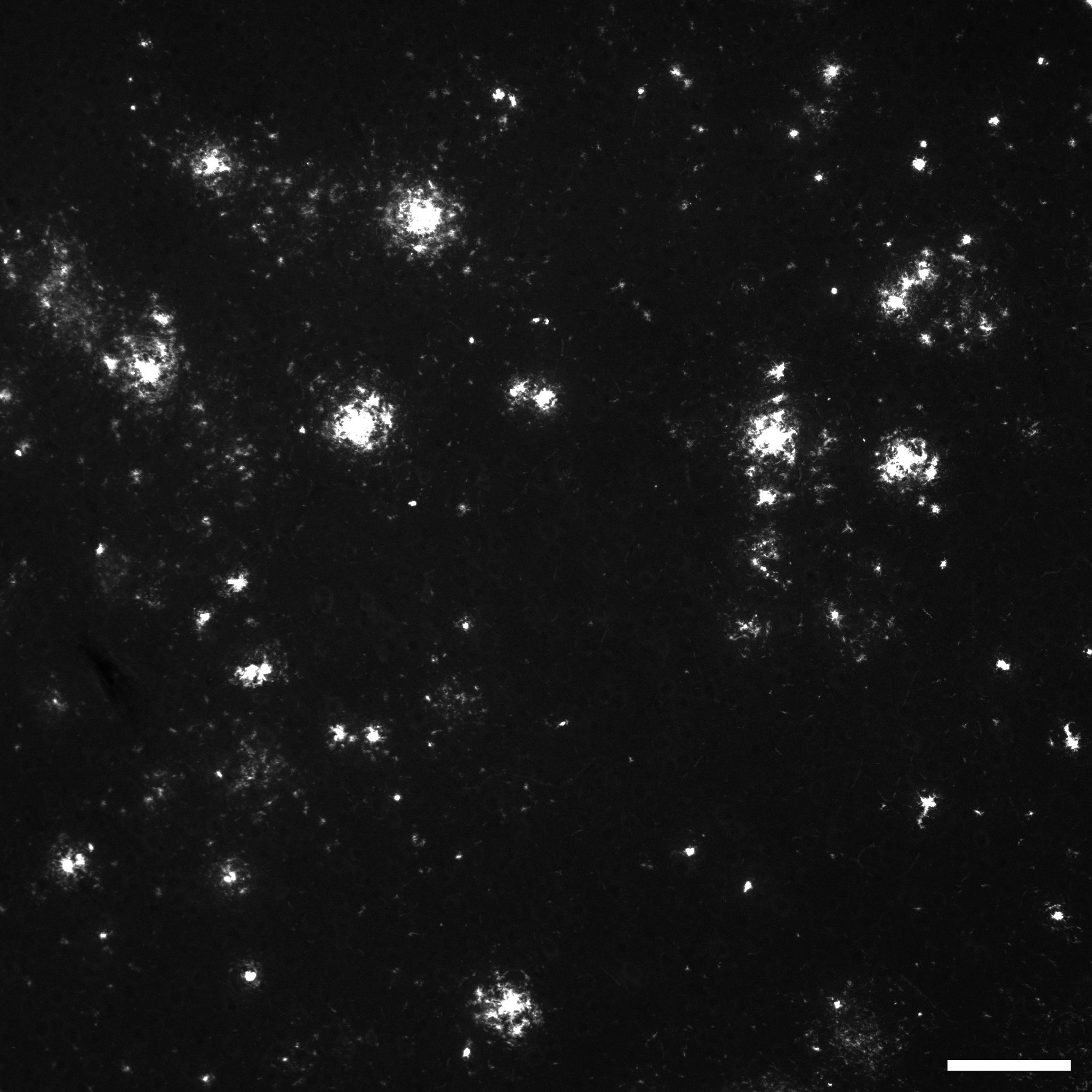

Supplement: Supplementary file 9 — Source data Fig. 1 [file 44321_2024_162_MOESM9_ESM.zip › Figure 1/1B/1B.APOE4_x-34.tif]

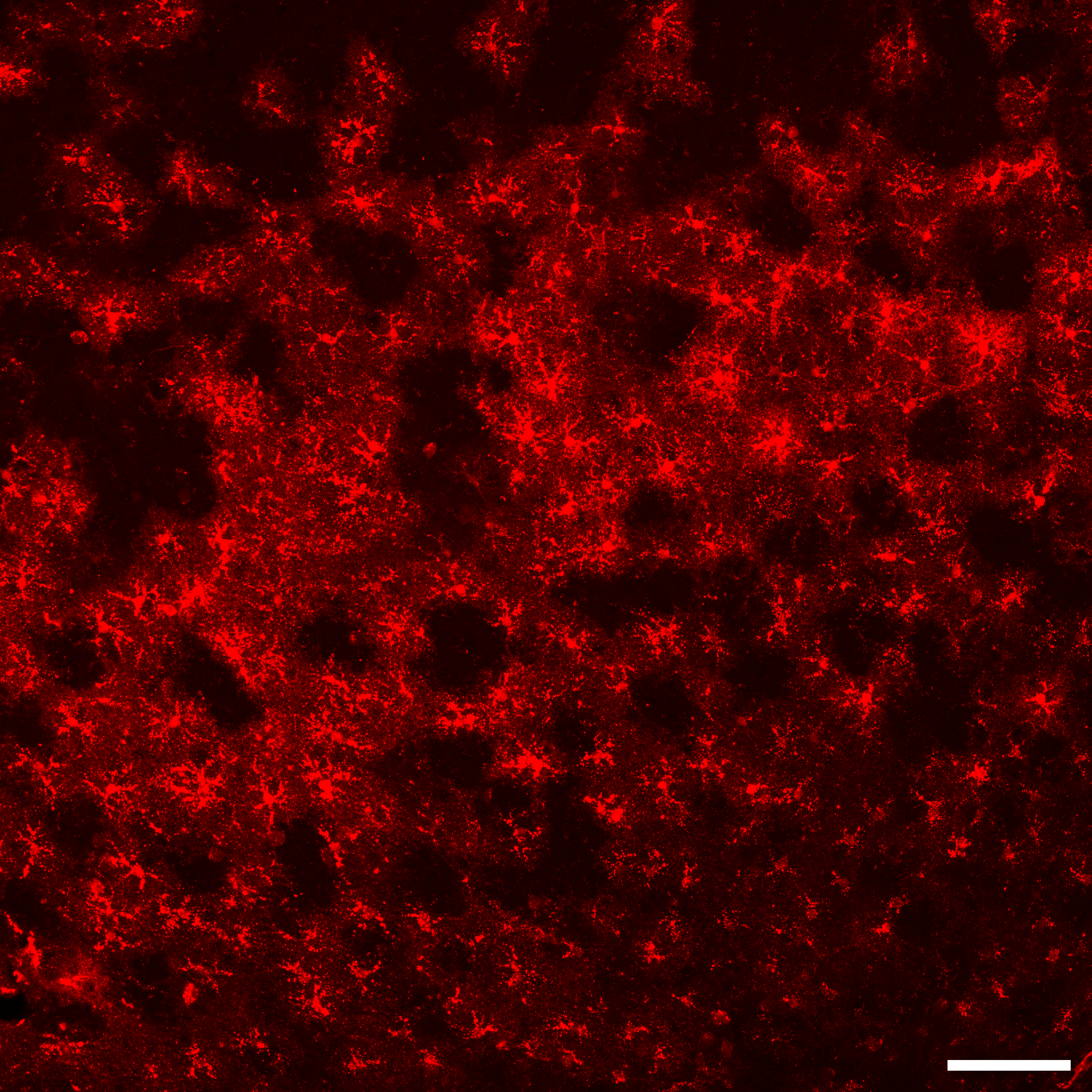

Supplement: Supplementary file 9 — Source data Fig. 1 [file 44321_2024_162_MOESM9_ESM.zip › Figure 1/1B/1B.APOEKO_mcherry.tif]

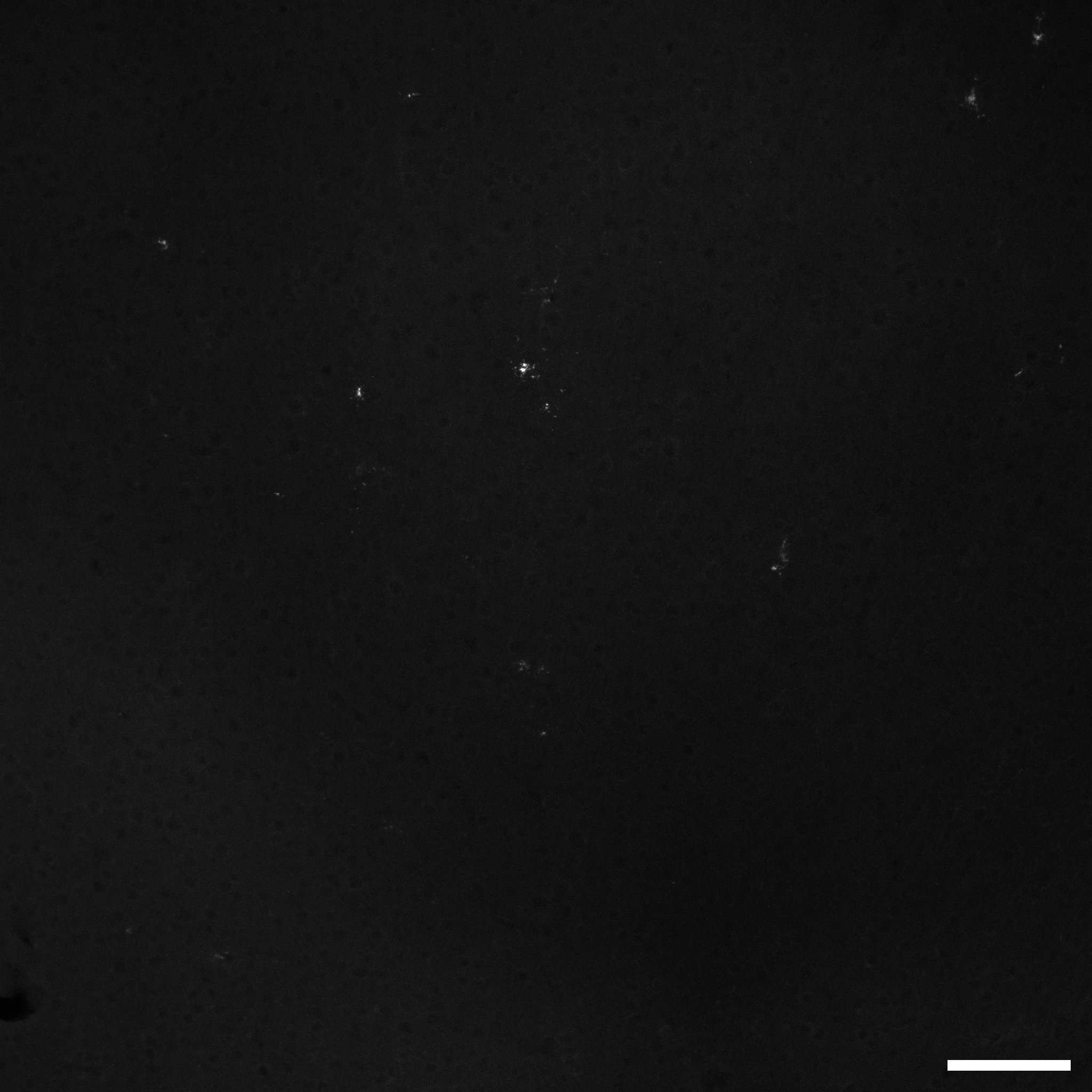

Supplement: Supplementary file 9 — Source data Fig. 1 [file 44321_2024_162_MOESM9_ESM.zip › Figure 1/1B/1B.APOEKO_x-34.tif]

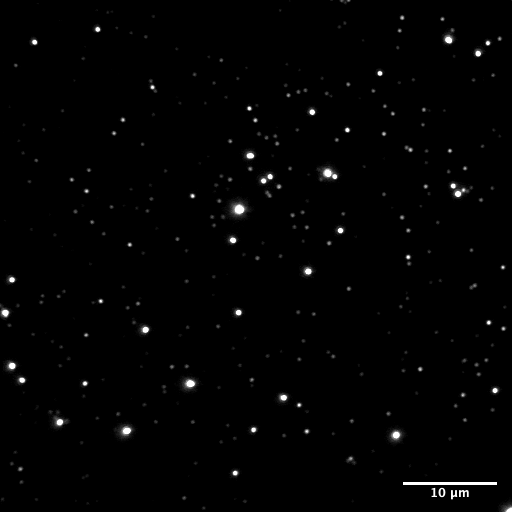

Supplement: Supplementary file 9 — Source data Fig. 1 [file 44321_2024_162_MOESM9_ESM.zip › Figure 1/1F/1f.APOE2.tif]

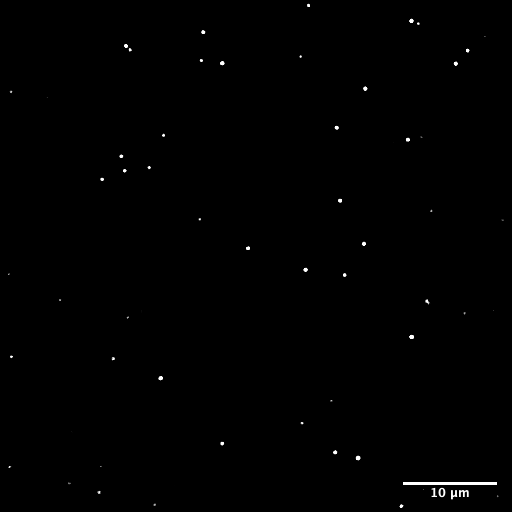

Supplement: Supplementary file 9 — Source data Fig. 1 [file 44321_2024_162_MOESM9_ESM.zip › Figure 1/1F/1f.APOE3.tif]

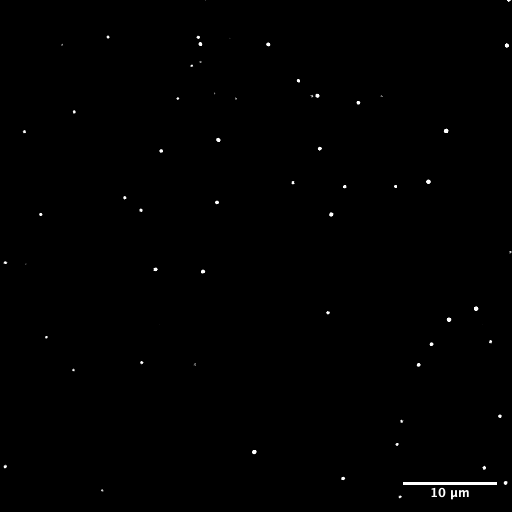

Supplement: Supplementary file 9 — Source data Fig. 1 [file 44321_2024_162_MOESM9_ESM.zip › Figure 1/1F/1f.APOE4.tif]

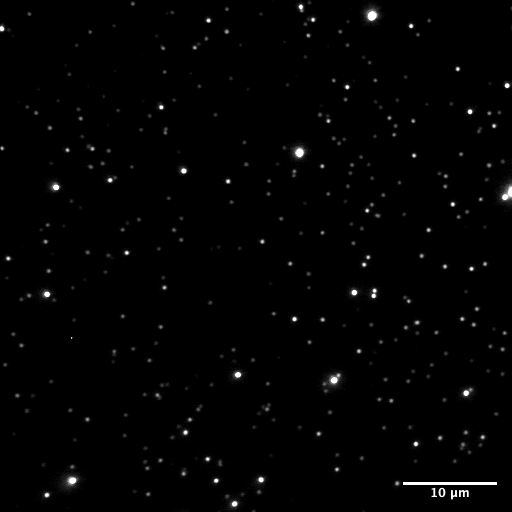

Supplement: Supplementary file 9 — Source data Fig. 1 [file 44321_2024_162_MOESM9_ESM.zip › Figure 1/1F/1f.APOEKO.tif]

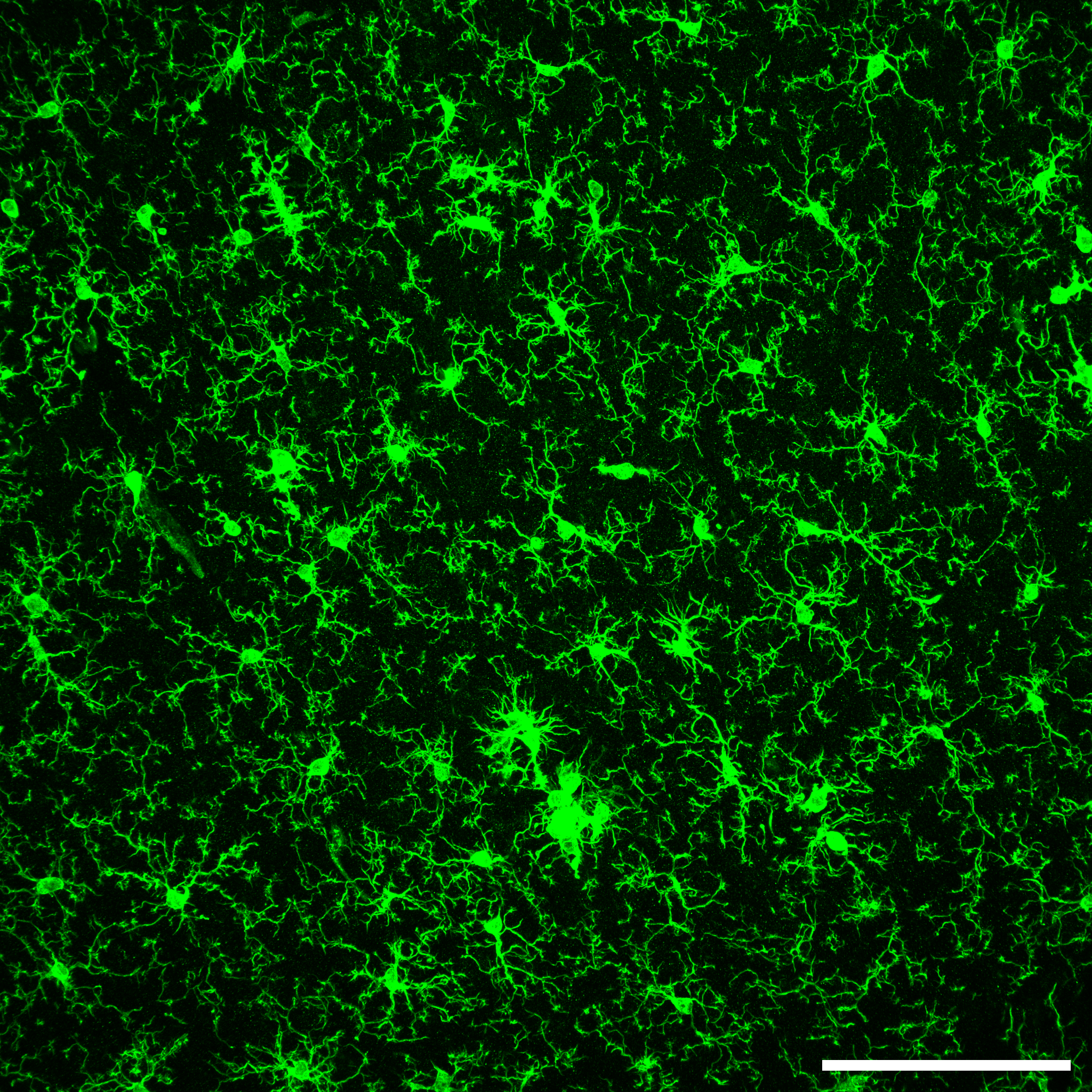

Supplement: Supplementary file 11 — Source data Fig. 4 [file 44321_2024_162_MOESM11_ESM.zip › Figure 4/4A/4A.APOE2_iba1.tif]

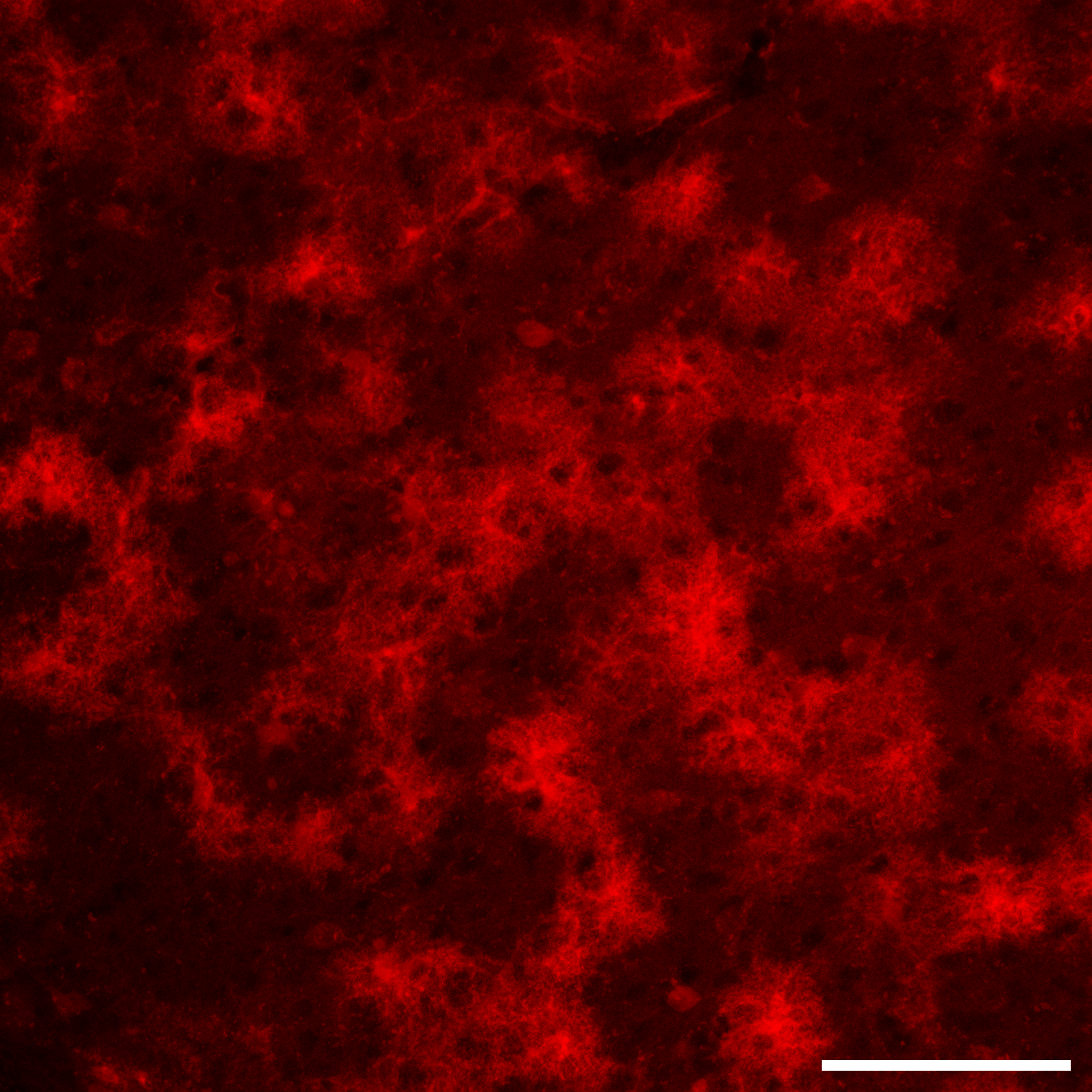

Supplement: Supplementary file 11 — Source data Fig. 4 [file 44321_2024_162_MOESM11_ESM.zip › Figure 4/4A/4A.APOE2_mcherry.tif]

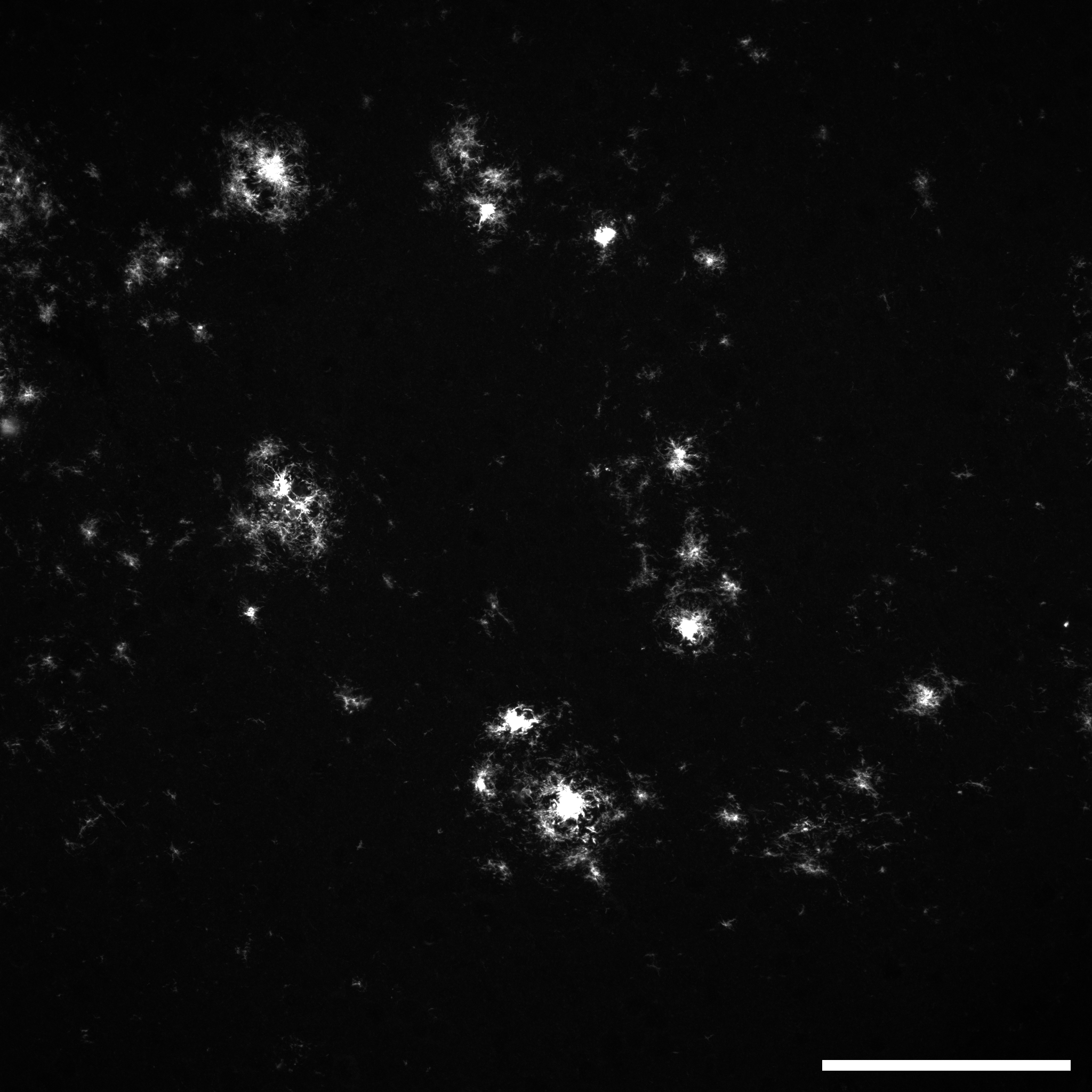

Supplement: Supplementary file 11 — Source data Fig. 4 [file 44321_2024_162_MOESM11_ESM.zip › Figure 4/4A/4A.APOE2_x-34.tif]

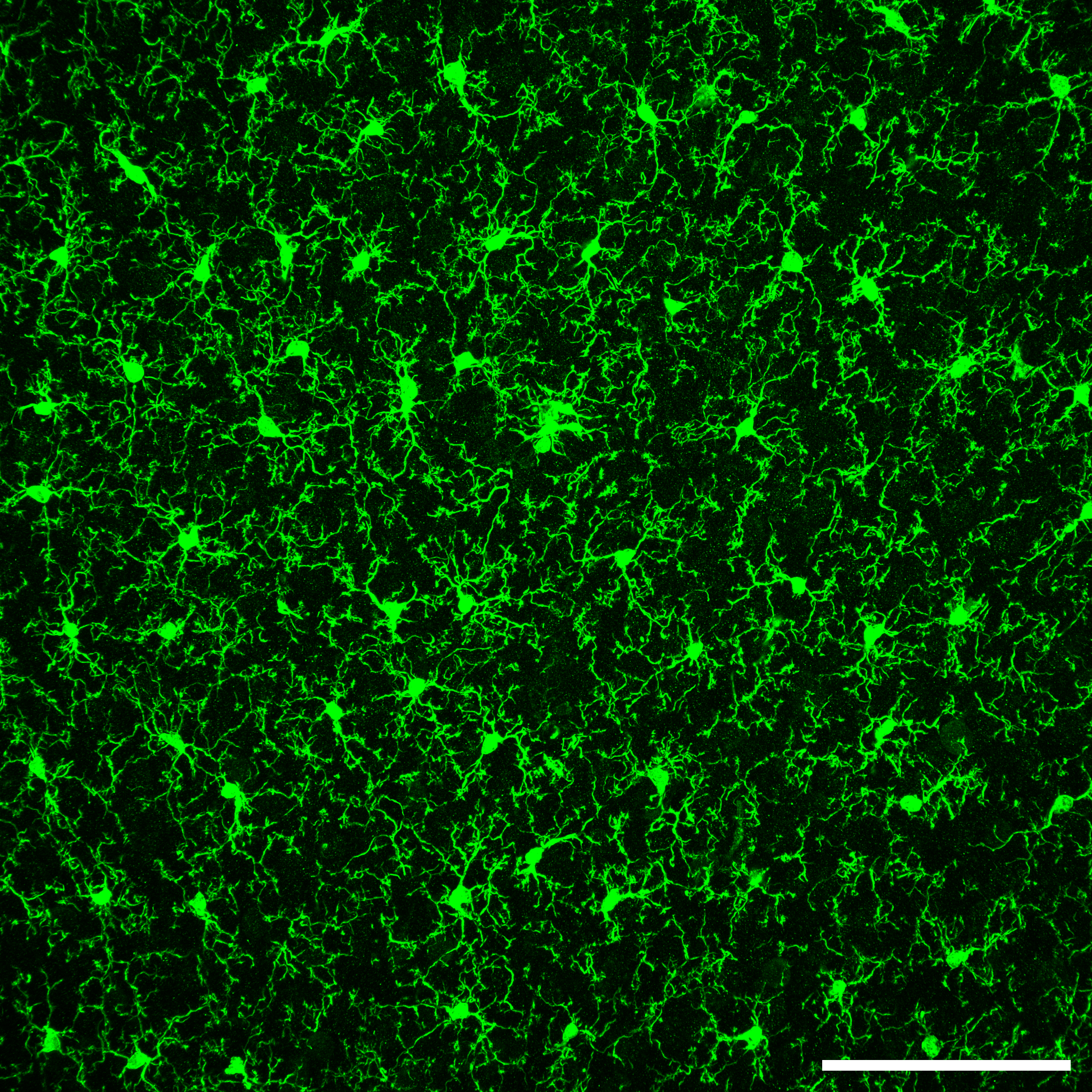

Supplement: Supplementary file 11 — Source data Fig. 4 [file 44321_2024_162_MOESM11_ESM.zip › Figure 4/4A/4A.APOE3_iba1.tif]

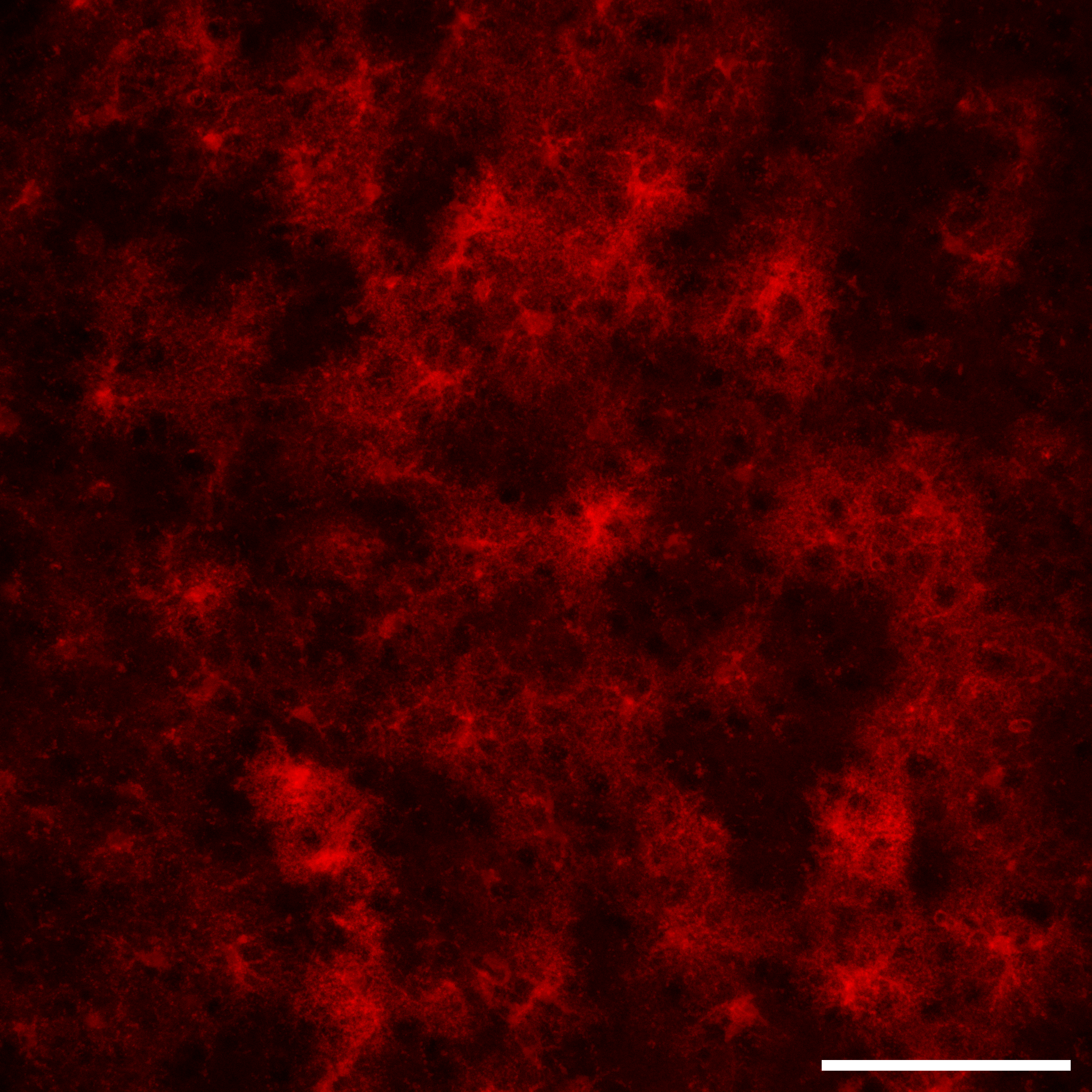

Supplement: Supplementary file 11 — Source data Fig. 4 [file 44321_2024_162_MOESM11_ESM.zip › Figure 4/4A/4A.APOE3_mcherry.tif]

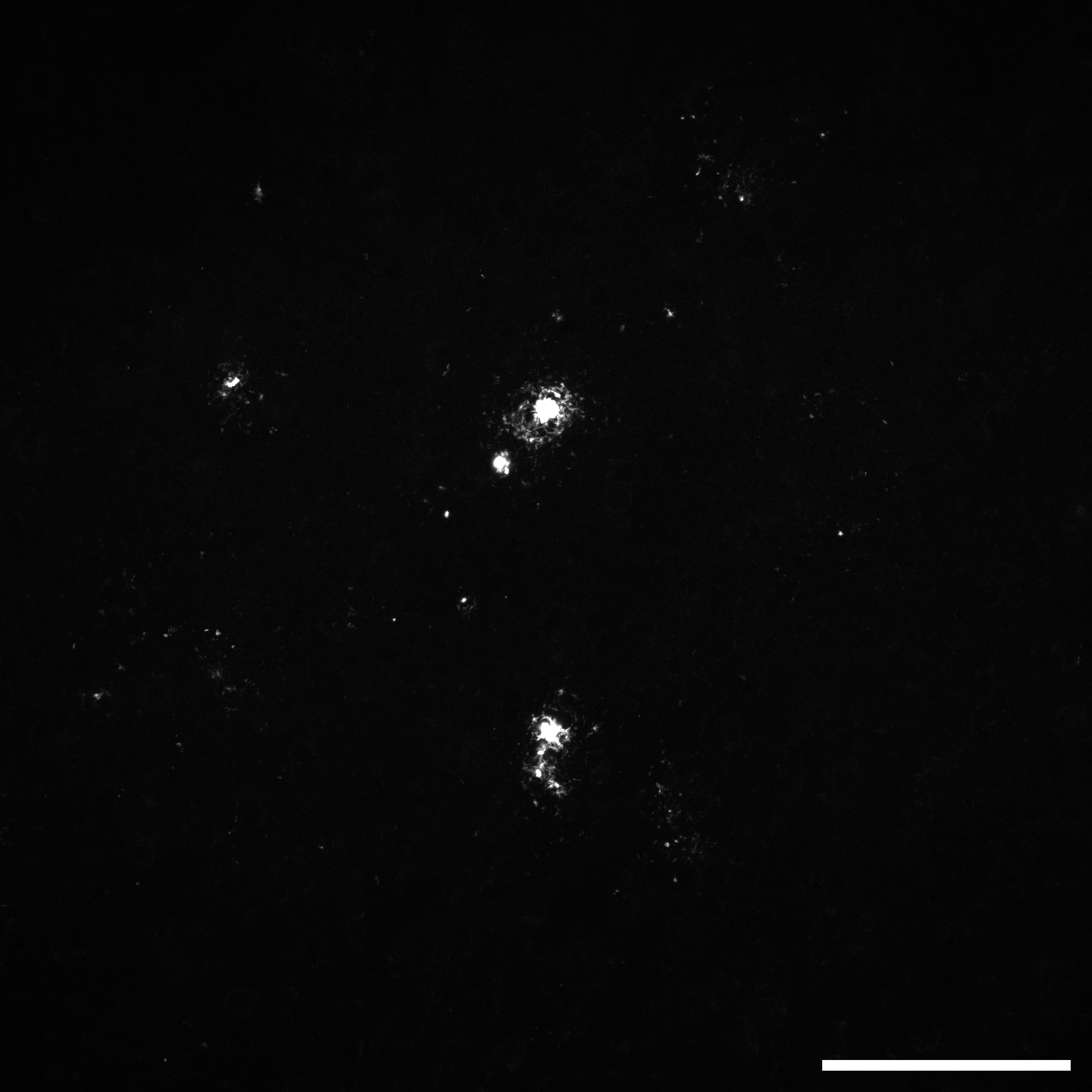

Supplement: Supplementary file 11 — Source data Fig. 4 [file 44321_2024_162_MOESM11_ESM.zip › Figure 4/4A/4A.APOE3_x-34.tif]

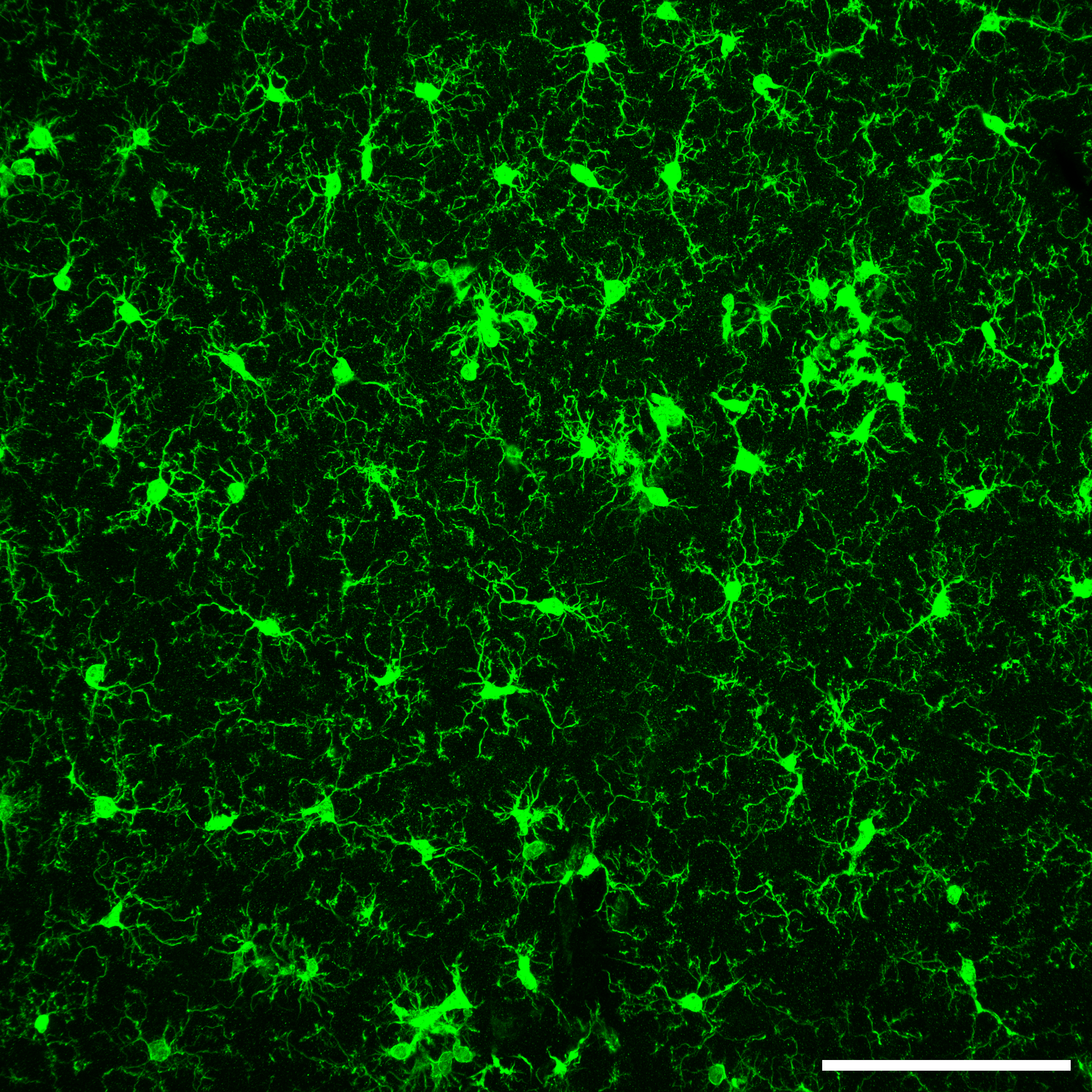

Supplement: Supplementary file 11 — Source data Fig. 4 [file 44321_2024_162_MOESM11_ESM.zip › Figure 4/4A/4A.APOE4_iba1.tif]

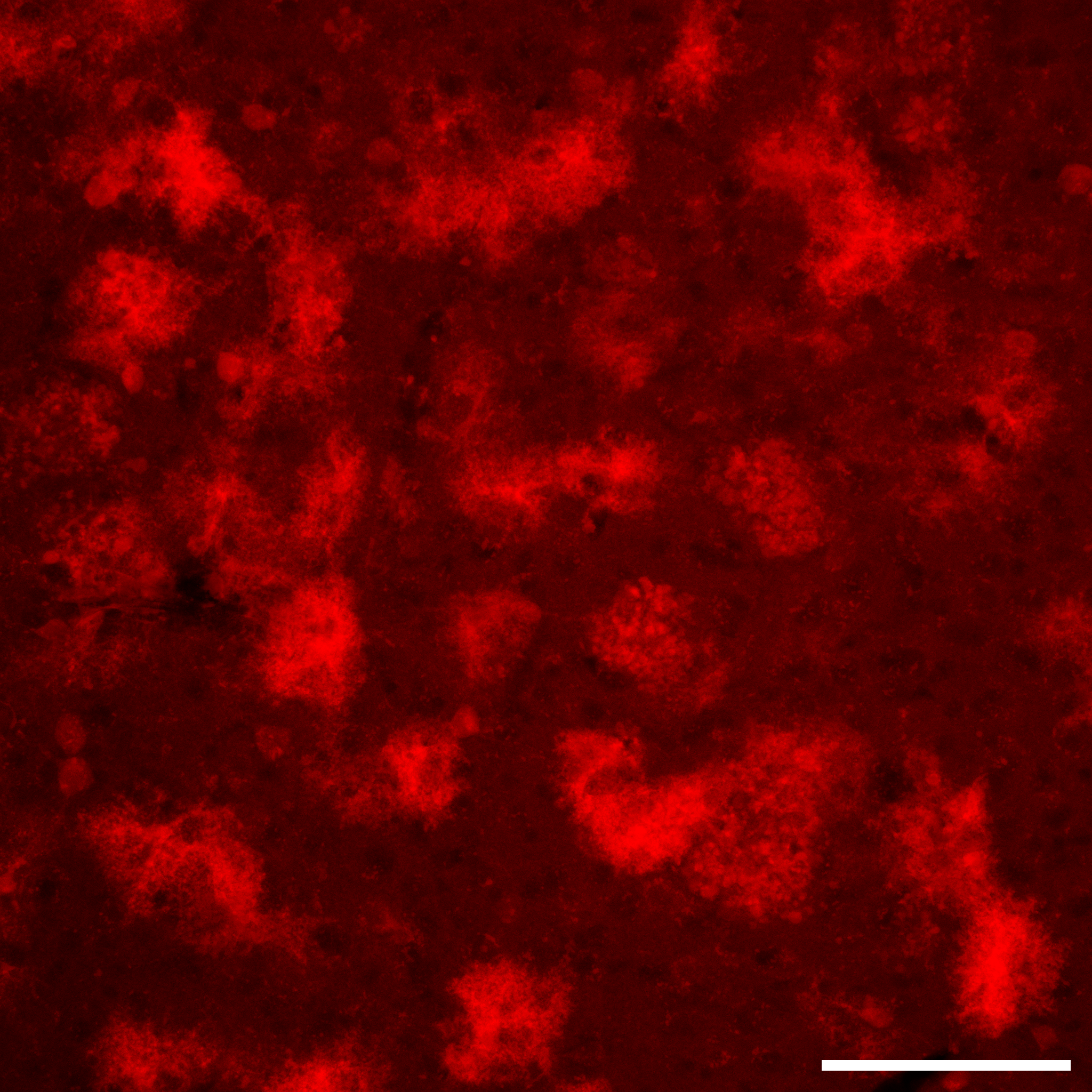

Supplement: Supplementary file 11 — Source data Fig. 4 [file 44321_2024_162_MOESM11_ESM.zip › Figure 4/4A/4A.APOE4_mcherry.tif]

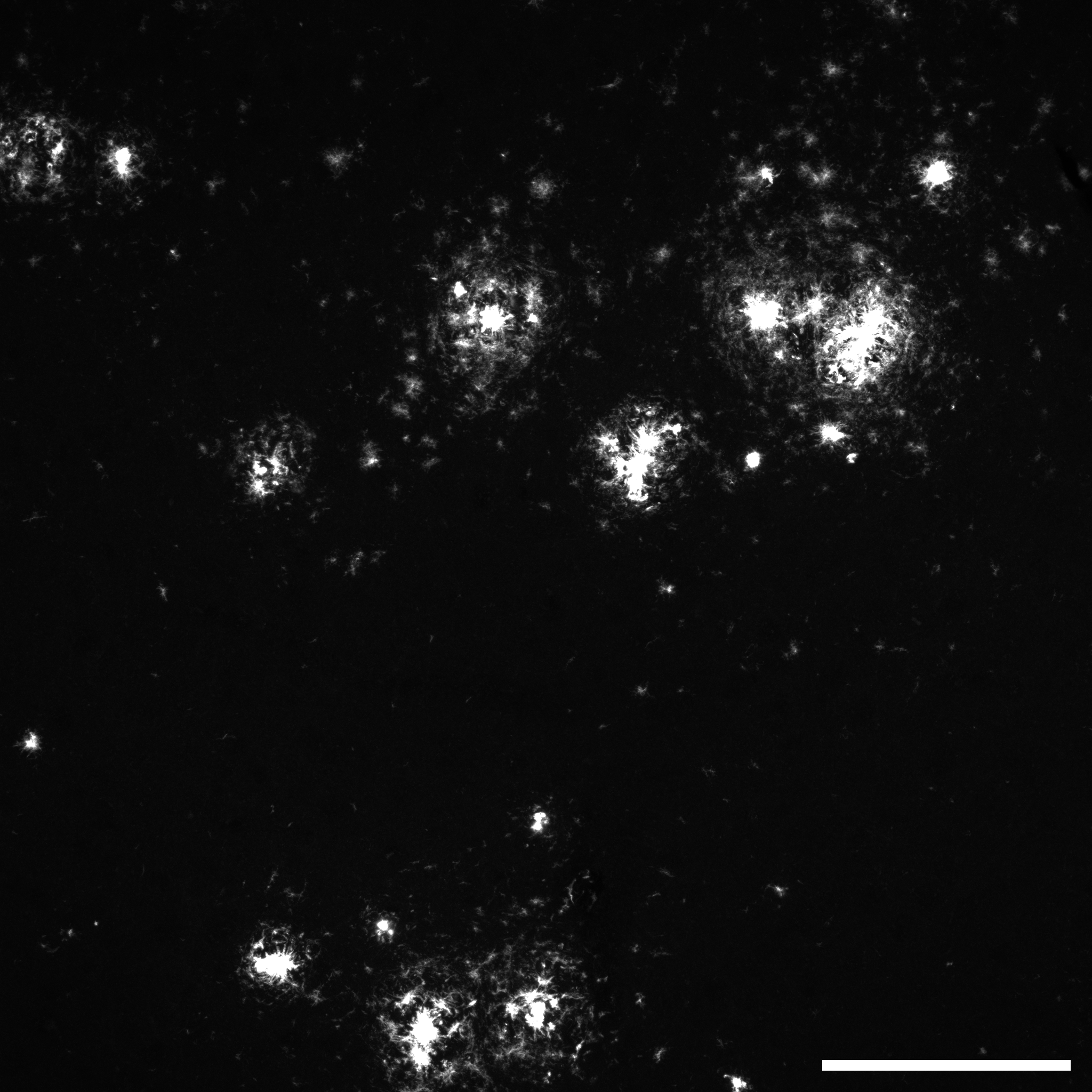

Supplement: Supplementary file 11 — Source data Fig. 4 [file 44321_2024_162_MOESM11_ESM.zip › Figure 4/4A/4A.APOE4_x-34.tif]

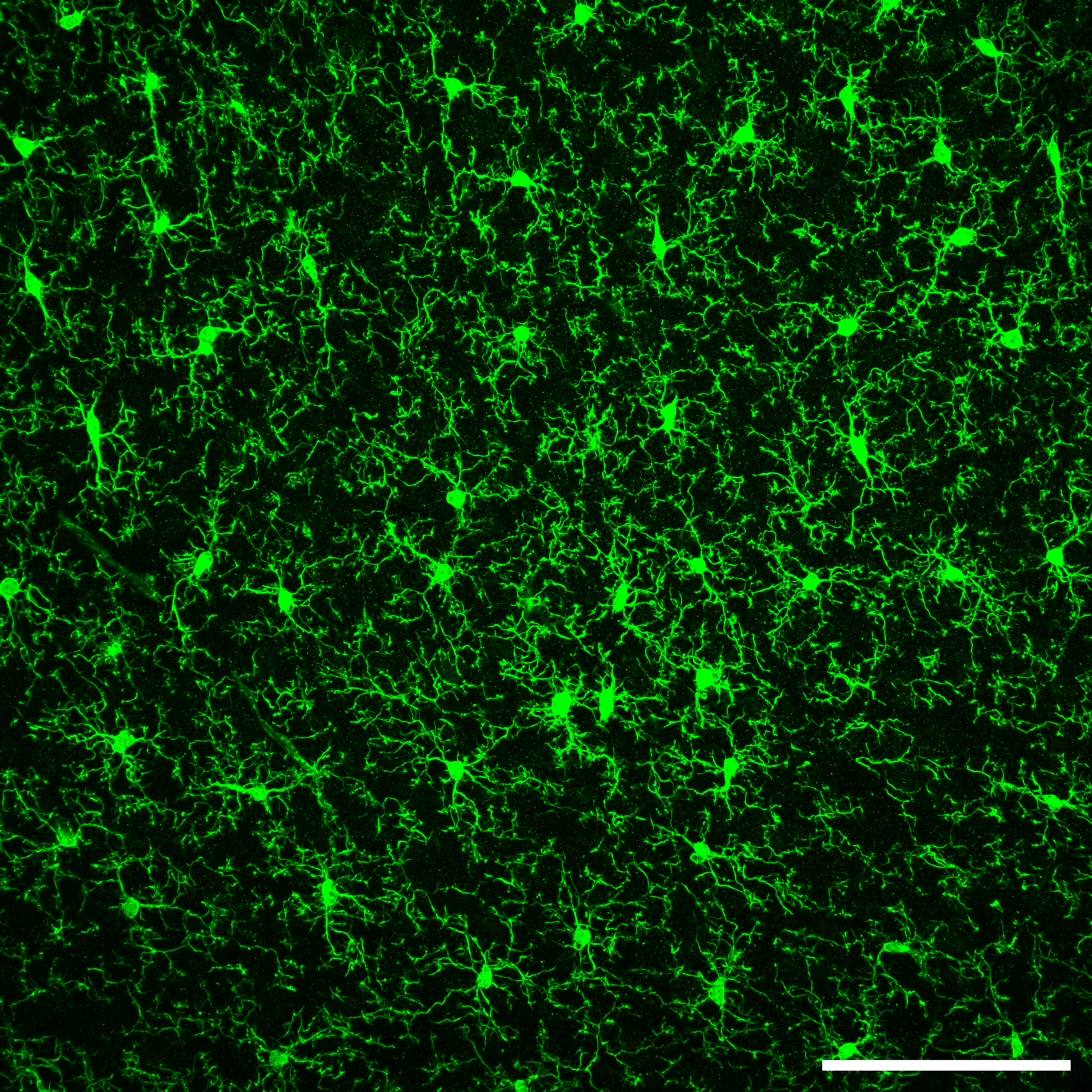

Supplement: Supplementary file 11 — Source data Fig. 4 [file 44321_2024_162_MOESM11_ESM.zip › Figure 4/4A/4A.APOEKO_iba1.tif]

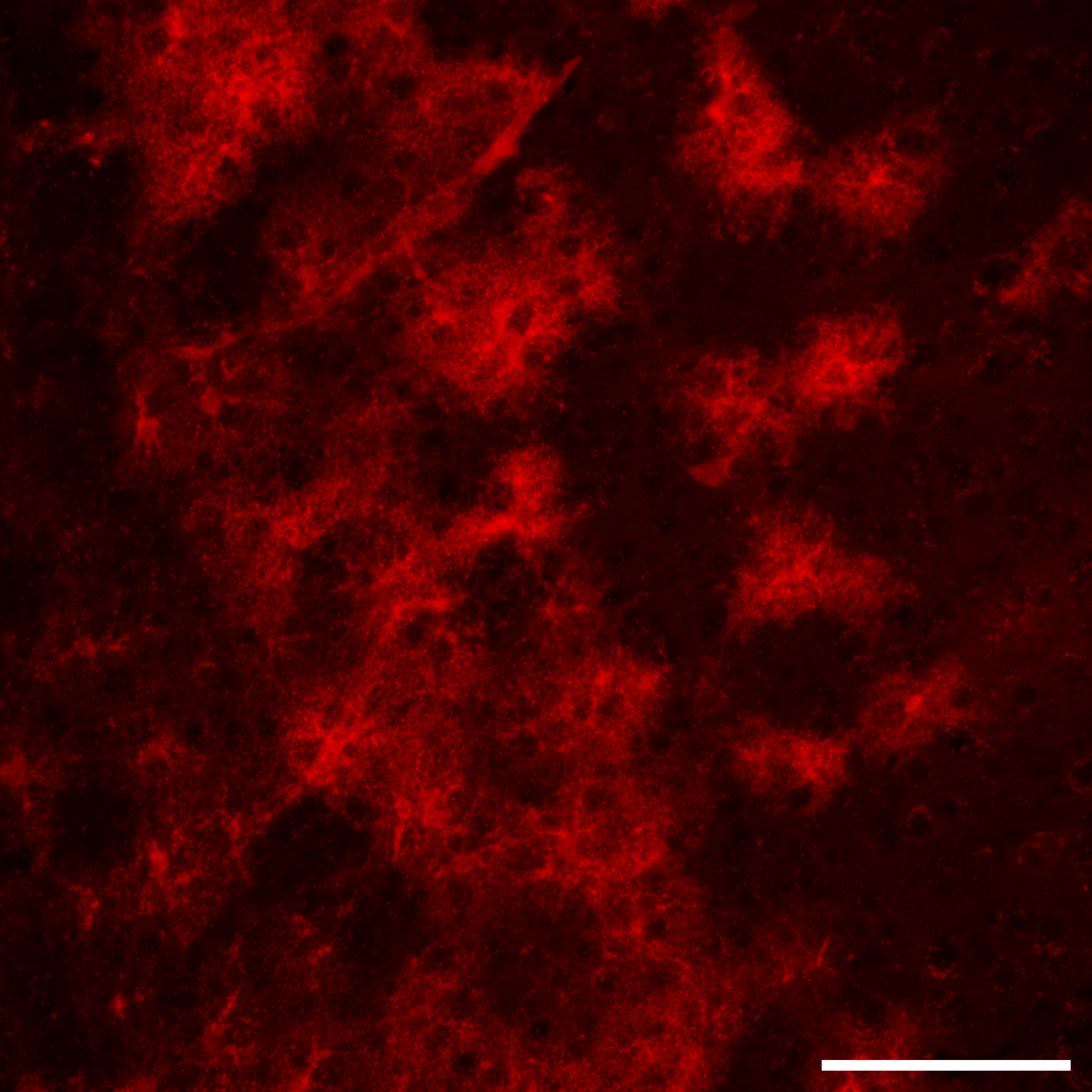

Supplement: Supplementary file 11 — Source data Fig. 4 [file 44321_2024_162_MOESM11_ESM.zip › Figure 4/4A/4A.APOEKO_mcherry.tif]

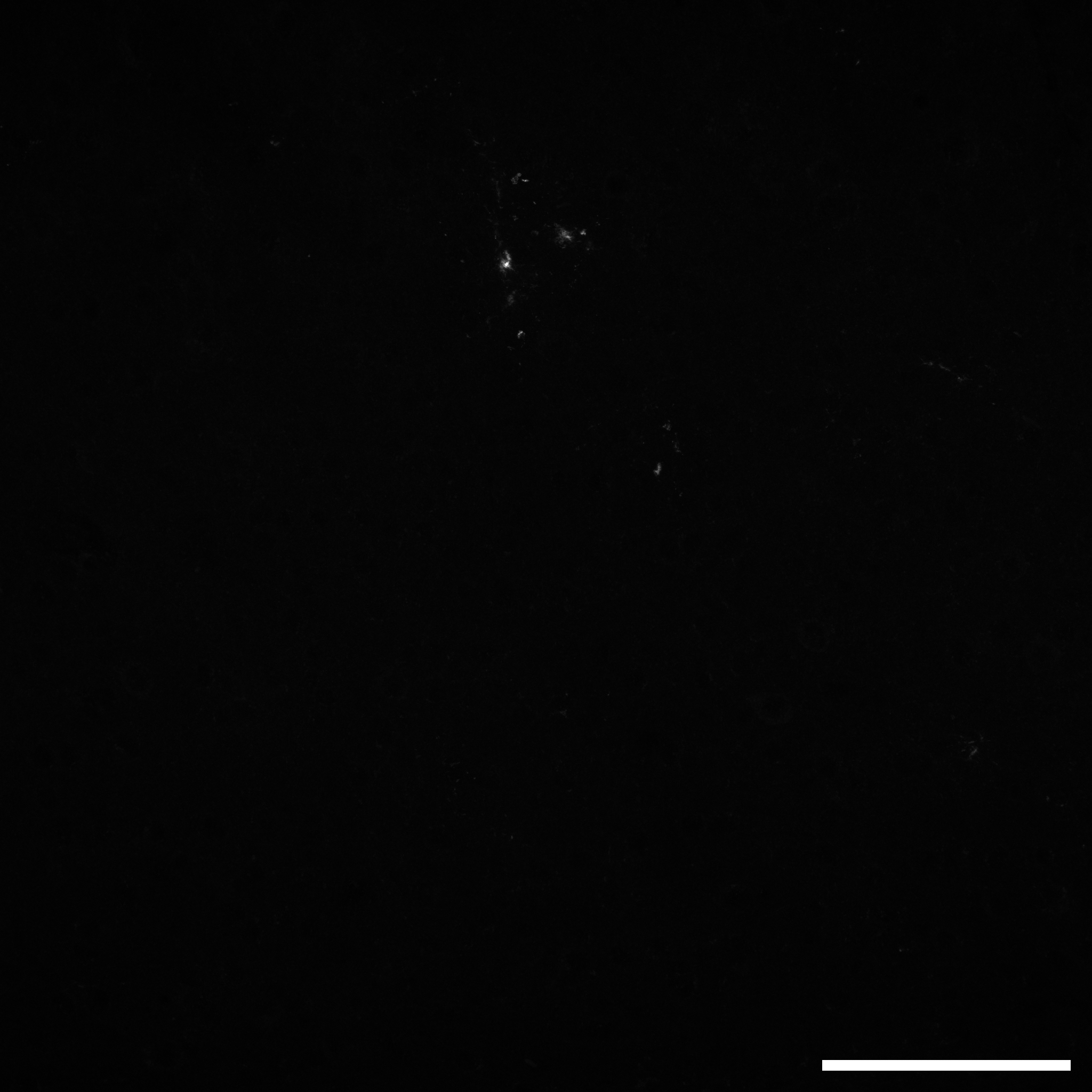

Supplement: Supplementary file 11 — Source data Fig. 4 [file 44321_2024_162_MOESM11_ESM.zip › Figure 4/4A/4A.APOEKO_x-34.tif]

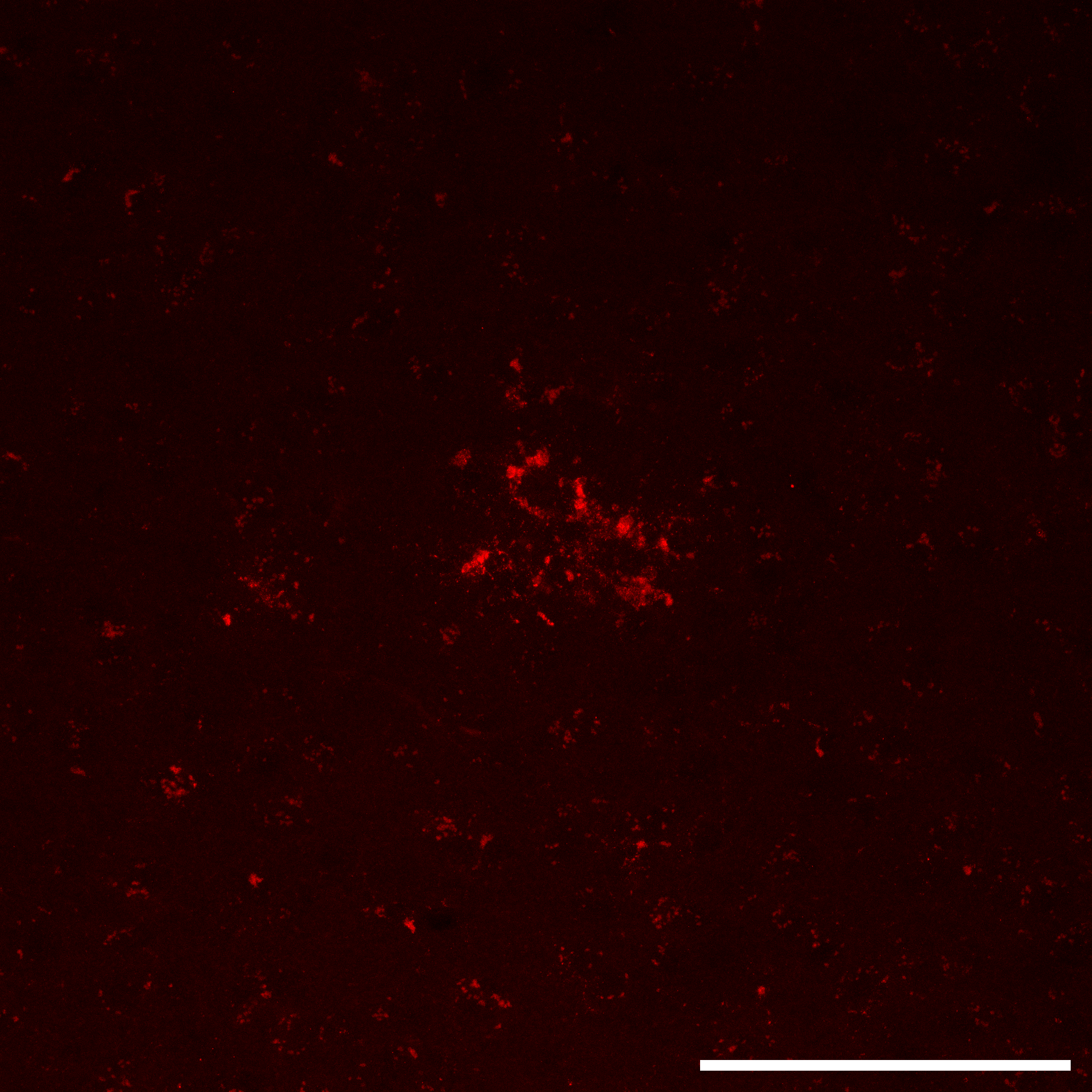

Supplement: Supplementary file 11 — Source data Fig. 4 [file 44321_2024_162_MOESM11_ESM.zip › Figure 4/4B/4B.APOE4_cd68.tif]

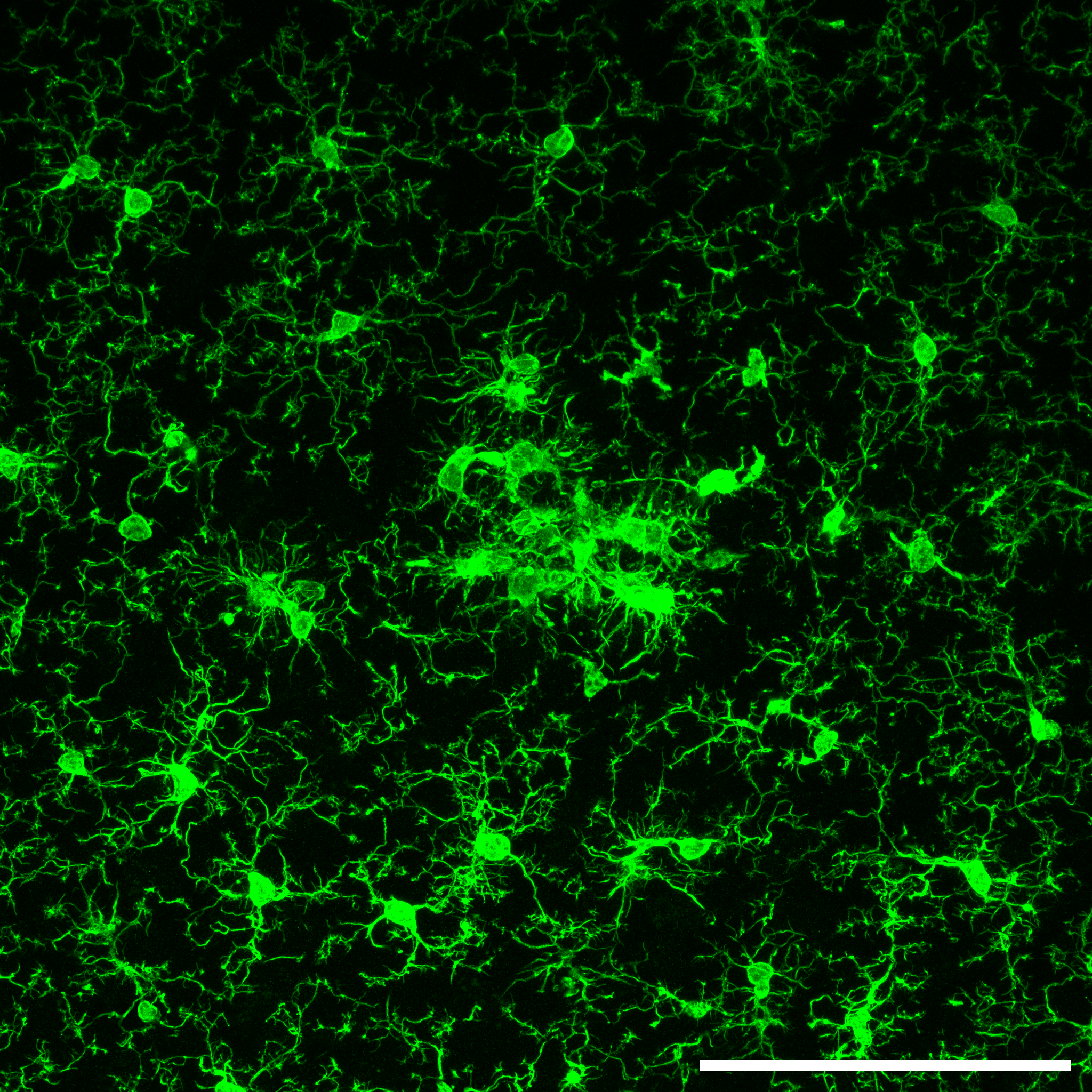

Supplement: Supplementary file 11 — Source data Fig. 4 [file 44321_2024_162_MOESM11_ESM.zip › Figure 4/4B/4B.APOE4_iba1.tif]

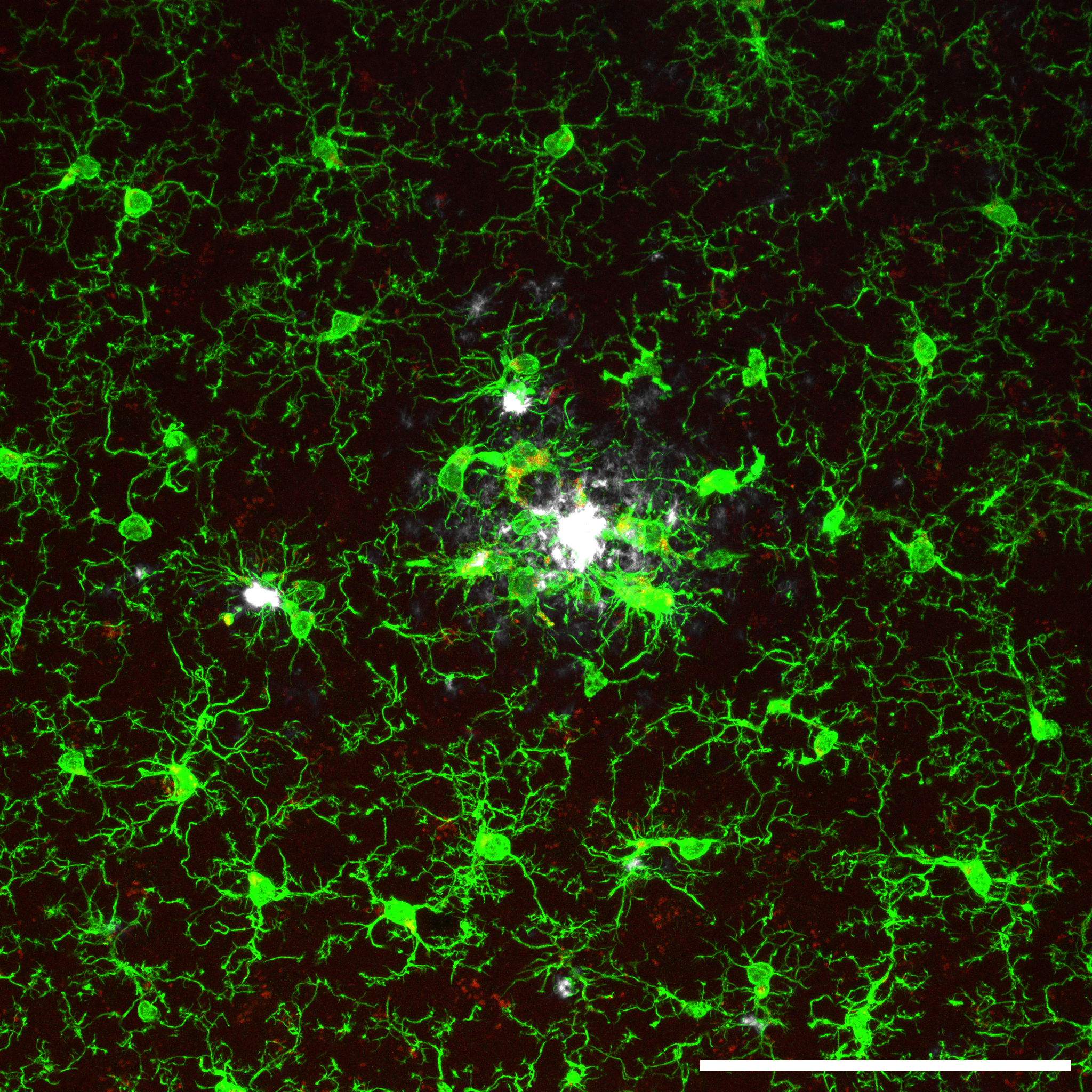

Supplement: Supplementary file 11 — Source data Fig. 4 [file 44321_2024_162_MOESM11_ESM.zip › Figure 4/4B/4B.APOE4_merge.tif]

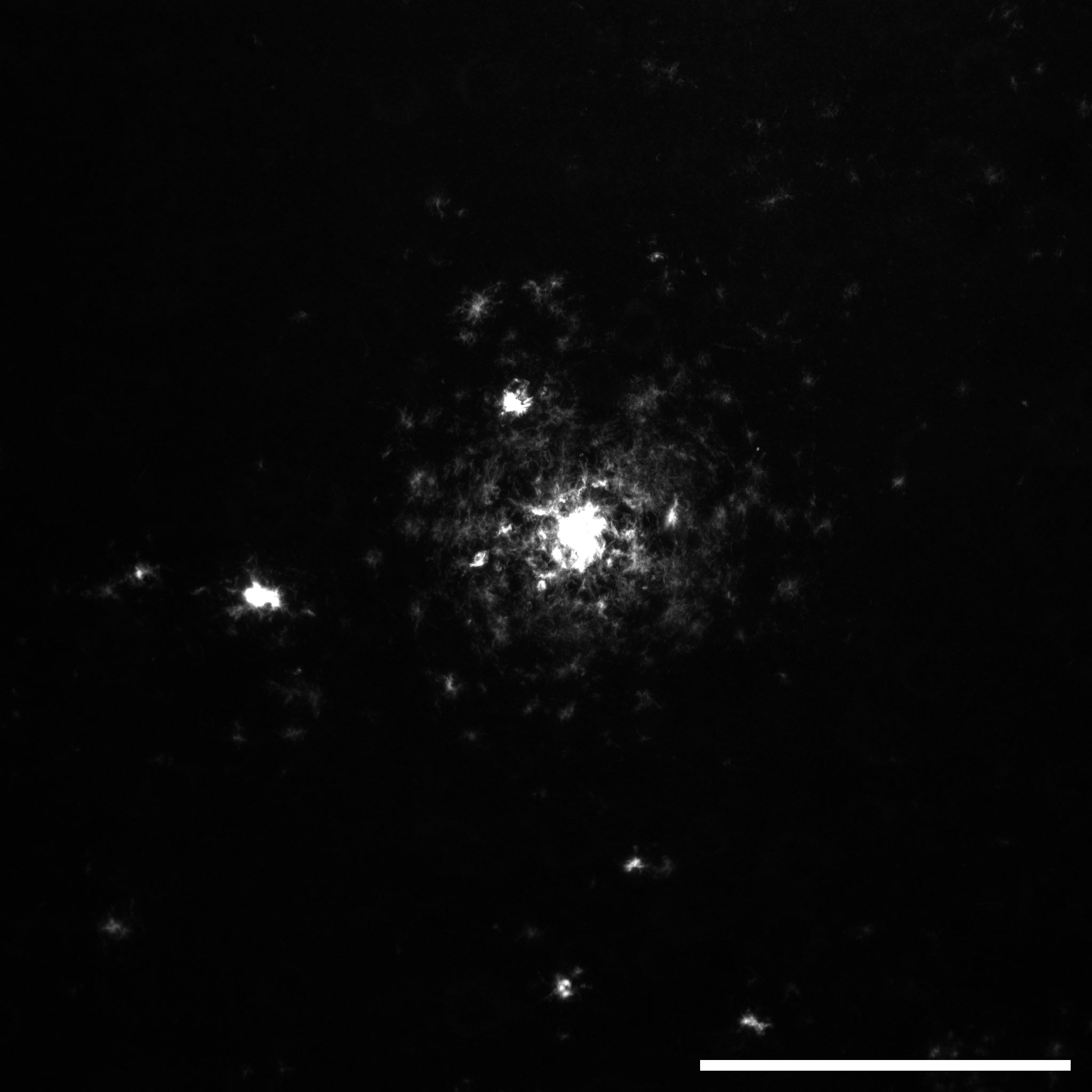

Supplement: Supplementary file 11 — Source data Fig. 4 [file 44321_2024_162_MOESM11_ESM.zip › Figure 4/4B/4B.APOE4_X34.tif]

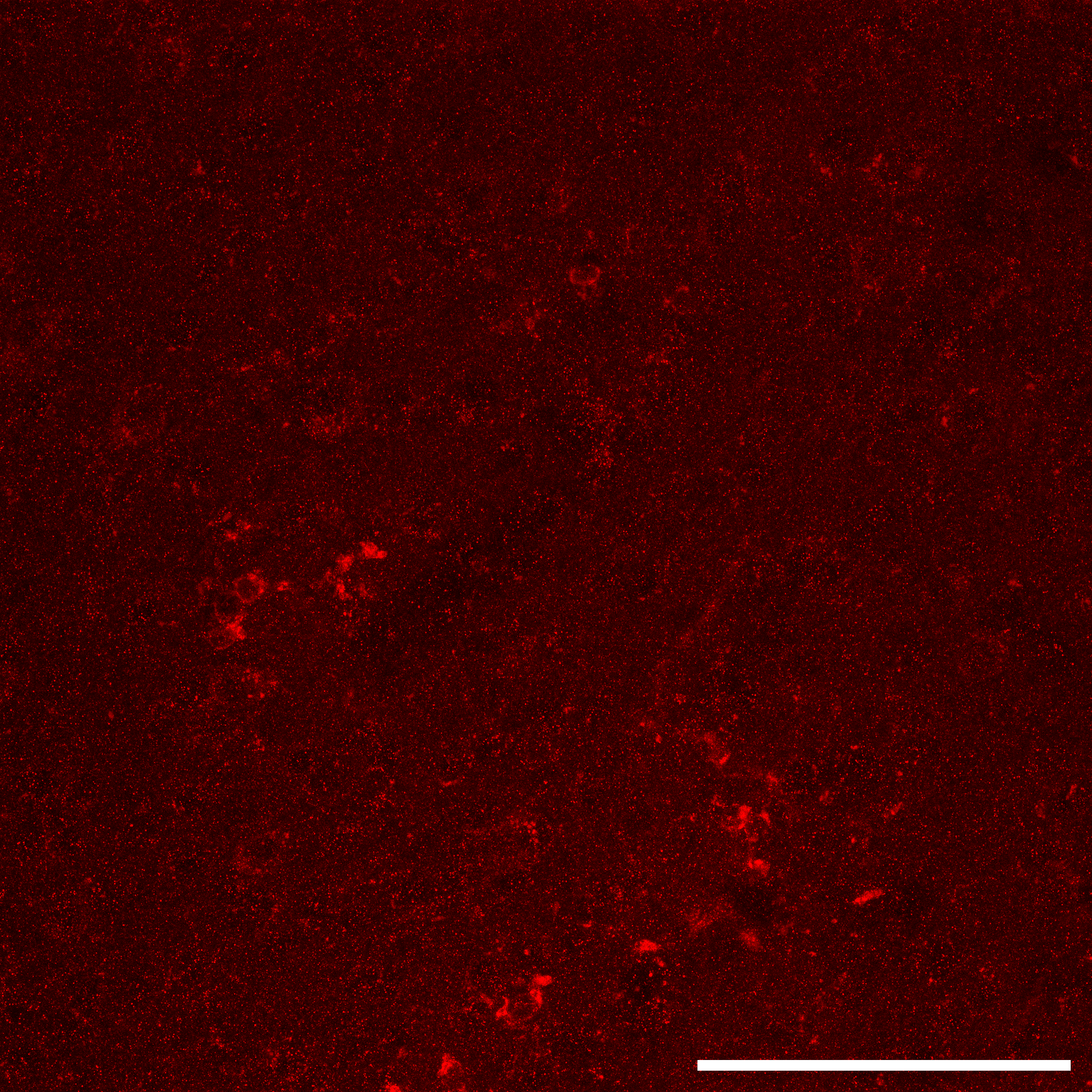

Supplement: Supplementary file 11 — Source data Fig. 4 [file 44321_2024_162_MOESM11_ESM.zip › Figure 4/4C/4C.APOE4_ctsd.tif]

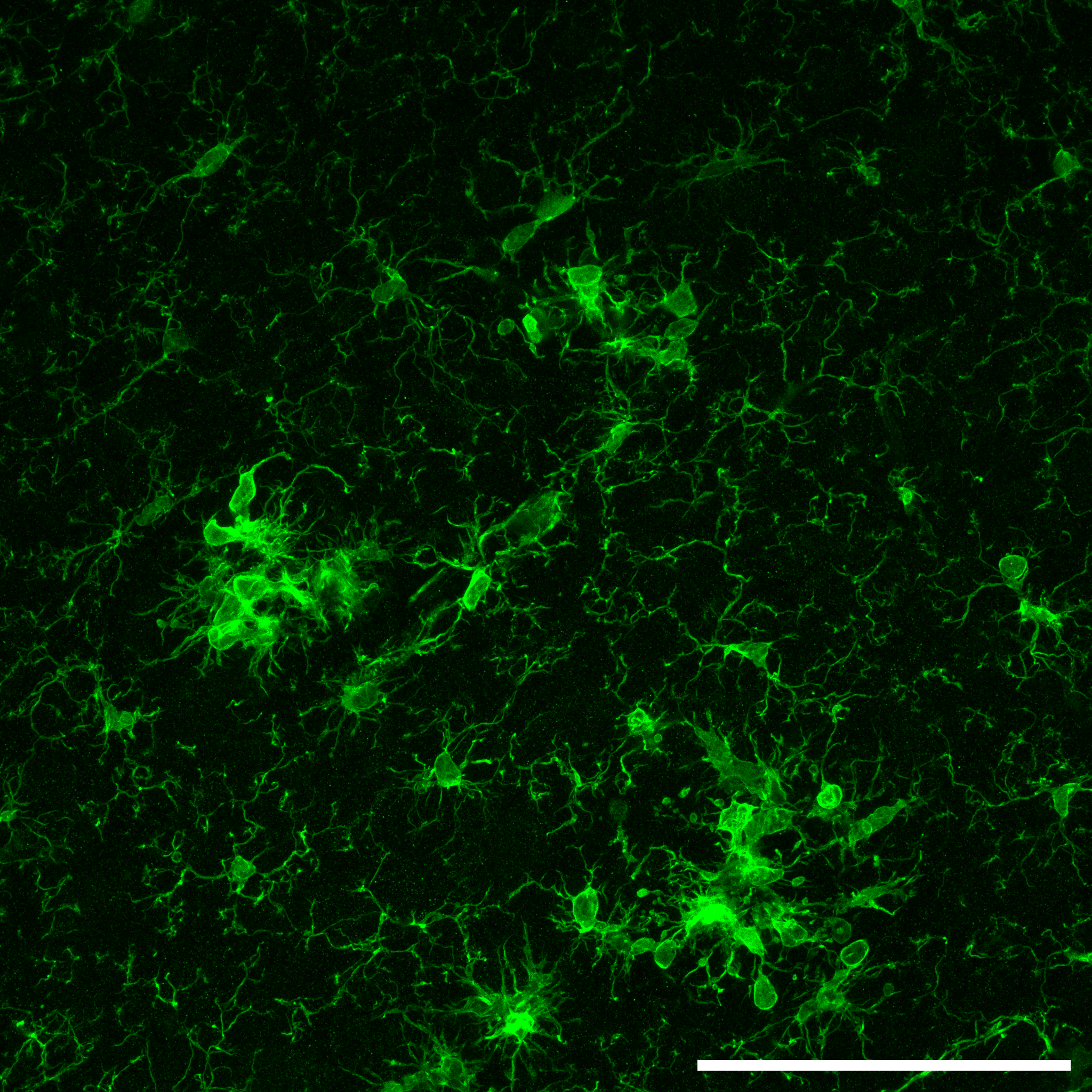

Supplement: Supplementary file 11 — Source data Fig. 4 [file 44321_2024_162_MOESM11_ESM.zip › Figure 4/4C/4C.APOE4_iba1.tif]

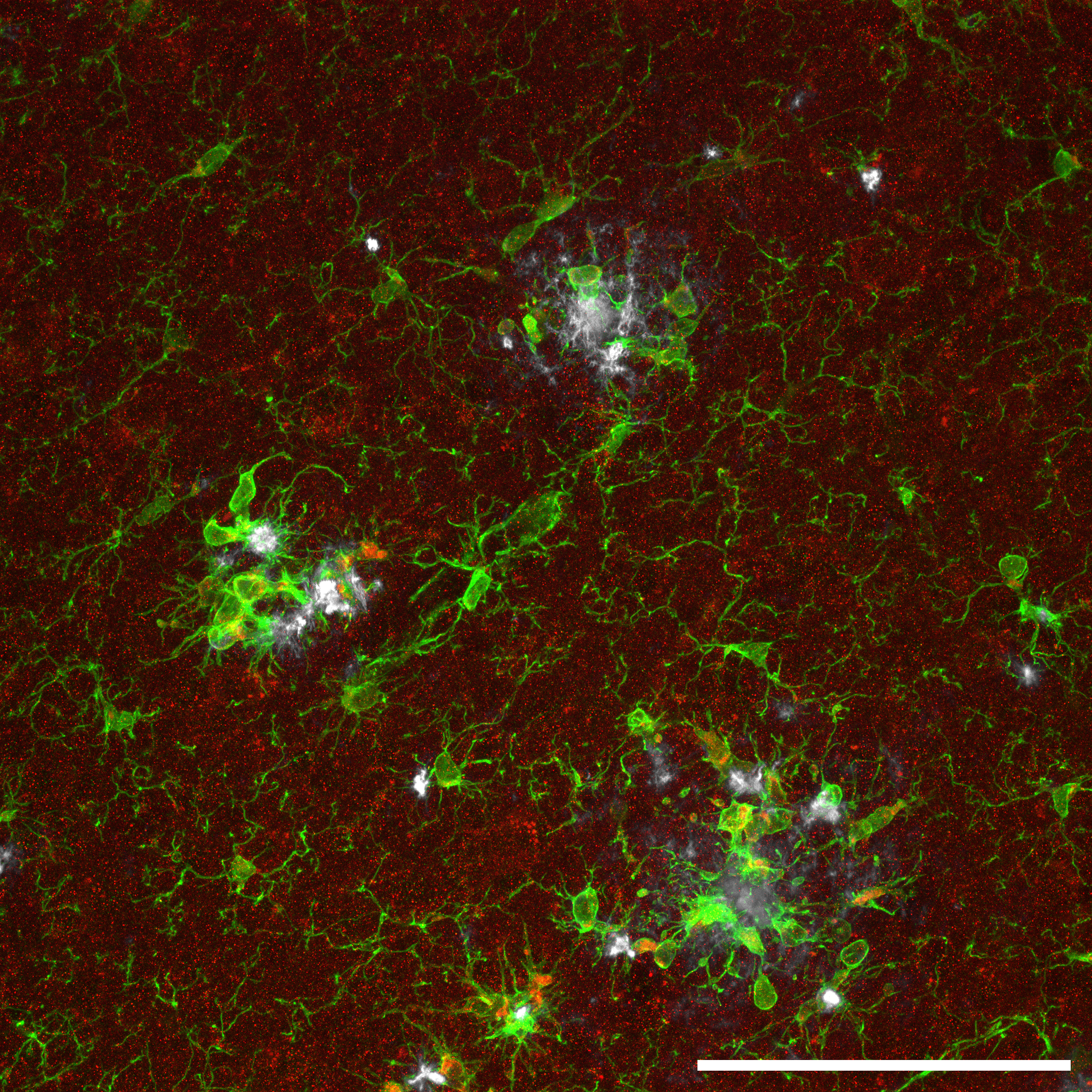

Supplement: Supplementary file 11 — Source data Fig. 4 [file 44321_2024_162_MOESM11_ESM.zip › Figure 4/4C/4C.APOE4_merge.tif]

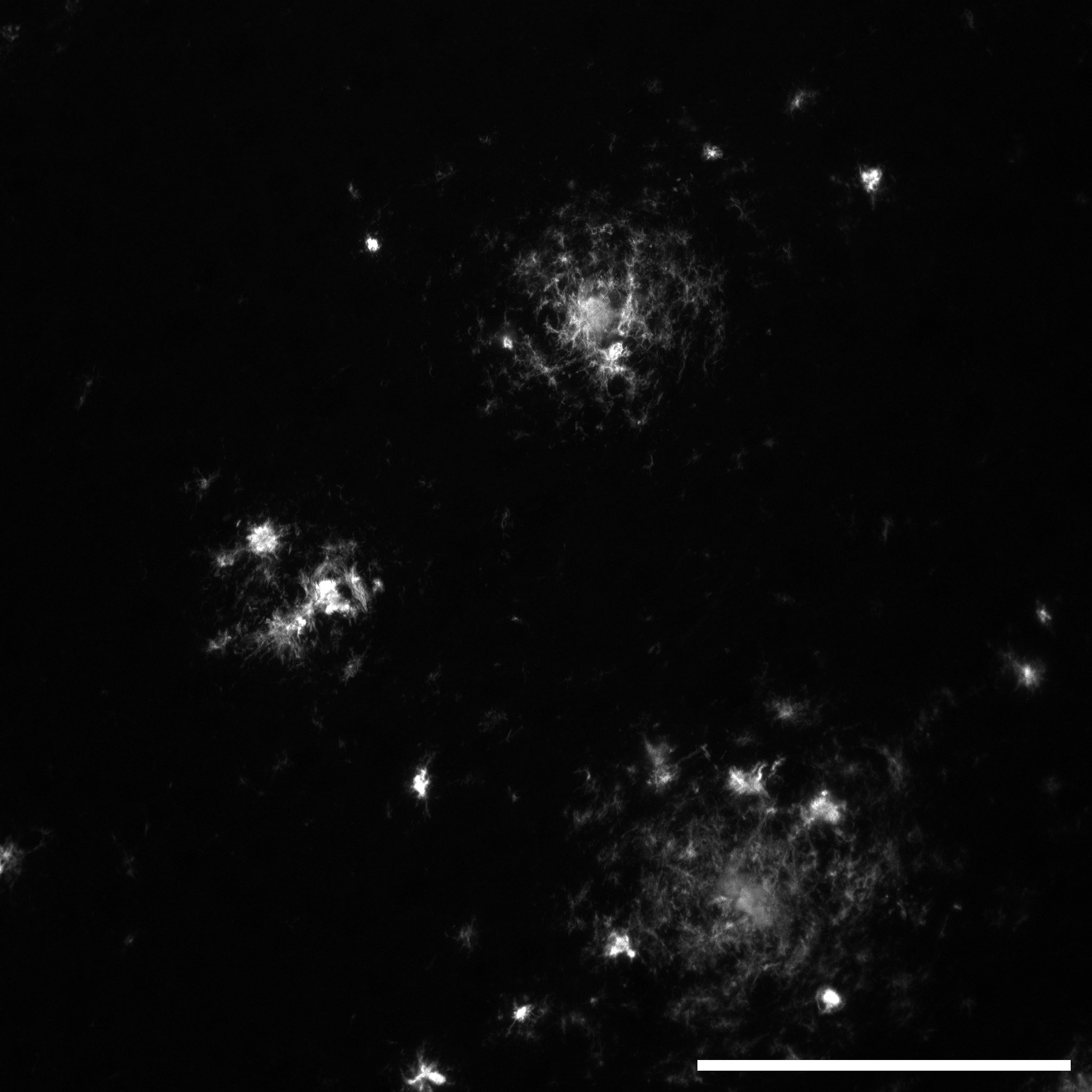

Supplement: Supplementary file 11 — Source data Fig. 4 [file 44321_2024_162_MOESM11_ESM.zip › Figure 4/4C/4C.APOE4_X34.tif]

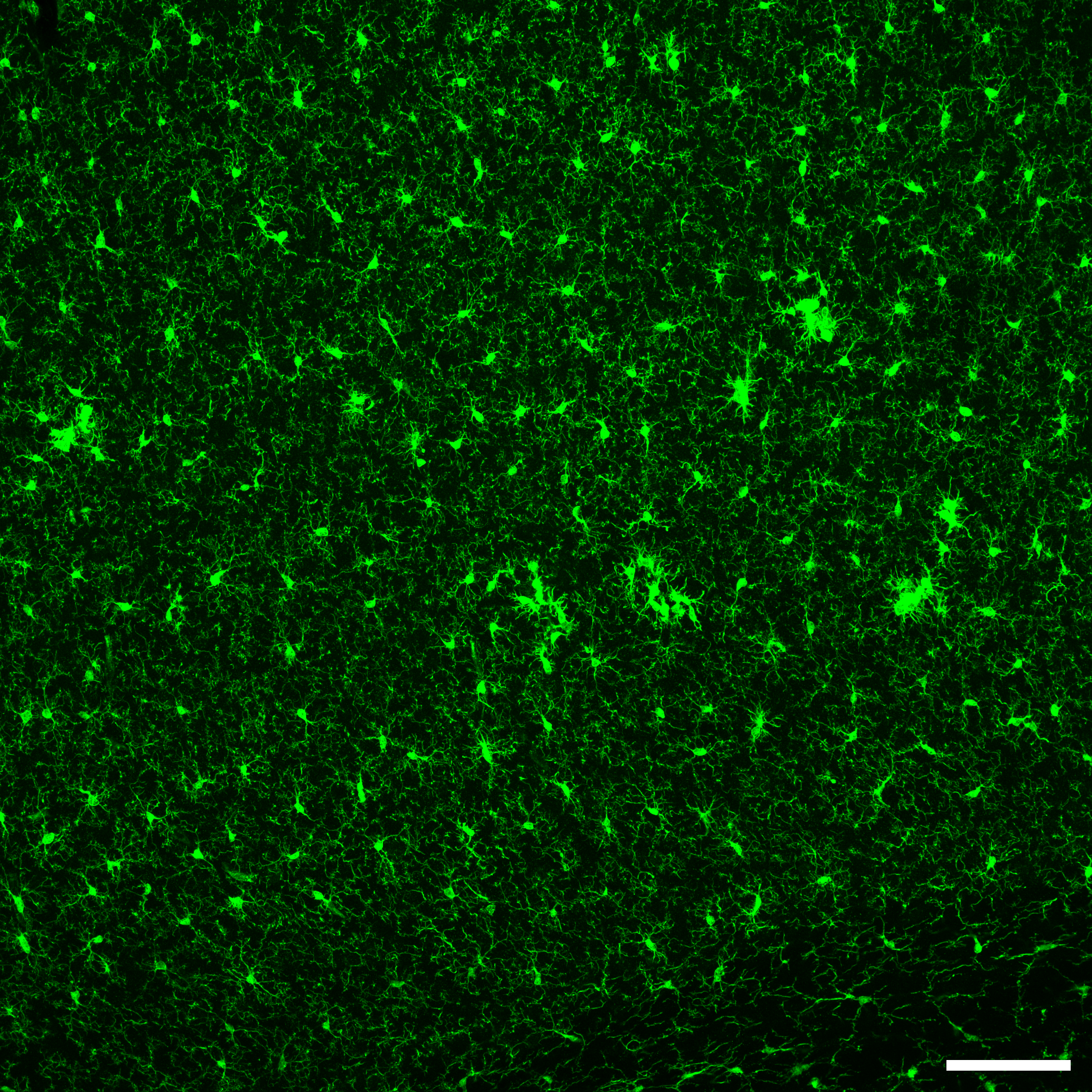

Supplement: Supplementary file 12 — Source data Fig. 5 [file 44321_2024_162_MOESM12_ESM.zip › Figure 5/5B/5B.APOE2_iba1.tif]

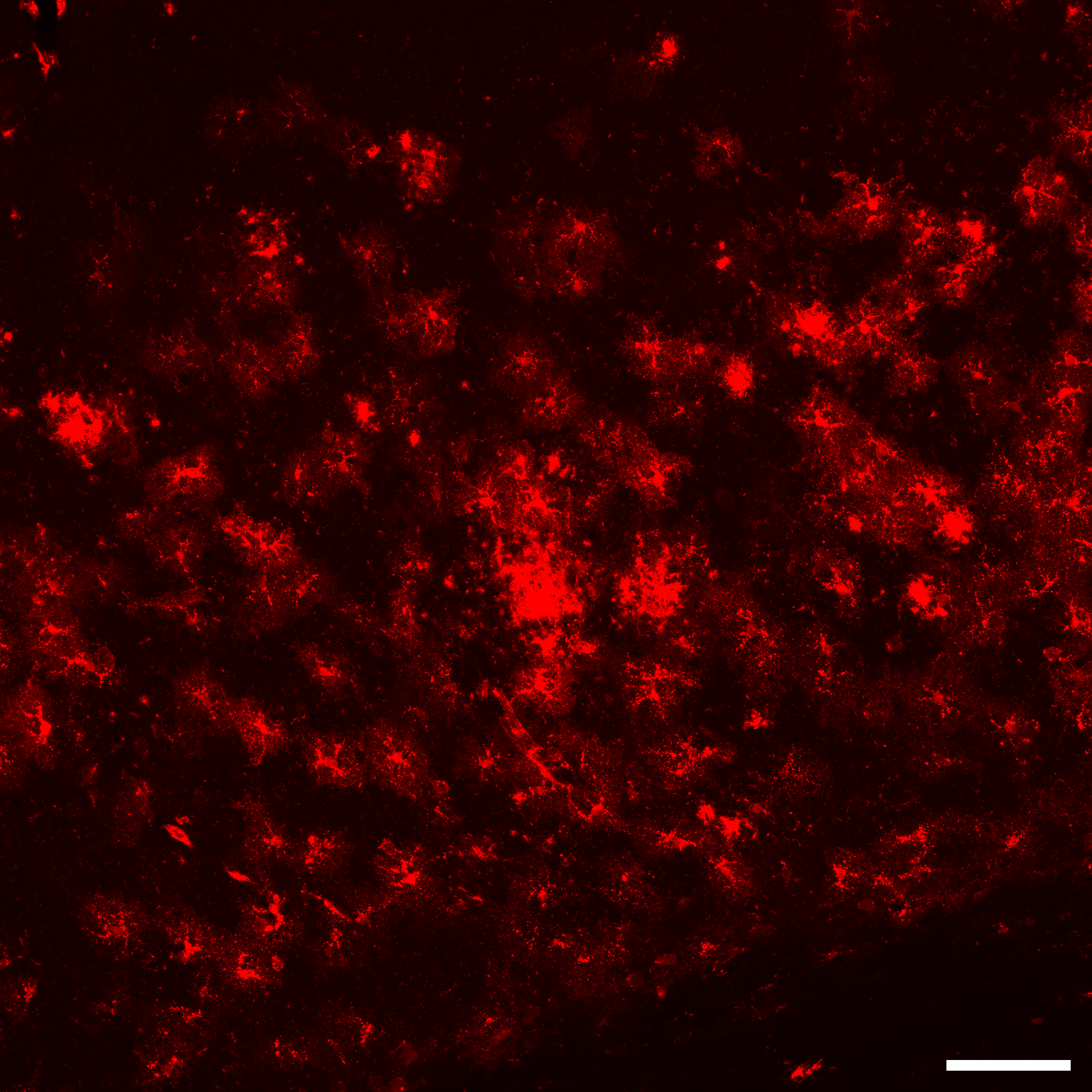

Supplement: Supplementary file 12 — Source data Fig. 5 [file 44321_2024_162_MOESM12_ESM.zip › Figure 5/5B/5B.APOE2_mcherry.tif]

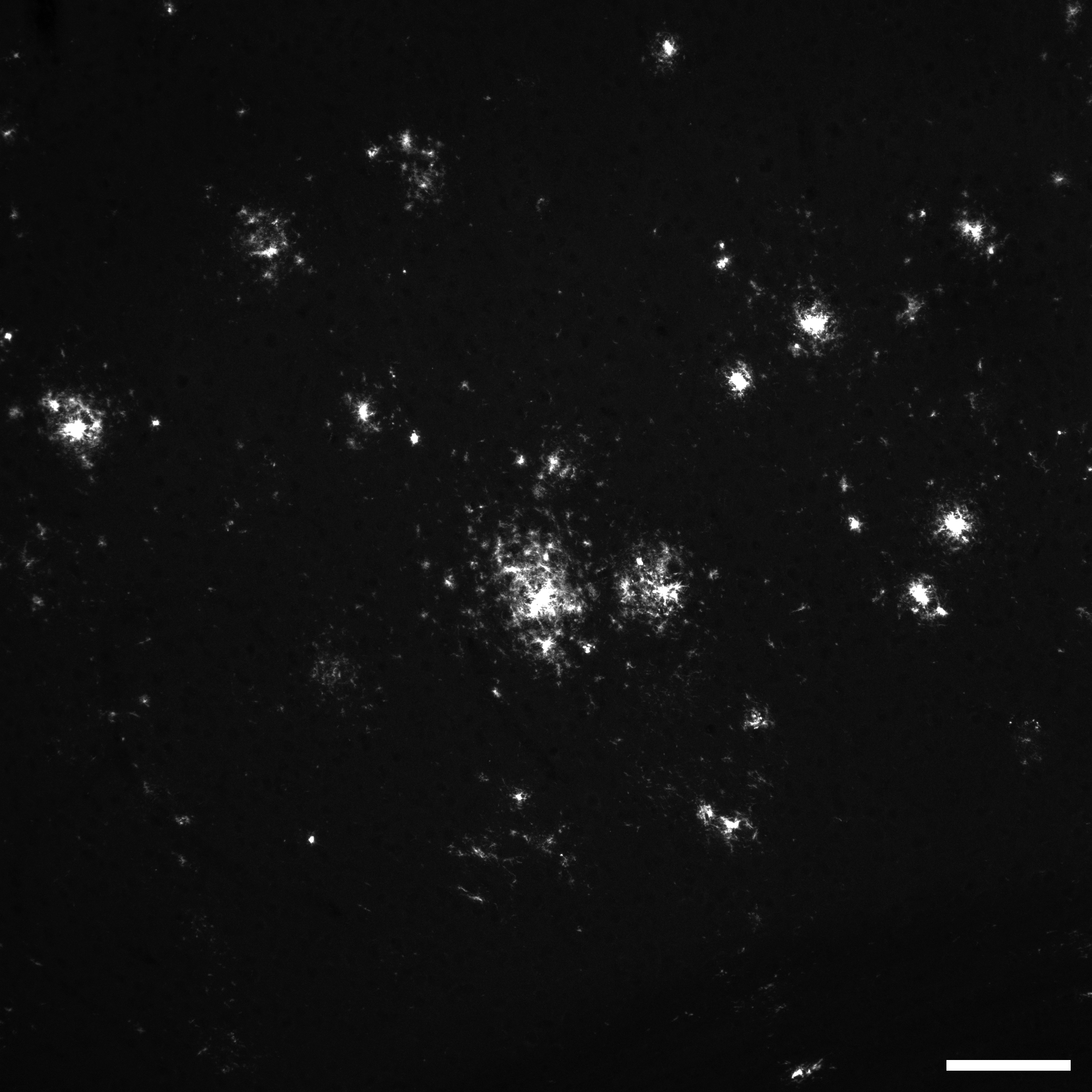

Supplement: Supplementary file 12 — Source data Fig. 5 [file 44321_2024_162_MOESM12_ESM.zip › Figure 5/5B/5B.APOE2_x-34.tif]

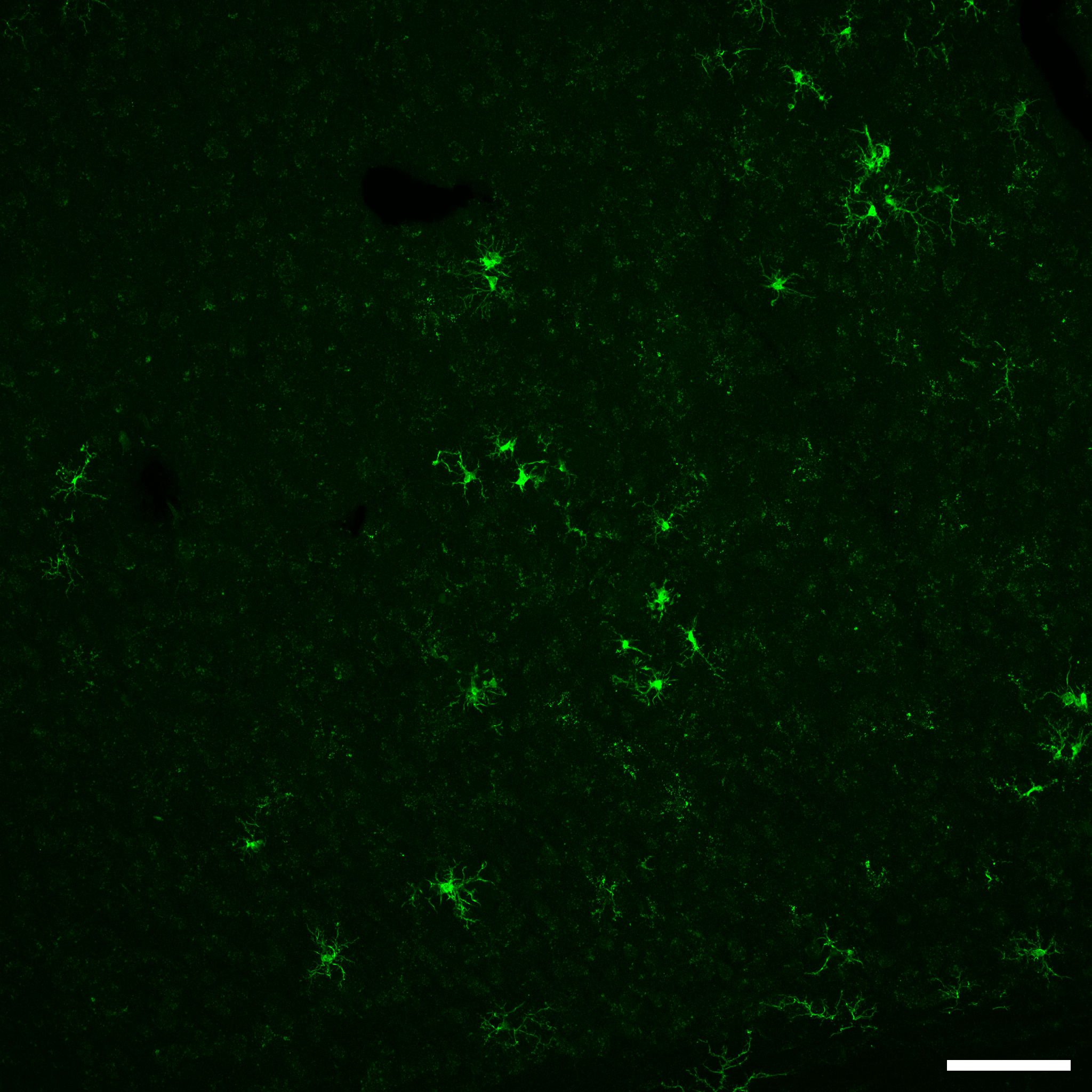

Supplement: Supplementary file 12 — Source data Fig. 5 [file 44321_2024_162_MOESM12_ESM.zip › Figure 5/5B/5B.APOE2plx_iba1.tif]

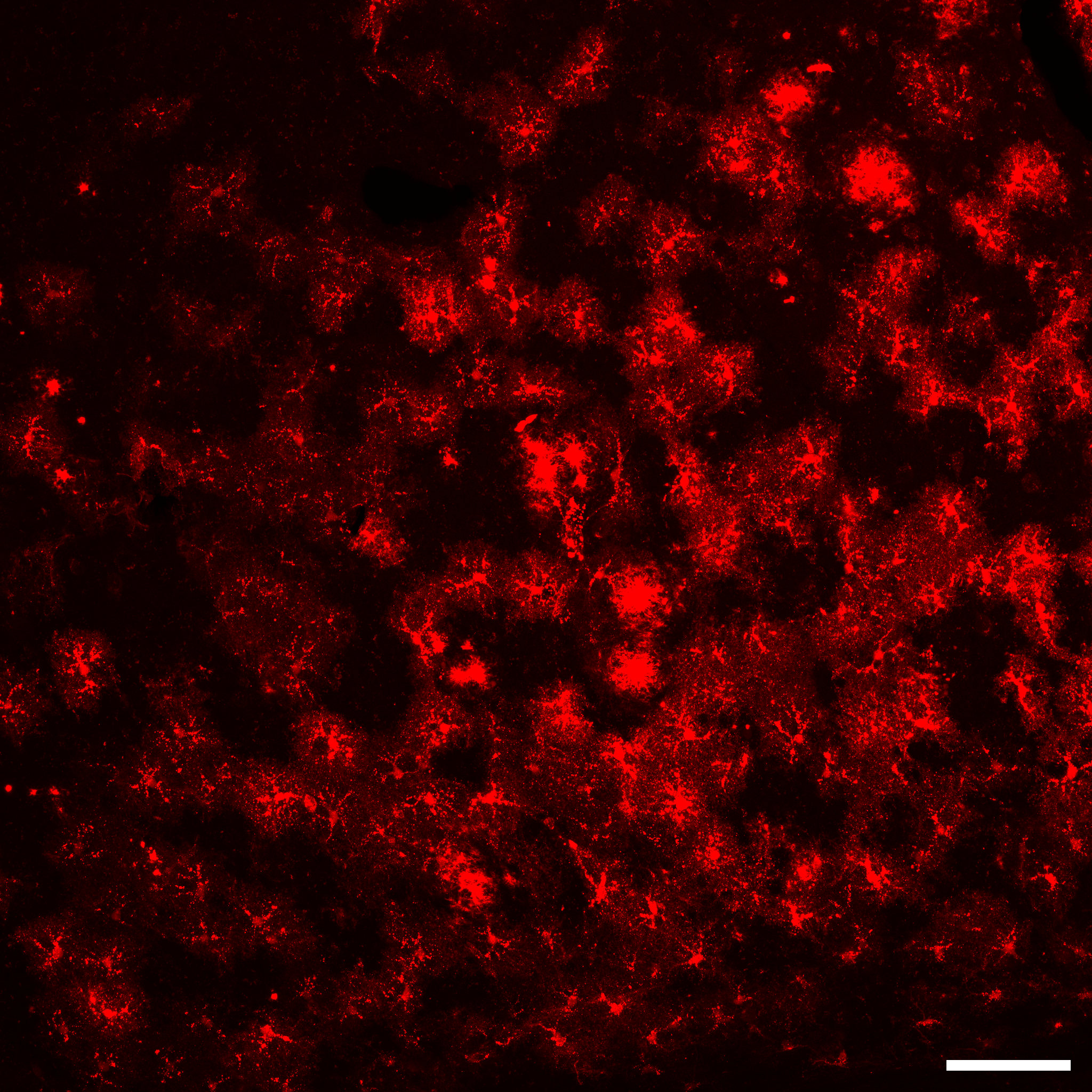

Supplement: Supplementary file 12 — Source data Fig. 5 [file 44321_2024_162_MOESM12_ESM.zip › Figure 5/5B/5B.APOE2plx_mcherry.tif]

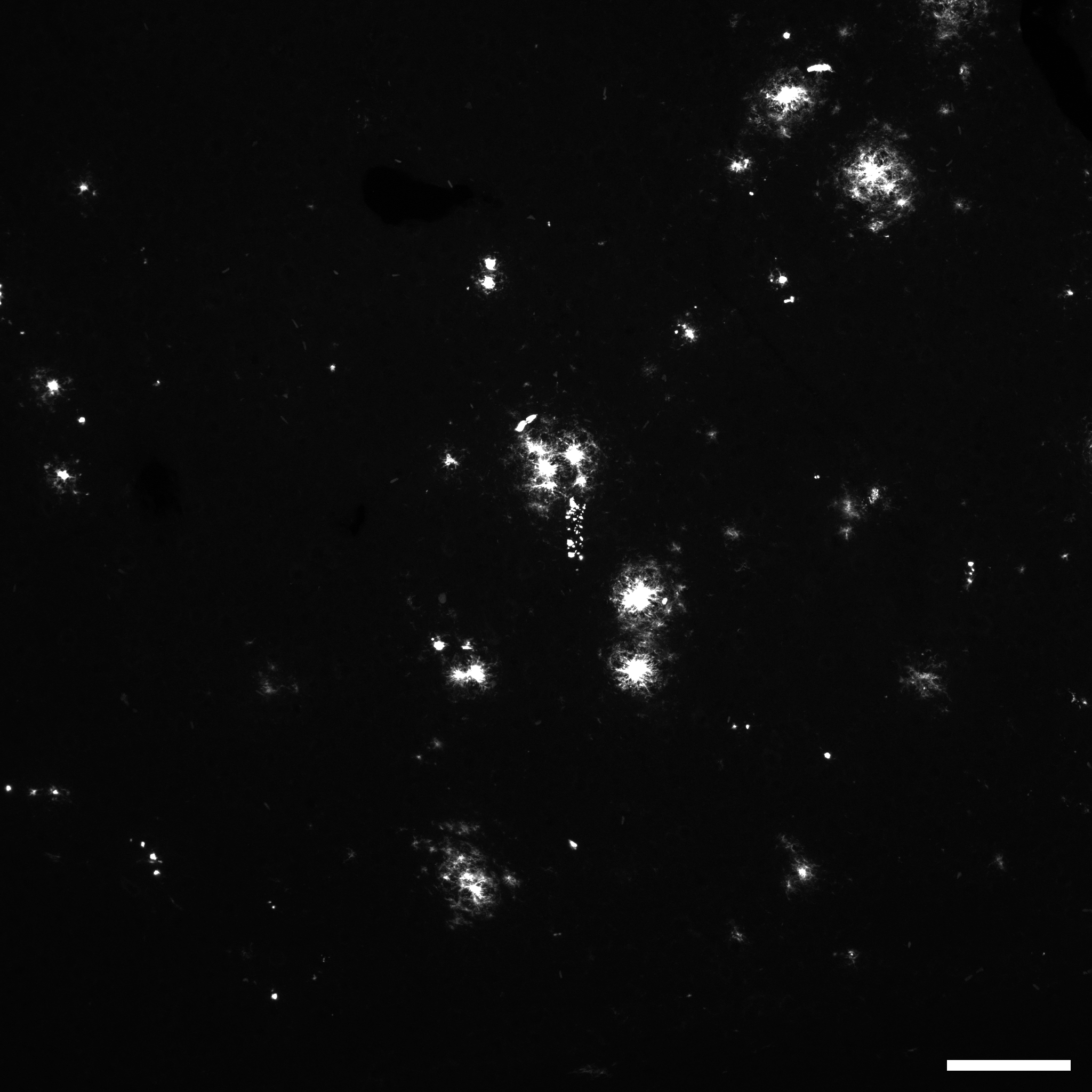

Supplement: Supplementary file 12 — Source data Fig. 5 [file 44321_2024_162_MOESM12_ESM.zip › Figure 5/5B/5B.APOE2plx_x-34.tif]

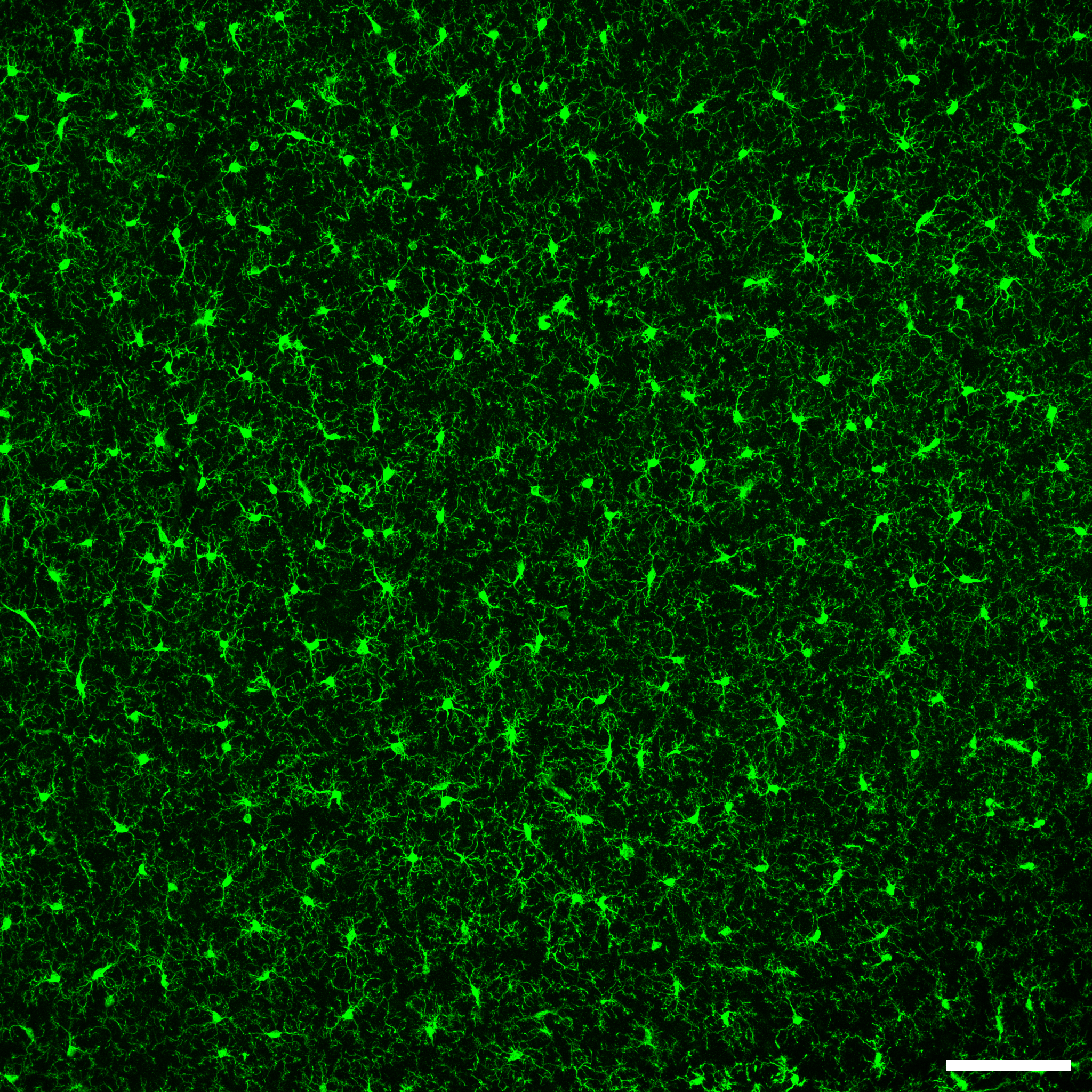

Supplement: Supplementary file 12 — Source data Fig. 5 [file 44321_2024_162_MOESM12_ESM.zip › Figure 5/5B/5B.APOE3_iba1.tif]

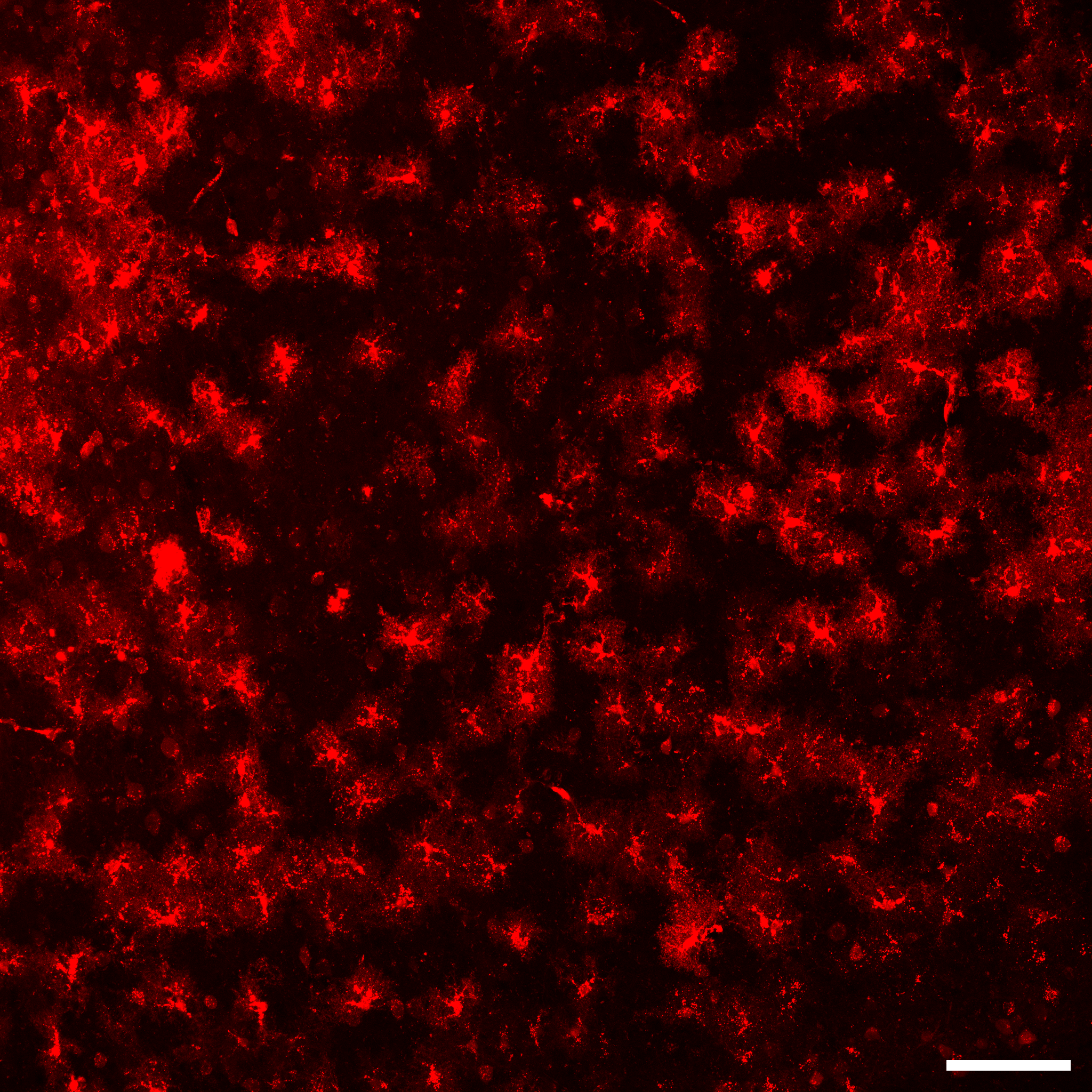

Supplement: Supplementary file 12 — Source data Fig. 5 [file 44321_2024_162_MOESM12_ESM.zip › Figure 5/5B/5B.APOE3_mcherry.tif]

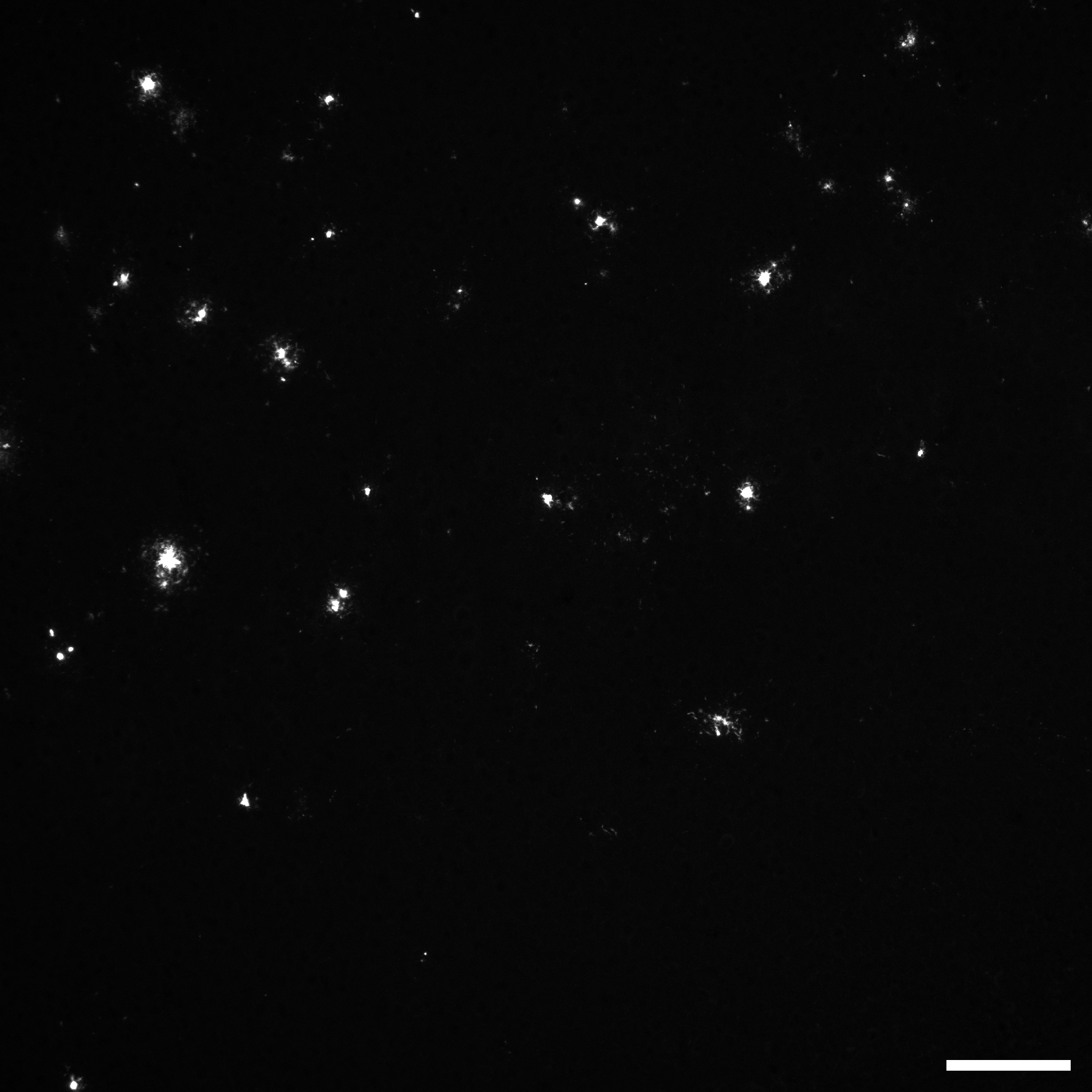

Supplement: Supplementary file 12 — Source data Fig. 5 [file 44321_2024_162_MOESM12_ESM.zip › Figure 5/5B/5B.APOE3_x-34.tif]

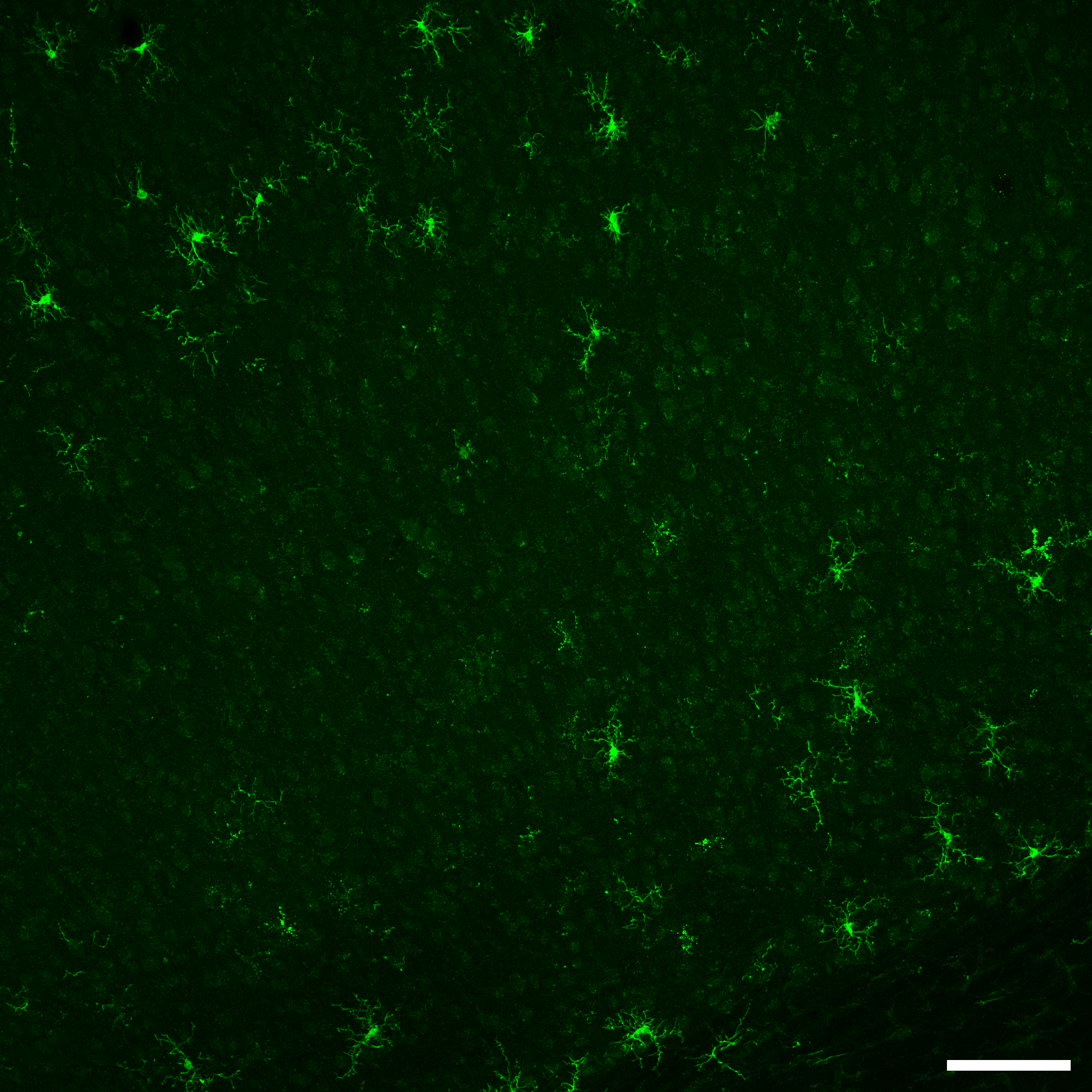

Supplement: Supplementary file 12 — Source data Fig. 5 [file 44321_2024_162_MOESM12_ESM.zip › Figure 5/5B/5B.APOE3plx_iba1.tif]

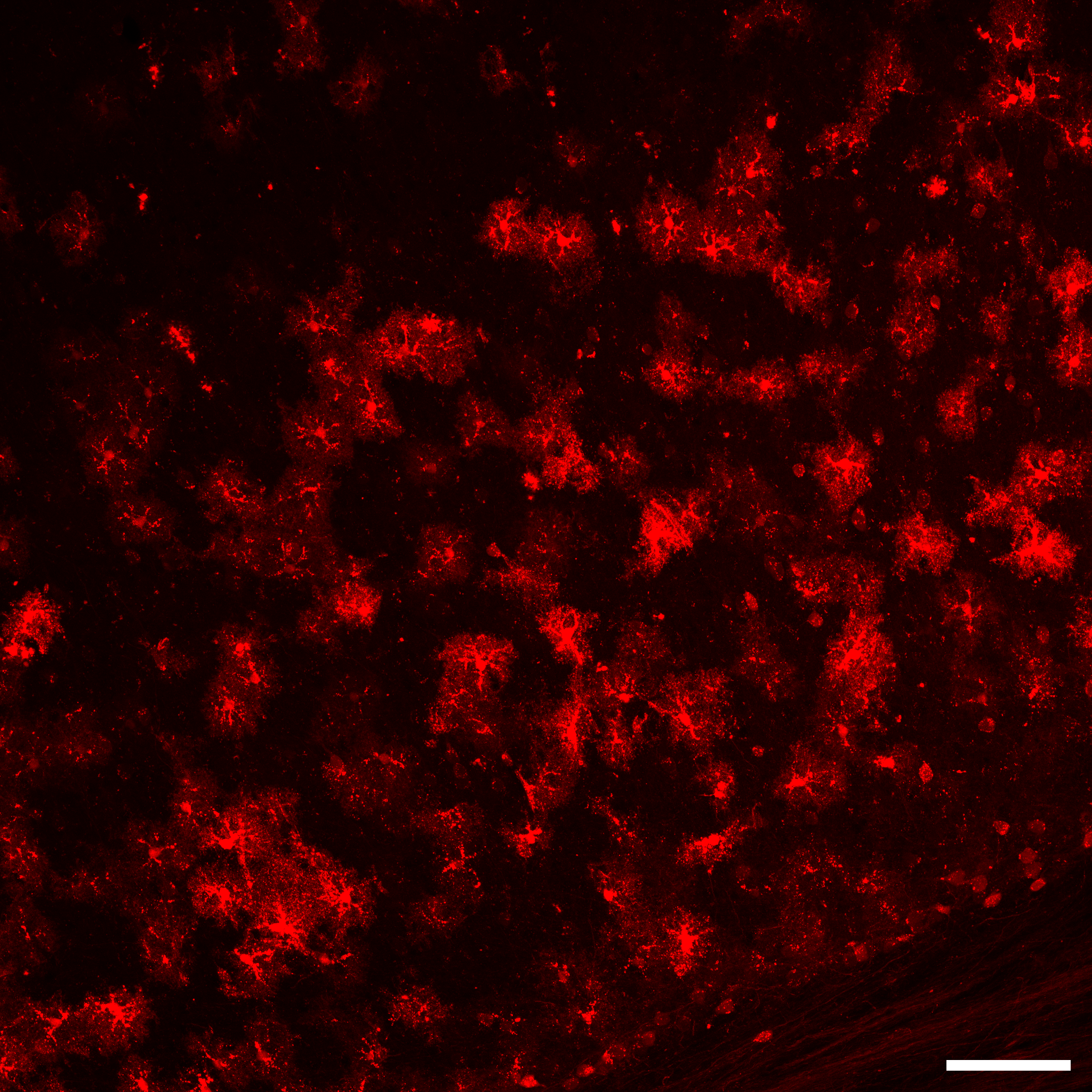

Supplement: Supplementary file 12 — Source data Fig. 5 [file 44321_2024_162_MOESM12_ESM.zip › Figure 5/5B/5B.APOE3plx_mcherry.tif]

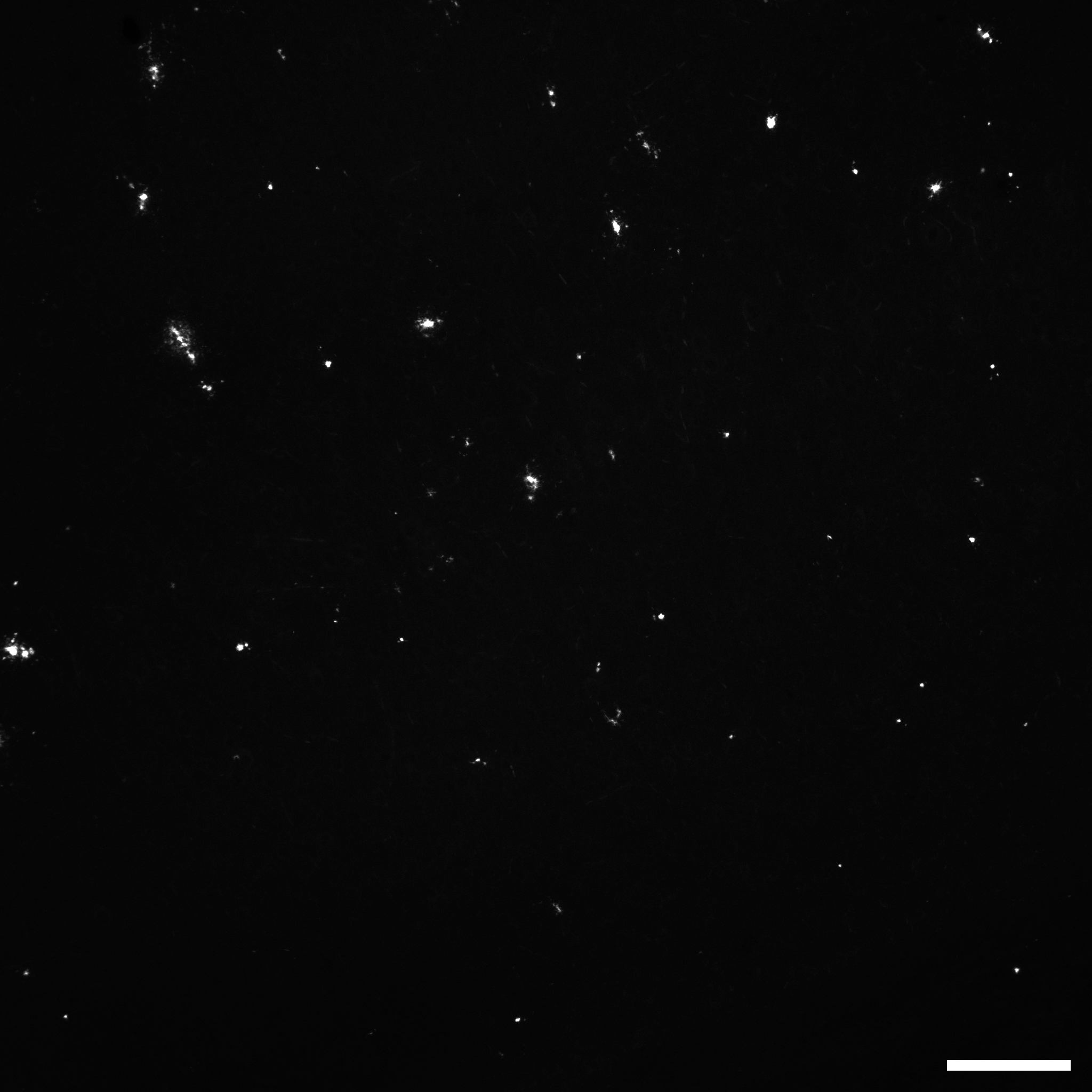

Supplement: Supplementary file 12 — Source data Fig. 5 [file 44321_2024_162_MOESM12_ESM.zip › Figure 5/5B/5B.APOE3plx_x-34.tif]

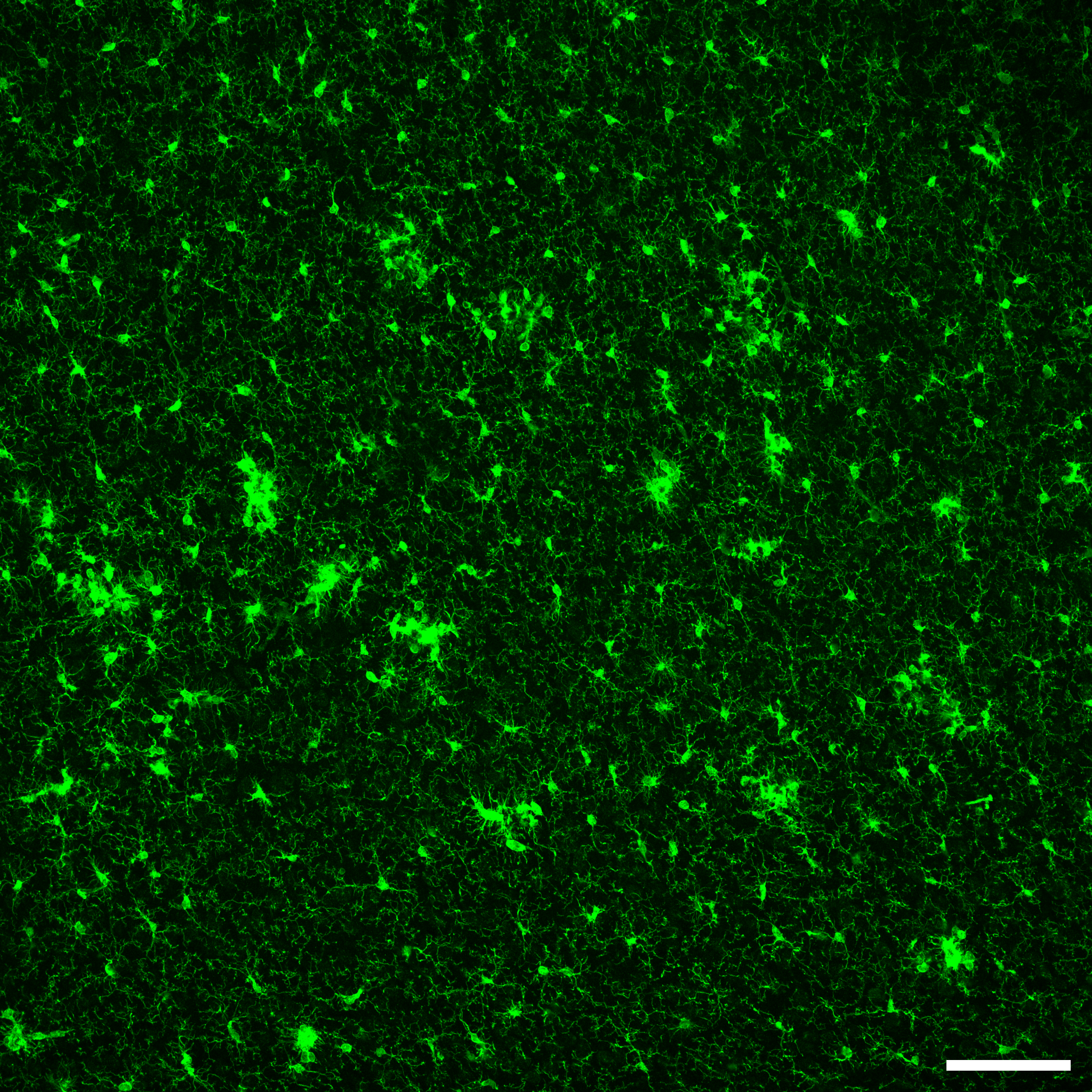

Supplement: Supplementary file 12 — Source data Fig. 5 [file 44321_2024_162_MOESM12_ESM.zip › Figure 5/5B/5B.APOE4_iba1.tif]

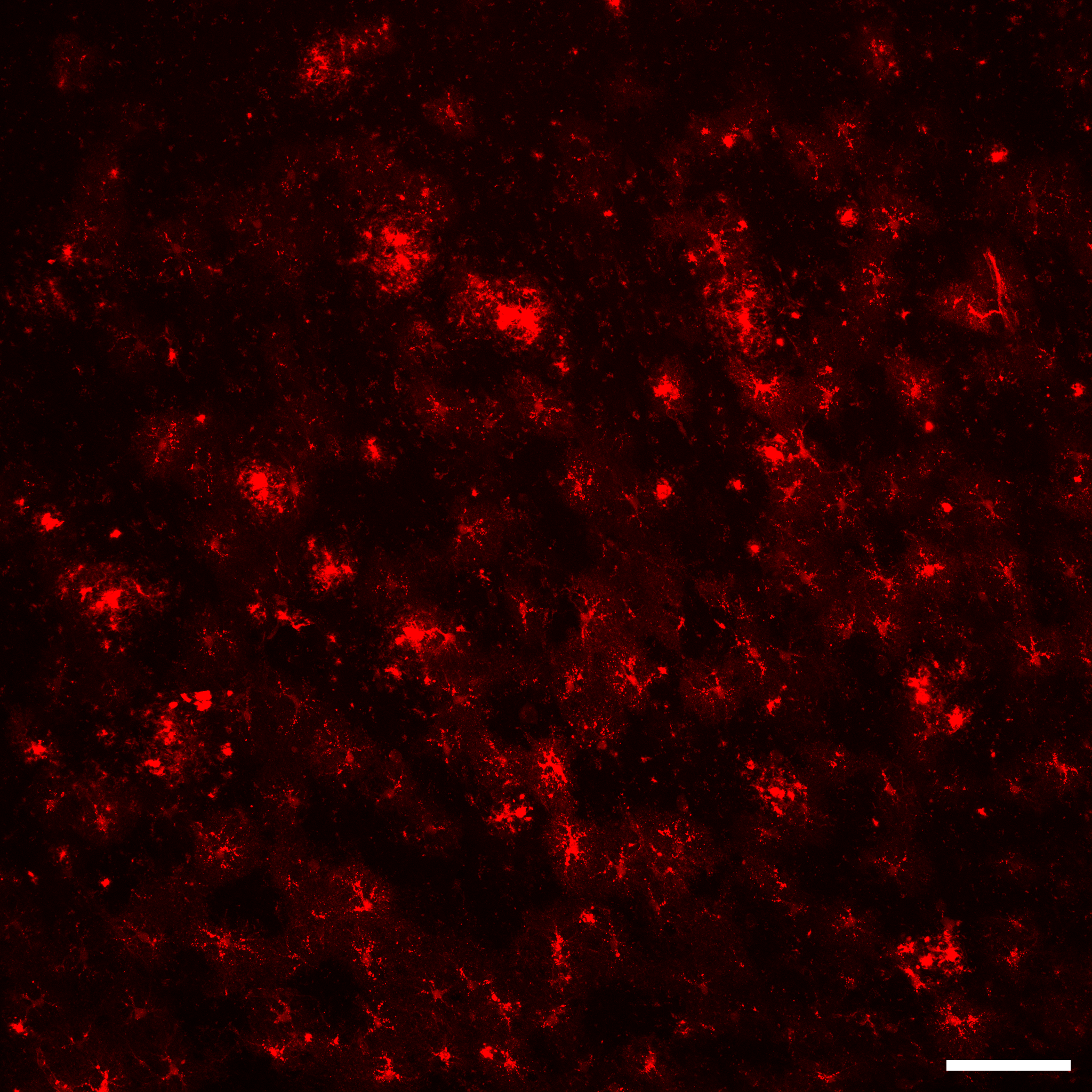

Supplement: Supplementary file 12 — Source data Fig. 5 [file 44321_2024_162_MOESM12_ESM.zip › Figure 5/5B/5B.APOE4_mcherry.tif]

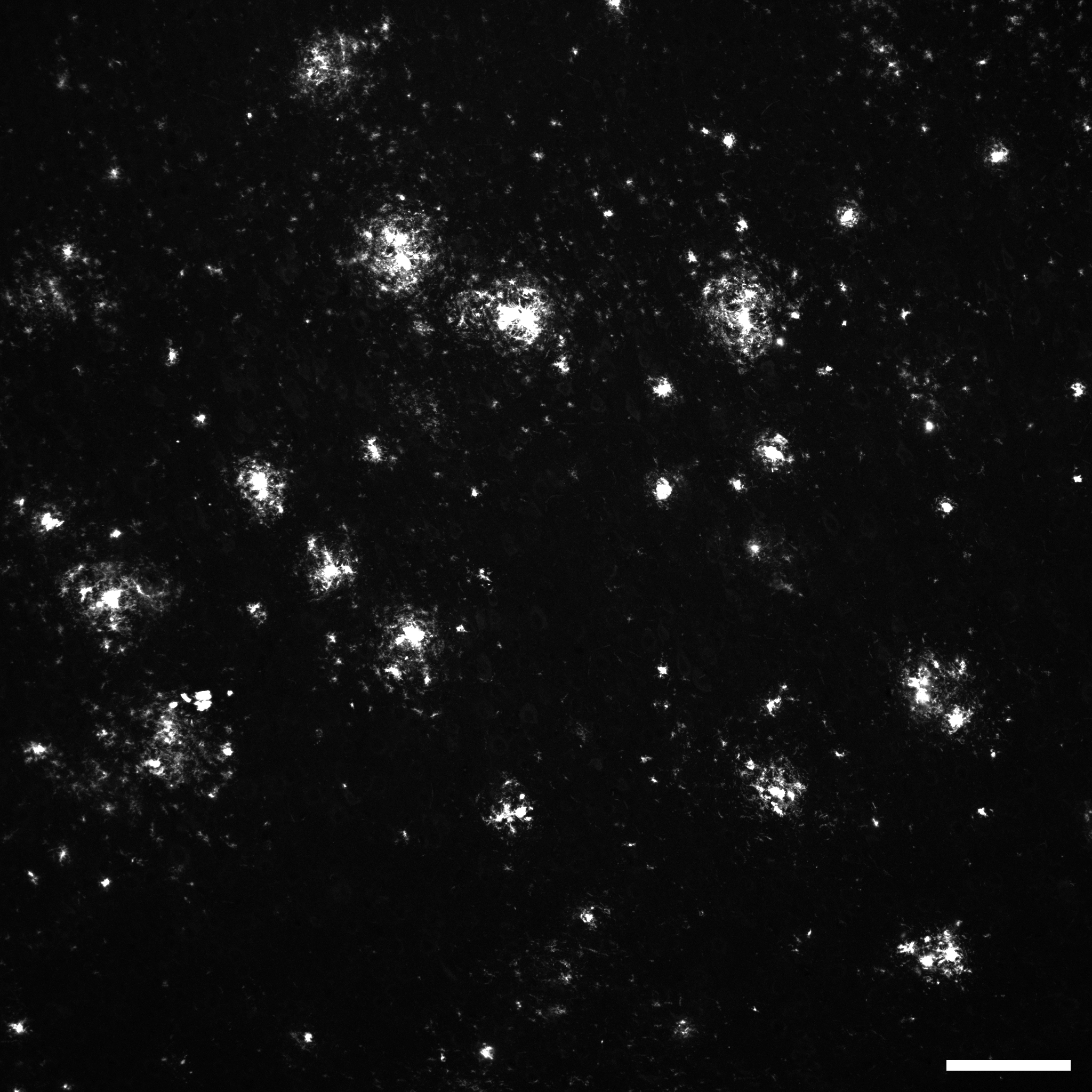

Supplement: Supplementary file 12 — Source data Fig. 5 [file 44321_2024_162_MOESM12_ESM.zip › Figure 5/5B/5B.APOE4_x-34.tif]

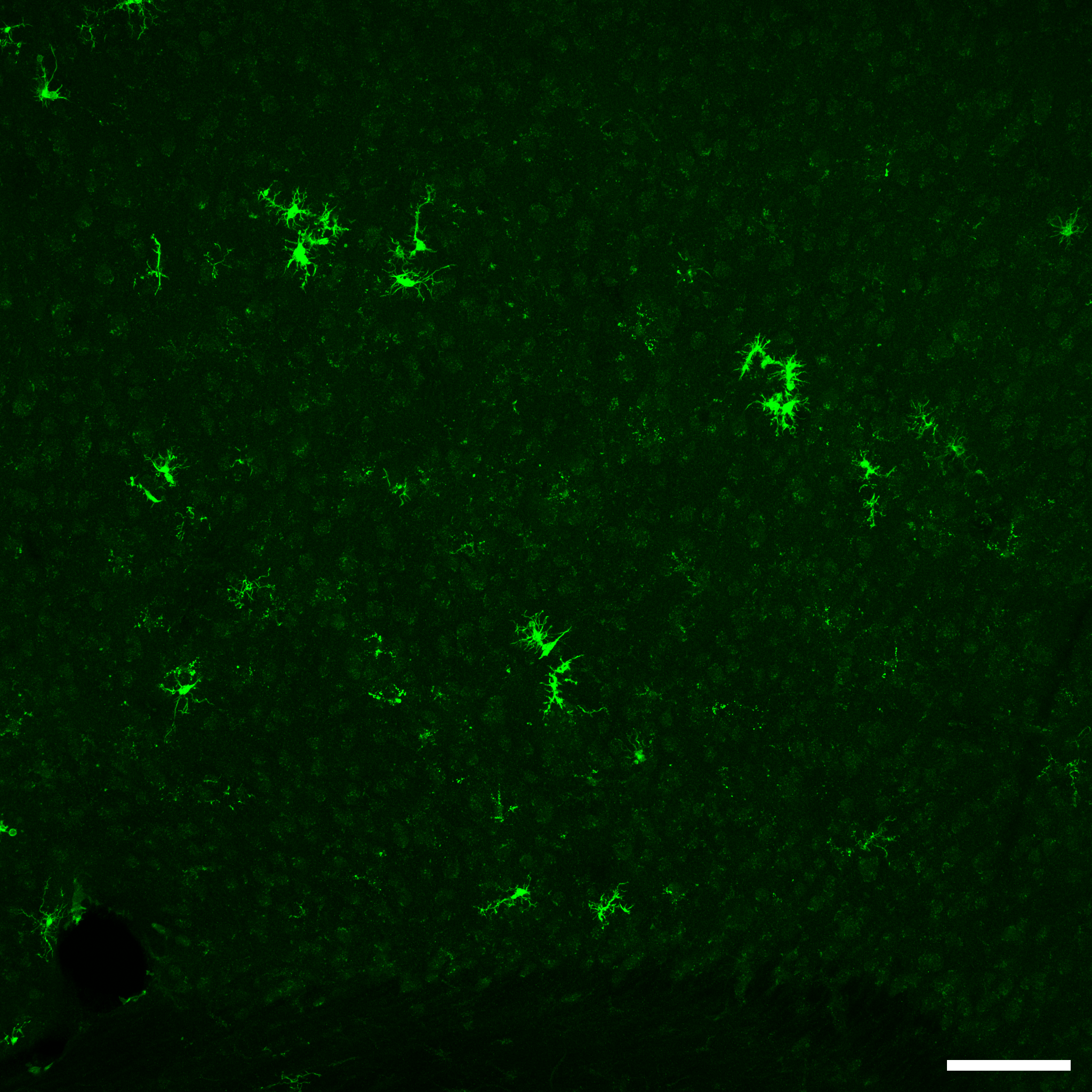

Supplement: Supplementary file 12 — Source data Fig. 5 [file 44321_2024_162_MOESM12_ESM.zip › Figure 5/5B/5B.APOE4plx_iba1.tif]

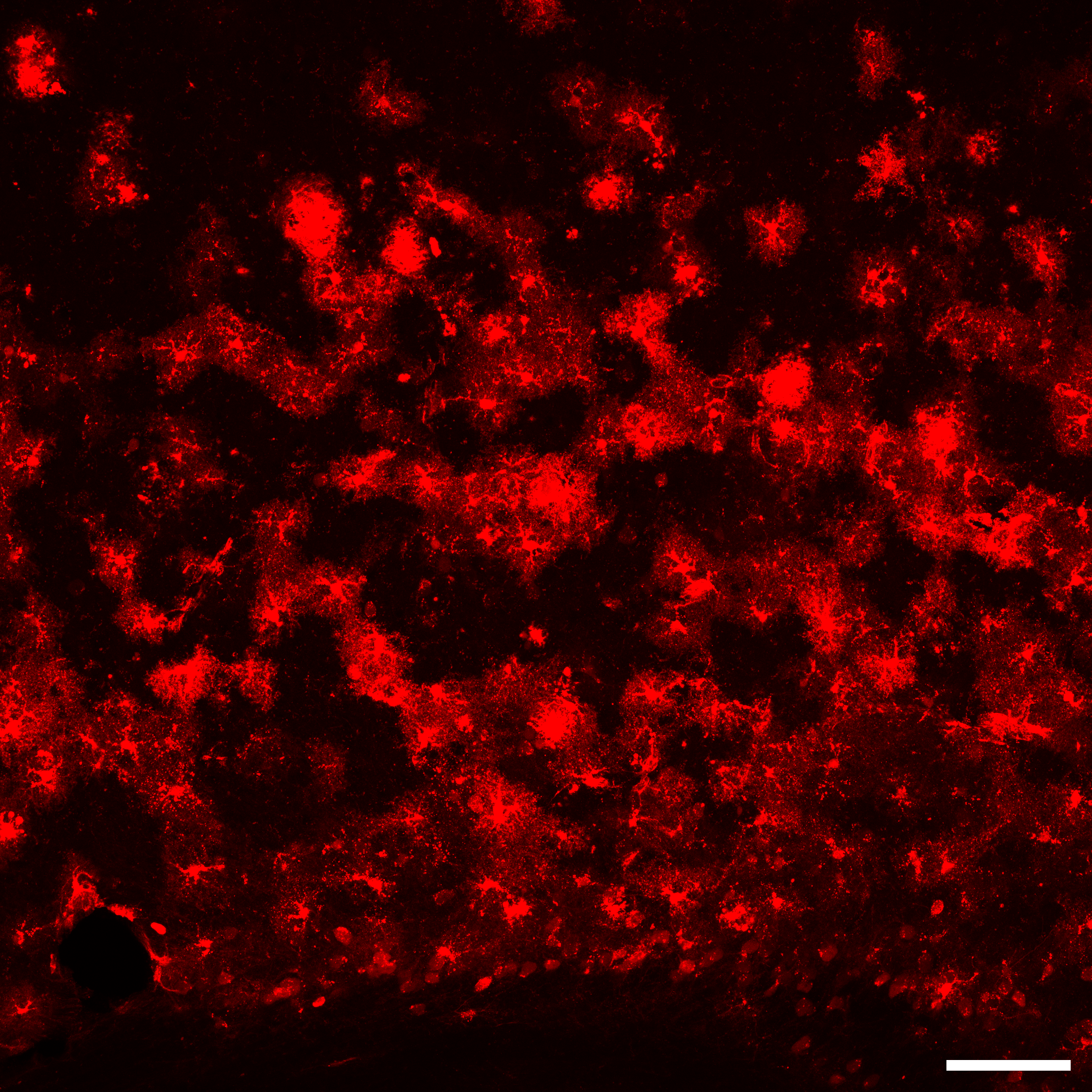

Supplement: Supplementary file 12 — Source data Fig. 5 [file 44321_2024_162_MOESM12_ESM.zip › Figure 5/5B/5B.APOE4plx_mcherry.tif]

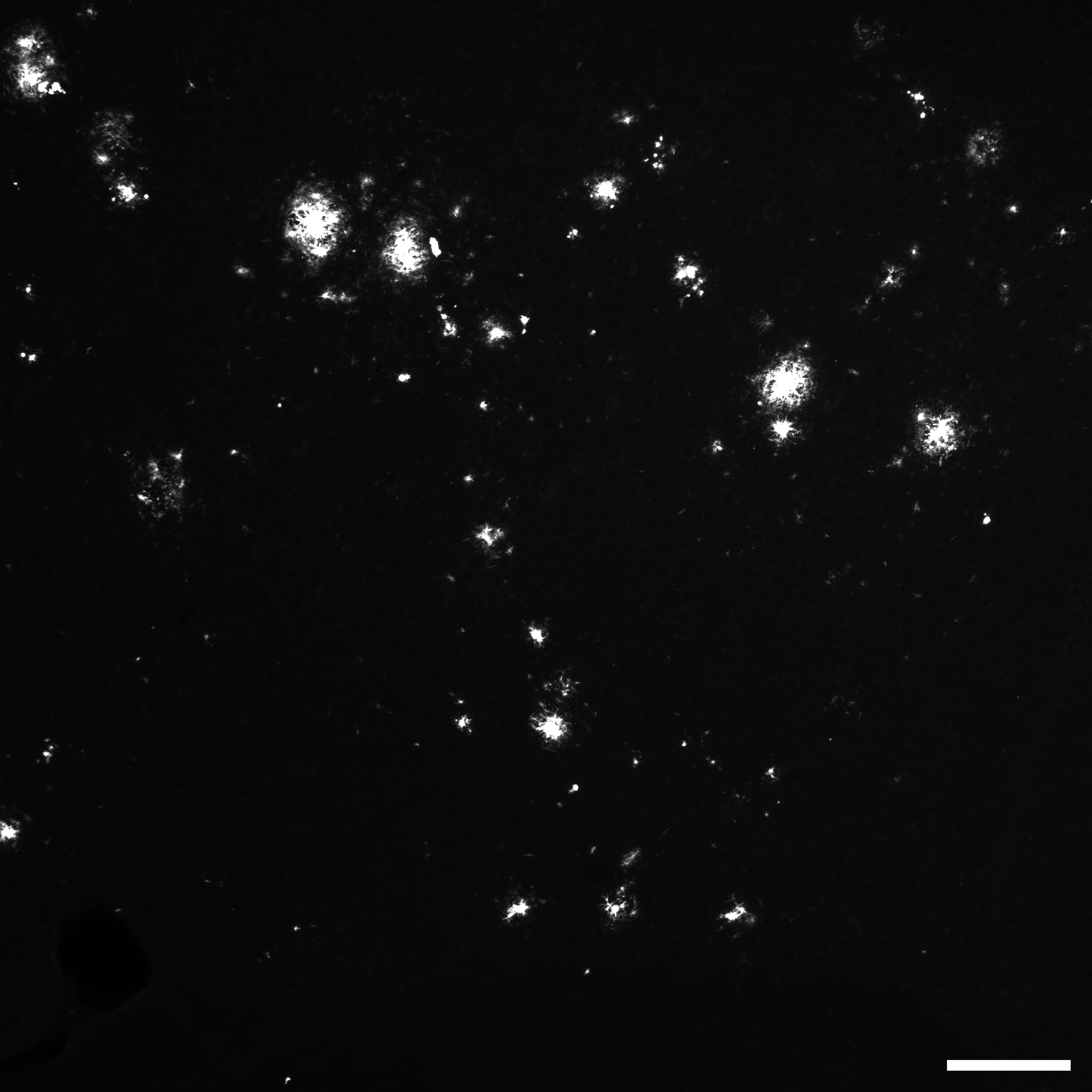

Supplement: Supplementary file 12 — Source data Fig. 5 [file 44321_2024_162_MOESM12_ESM.zip › Figure 5/5B/5B.APOE4plx_x-34.tif]
