# Supplementary material for: APOE from astrocytes restores Alzheimer’s Aβ-pathology and DAM-like responses in APOE deficient microglia
Source: EMBO Mol Med. 2024 Nov 11;16(12):3113–41. doi: 10.1038/s44321-024-00162-7 (PMC11628604; doi:10.1038/s44321-024-00162-7)
Supplement: Supplementary file 13 — Expanded View Figures [file 44321_2024_162_MOESM13_ESM.pdf]

Expanded View Figures

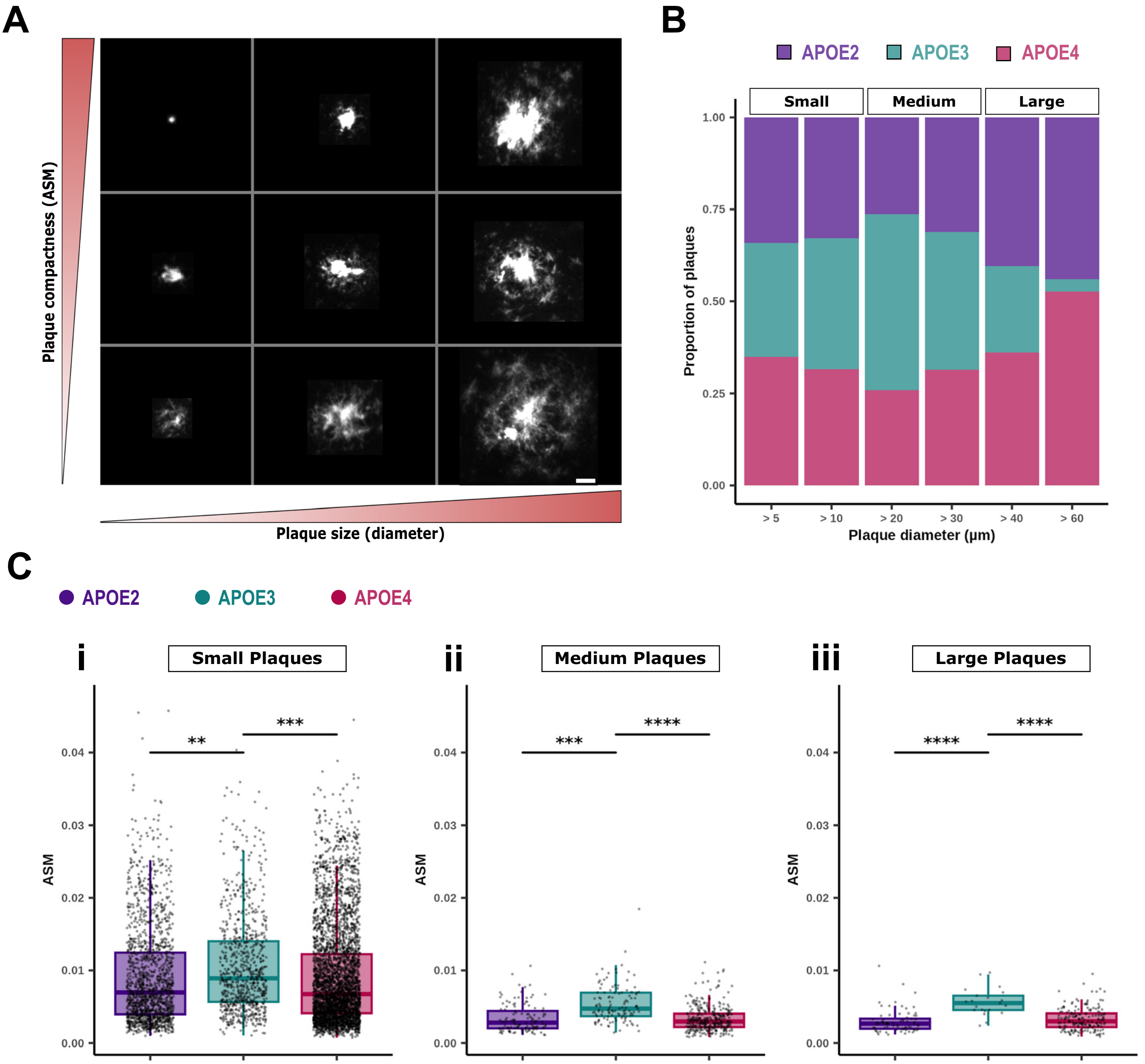

Figure EV1. Astrocyte-derived APOE modulates the size and compactness of fibrillar Aβ plaques.

(**A**) Composite tile showing individual X-34+ fibrillar plaques, with increasing plaque size (measured in diameter) along the x-axis and increasing compactness (measured in Angular Second Moment (ASM) value) along the y-axis. Higher ASM values correlate with higher compactness. Scale bar: 10 µm. (**B**) Stacked bar plot showing proportion of X-34+ fibrillar plaques from APOE2, APOE3 and APOE4 groups in different size categories (diameter in µm) along the x-axis. Lower limit for plaque size set to 5 µm in diameter. Size categories used: small (5–20 µm diameter), medium (20–40 µm diameter) and large (above 40 µm diameter). (**C**) Box plot showing ASM values as an estimate of plaque compactness in APOE2, APOE3 and APOE4 groups. Size categories used: (i) small (5–20 µm diameter), (ii) medium (20–40 µm diameter) and (iii) large (above 40 µm diameter). Data points show value for individual plaques ($n = 6$–10 mice per group. 3 FOV per mouse). In C(i), APOE2 vs APOE3 **$p = 0.0093$; APOE2 vs APOE4 $p = 0.547$; APOE3 vs APOE4 ***$p = 0.0003$. In C(ii), APOE2 vs APOE3 ***$p = 0.0001$; APOE2 vs APOE4 $p = 0.9818$; APOE3 vs APOE4 ****$p < 0.0001$. In C(iii), APOE2 vs APOE3 ****$p < 0.0001$; APOE2 vs APOE4 $p = 0.471$; APOE3 vs APOE4 ****$p < 0.0001$. Statistical tests: Data presented as median and interquartile range ± values within 1.5 times the interquartile range (**C**). Minima and maxima for box plots in C(i) APOE2 (0.001, 0.0457), APOE3 (0.0011, 0.0501), APOE4 (0.0007, 0.0565); in C(ii) APOE2 (0.0011, 0.0106), APOE3 (0.0015, 0.0184), APOE4 (0.0008, 0.0111); in C(iii) APOE2 (0.0012, 0.0106), APOE3 (0.0024, 0.0097), APOE4 (0.0009, 0.0095). Linear mixed effects model with Tukey's HSD test in (**C**) done at mouse sample level.

© The Author(s)

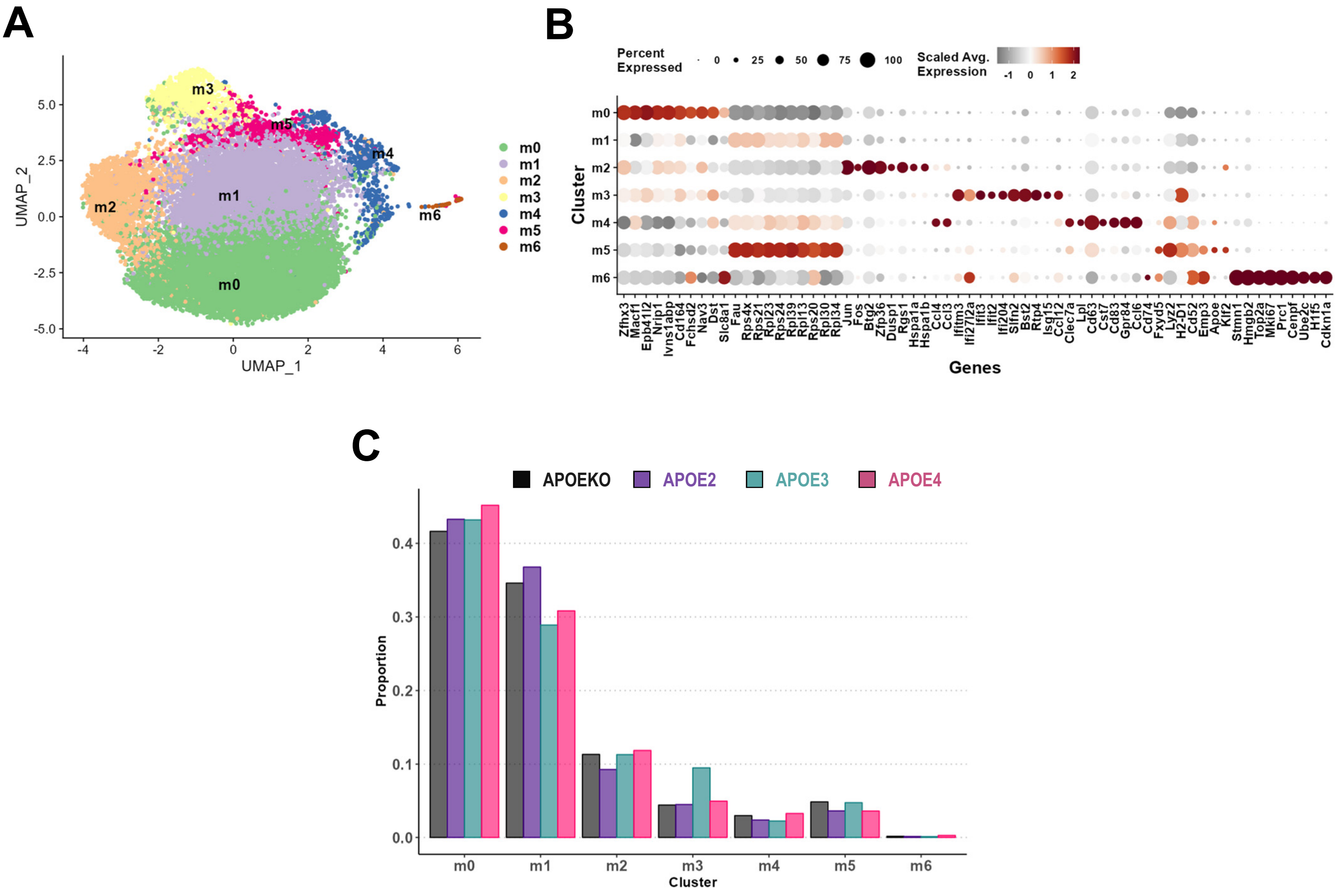

Figure EV2. Astrocyte-derived APOE influences the expression of microglial cell state markers.

(**A**) UMAP plot showing 18,569 APOE-deficient microglia (Cd11b+/Cd45-low) sorted from 6 months old mouse brains from the four experimental groups ($n = 2$ or 3 mice per group). Different sub-populations identified through unbiased clustering have been assigned cluster numbers. (**B**) Dot plot showing the top 10 differentially expressed genes in each cluster. Colour scale indicates normalized expression level, scaled per gene (z-score). Dot size indicates percentage of cells, in each cluster, expressing the gene. (**C**) Bar plot showing the proportion of microglia from each experimental group present in the different clusters.

 © The Author(s)

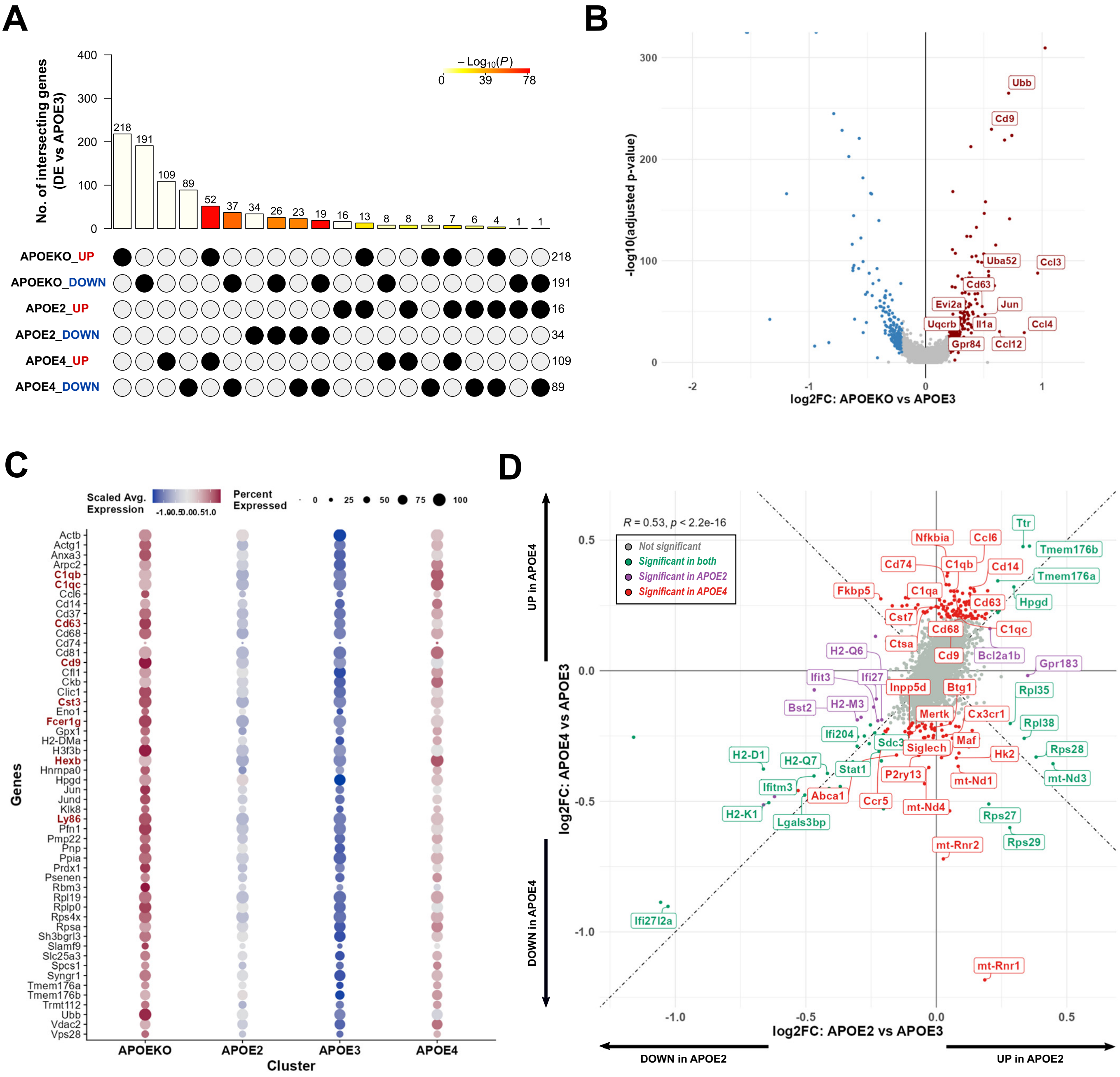

Figure EV3. Astrocyte-derived APOE modulates gene expression in microglia.

(**A**) Upset plot showing number of differentially expressed genes (UP or DOWN) in microglia from each experimental group, compared to APOE3 group. Bar plot shows the number of differentially expressed genes (DEGs) common between different DE analyses (overlapping sets indicated by black dots in column below the bars). Bars are coloured according to statistical significance of intersection of DEGs. (**B**) Volcano plot showing differentially expressed genes between APOEKO telencephalon astrocytes and APOE3 expressing telencephalon astrocytes. Data points for significant genes are coloured (Red for UP and blue for DOWN). Significance assigned based on |Log2(Fold Change)| > 0.2 and adjusted *p*-value < 0.05. Genes discussed in text are indicated in red. (**C**) Dot plot showing expression of the 52 genes (Fig. 2A) commonly upregulated in APOEKO and APOE4 microglia, split by experimental groups. Colour scale indicates normalized expression level, scaled per gene (z-score). Dot size indicates percentage of cells, in each group, expressing the gene. Plaque-induced genes (PIGs) (Chen et al, 2020) are coloured in red on y-axis. (**D**) Quadrant plot comparing differential expression of genes in microglia in APOE2 vs APOE3 (along x-axis) and in APOE4 vs APOE3 (along y-axis). Colours in legend key indicate statistical significance of genes up- or downregulated in APOE2 or APOE4 or both. Significance of differentially expressed genes based on |Log2(Fold Change)| > 0.2 and adjusted *p*-value < 0.05). Pearson's correlation, R = 0.52. Statistical tests: MAST differential expression test in (**B**), (**C**) and (**D**), *p*-values were adjusted with Bonferroni correction based on the total number of genes in the dataset.

© The Author(s)
